# Lung‐Specific mRNA Delivery by Ionizable Lipids with Defined Structure‐Function Relationship and Unique Protein Corona Feature

**DOI:** 10.1002/advs.202416525

**Published:** 2025-02-18

**Authors:** Xiaoyan He, Runyuan Wang, Yan Cao, Yan Ding, Yan Chang, Haoru Dong, Rong Xie, Guisheng Zhong, Huiying Yang, Jianfeng Li

**Affiliations:** ^1^ School of Life Science and Technology & State Key Laboratory of Advanced Medical Materials and Devices ShanghaiTech University Shanghai 201210 China; ^2^ Department of Neurosurgery Huashan Hospital Fudan University Shanghai 200040 China; ^3^ iHuman Institute ShanghaiTech University Shanghai 201210 China; ^4^ Department of Pharmacy Huashan Hospital Fudan University Shanghai 200040 China

**Keywords:** lipid nanoparticle, lung‐targeted, mRNA delivery, protein corona, structure‐function relationship

## Abstract

Targeted delivery of mRNA with lipid nanoparticles (LNPs) holds great potential for treating pulmonary diseases. However, the lack of rational design principles for efficient lung‐homing lipids hinders the prevalence of mRNA therapeutics in this organ. Herein, the combinatorial screening with structure‐function analysis is applied to rationalize the design strategy for nonpermanently charged lung‐targeted ionizable lipids. It is discovered that lipids carrying N‐methyl and secondary amine groups in the heads, and three tails originated from epoxyalkanes, exhibiting superior pulmonary selectivity and efficiency. Representative ionizable lipids with systematically variation in chemical structures are selected to study the well‐known but still puzzling “protein corona” adsorbed on the surface of LNPs. In addition to the commonly used corona‐biomarker vitronectin, other arginine‐glycine‐aspartic acid (RGD)‐rich proteins usually involved in collagen‐containing extracellular matrix, such as fibrinogen and fibronectin have also been identified to have a strong correlation with lung tropism. This work provides insight into the rational design of lung‐targeting ionizable lipids and reveals a previously unreported potential function of RGD‐rich proteins in the protein corona of lung‐homing LNPs.

## Graphical abstract

A novel structure‐function relationship is identified for lung‐targeting ionizable lipids with nonpermanent charges. These lipids feature N‐methyl and secondary amine groups in their heads and three tails derived from epoxyalkanes, demonstrating superior pulmonary selectivity and efficiency. The proteomic study reveals that protein corona fingerprints may serve as a more comprehensive characteristic for facilitating the lung tropism of LNPs.

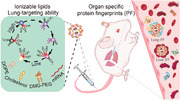

## 1 Introduction

Due to their remarkable success in the application of mRNA vaccines (BNT162b2 and mRNA‐1273) and Patisiran, lipid nanoparticles (LNPs) have emerged as the most advanced nonviral delivery vehicles for mRNA drugs.^[^ ^1^, ^2^, ^3^ ^]^ Classical LNPs have demonstrated high efficacy in delivering mRNA to the liver and muscles through intravenous and local injection. Nevertheless, extrahepatic delivery via systemic administration remains challenging^[^ ^4^ ^]^ and has been considered to be one of the major obstacles to the full exerting of mRNA drug capabilities in the treatment of pulmonary diseases.

Since the first reported SORT delivery system,^[^ ^5^ ^]^ cationic lipids have been believed to be vital for lung targeting and have been proven by many researchers.^[^ ^6^, ^7^, ^8^, ^9^, ^10^, ^11^ ^]^ Following the breakthrough in lung‐targeted delivery with the addition of cationic lipids, scientists also discover that some lipids and polymers with nonpermanent charges are efficient for lung‐targeted delivery, such as N‐serial LNPs with an amide bond in the tail,^[^ ^12^ ^]^ one‐component ionizable amphiphilic Janus dendrimers,^[^ ^13^, ^14^, ^15^ ^]^ siloxane‐incorporated lipids,^[^ ^16^ ^]^ charge‐alternating releasable transporter,^[^ ^17^ ^]^ poly(β‐amino esters),^[^ ^18^, ^19^, ^20^, ^21^ ^]^ amidine‐incorporated lipids,^[^ ^22^ ^]^ and so on.^[^ ^23^, ^24^ ^]^ These nonpermanently charged materials attract much attention due to their simpler formulation and potentially less immunogenic nature. Despite these advances, rational design of ionized lipids with high lung selectivity upon systematic administration and precise structure‐function relationships remains in urgent need. The synthesis of lung‐targeted lipids with systemic variation in chemical structures and the study of potential correlations between lipid structure, corona characteristics, and lung specificity of LNPs will provide guidance for the rational design of lung‐selective lipids.

Herein, we design a series of nonpermanently charged ionizable lipids with the adjustment of the structures of lipid heads and tails, as well as the number and length of the tails to address the above challenges. High lung‐selectivity was achieved with lipids possessing N‐methyl and secondary amine groups in the heads, as well as three‐tail skeletons originated from epoxyalkanes. The protein corona adsorbed on the surface of LNPs was analyzed with those containing lipids that showing minimum changes in chemical structure while demonstrating a gradual shift of organ tropism from the liver to lung. The proteomic analysis revealed a strong correlation between lung selectivity and the “protein fingerprints.” We found that arginine‐glycine‐aspartic acid (RGD)‐rich proteins in the protein corona might have a strong correlation with organ selective delivery of mRNA. RGD fragment usually acts as cell recognition site for adhesive proteins present in extracellular and blood matrices, such as fibronectin, vitronectin, and fibrinogen.^[^ ^25^ ^]^ These results will improve the design and screening efficiency of lung‐selective lipids, and facilitate the application of mRNA therapy in the treatment of pulmonary diseases delivered by LNPs.

## 2 Results and Discussion

### 2.1 Combinatorial Screening of Ionizable Lipids

**Figure 1:**
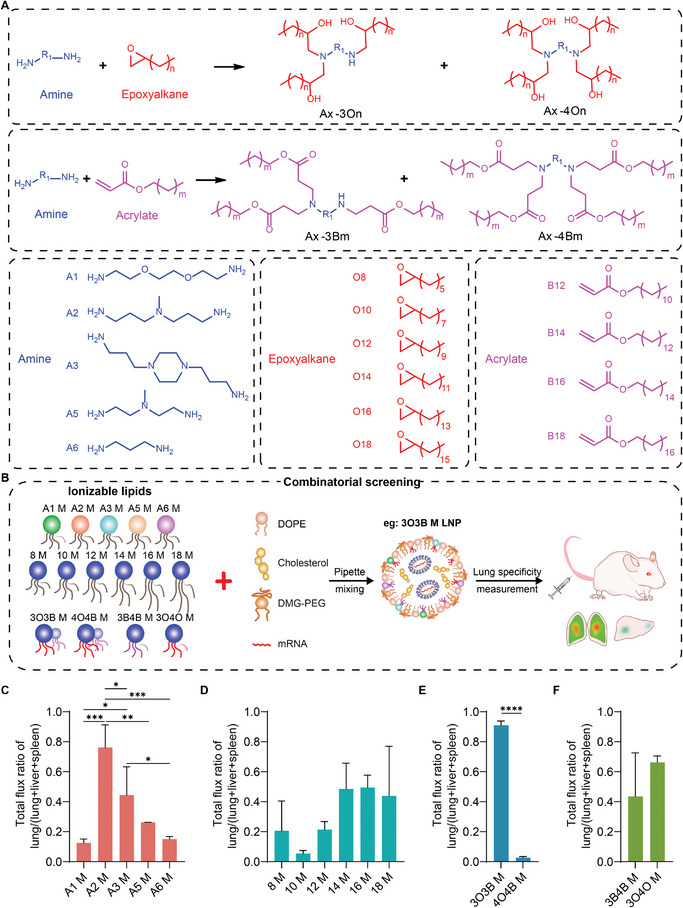
Combinatorial screening of ionizable lipids. A) General synthesis procedures for the ionizable lipid library. Structures of amines, epoxyalkanes, and acrylates used for constructing the library. B) Schematic illustration of a classification strategy for ionizable lipids for combinational screening. Ax M (A1–A6 m) represents the mixture of all lipids carrying the same head; X M (8–18 m) indicates that ionizable lipids with the same tail length were mixed together for screening; 3O3B M, 4O4B M, 3B4B M, and 3O4O m represent mixtures of ionizable lipids with three tails, four tails, tails derived from acrylates, and tails derived from epoxyalkanes, respectively. C–F) Calculation of the Luc mRNA (0.2 mg kg^−1^) expression ratio in the lung of each classified lipid mixture. Data are presented as mean ± s.d. *n* = 3. The statistical analysis of 1C,D was performed using one‐way ANOVA Turkey's multiple comparisons test, substantial differences between groups were indicated by ^*^ *p* < 0.05, ^**^ *p* < 0.01, ^***^ *p* < 0.001, and ^****^ *p* < 0.0001. The none significant difference between groups was not shown in Figure 1C,D. The statistical analysis of 1E,F was performed using an unpaired *t*‐test, substantial differences between groups were indicated by ^*^ *p* < 0.05, ^**^ *p* < 0.01, ^***^ *p* < 0.001, and ^****^ *p* < 0.0001. The none significant difference between groups was not shown in Figure 1F.

We selected five different structures of diamines with one primary amine group (‐NH_2_) on each side as head groups, providing four reaction sites for nucleophilic addition reactions. Epoxyalkanes or acrylates with varying lengths of alkyl chains were utilized as acceptors for nucleophilic addition reactions (**Figure** **1**A). The epoxyalkanes reacted with amine heads through a ring‐opening reaction. The acrylates reacted with amine heads via the Michael addition reaction. The lipids were obtained after the separation with flash column chromatography and were named as Ax‐yOn or Ax‐yBm, where Ax, y, On, and Bm represent the head of the lipid, the number of tails, the tails derived from epoxyalkanes, and the tails originating from acrylates, respectively. It was reported that there is a weak correlation between the in vitro and in vivo nucleic acid delivery mediated by nanoparticles^[^ ^16^, ^26^ ^]^ and the organ selectivity cannot be evaluated from in vitro experiments. Therefore, in vivo and ex vivo screenings were carried out for these lipids directly. A mixture of ionizable lipids (M) was used for the preliminary fast screening. We grouped these lipids based on the head structures (five groups: A1 M, A2 M, A3 M, A5 M, and A6 m), tails lengths (six groups: 8 M, 10 M, 12 M, 14 M, 16 M, and 18 m), tail numbers (two groups: 3O3B M and 4O4B m), and tail structures (two groups: 3B4B M and 3O4O m). LNPs encapsulating firefly luciferase mRNA (Luc mRNA) were prepared according to the classical formulation^[^ ^27^ ^]^ and generated through rapid pipet mixing of the aqueous phase and ethanol solution. Then the bioluminescence following the administration of luciferin was measured to evaluate the expression efficiency (Figure 1B).

The lung targeting ability was evaluated by calculating the ratio of mRNA expression in the lungs, i.e., “total flux of lung/(total flux of lung + total flux of liver + total flux of spleen).” The lung‐selectivity of LNPs formulated with different heads indicated a substantial impact of head structures on the organ selectivity, with A2 stood out as the optimal head for pulmonary delivery (Figure 1C). An increase in the lung ratio was observed as the tail length reached 14, with the highest average value appearing at 16 (Figure 1D). Unexpectedly, a substantial difference in organ targeting ability was found between the three‐tail mixture 3O3B m and the four‐tail mixture 4O4B m. The ratio of mRNA expression in the lung of 3O3B m was over 0.9, while that of 4B4O m was ≈ 0, indicating a completely different organ‐targeting ability (Figure 1E). This highlighted the potentially important role of tail numbers on organ specificity. The tail structures also played an influential role in organ selectivity when comparing the ratio of lung for 3B4B M and 3O4O m, although not as distinct as that of tail numbers (Figure 1F). Based on these results, we moved forward to explore the lung‐targeting ability of individual ionizable lipids in detail. This exploration will provide insight into the structure‐function relationship.

### 2.2 Exploration of the Structure‐Function Relationship of Lung‐Targeted Ionizable Lipids

**Figure 2:**
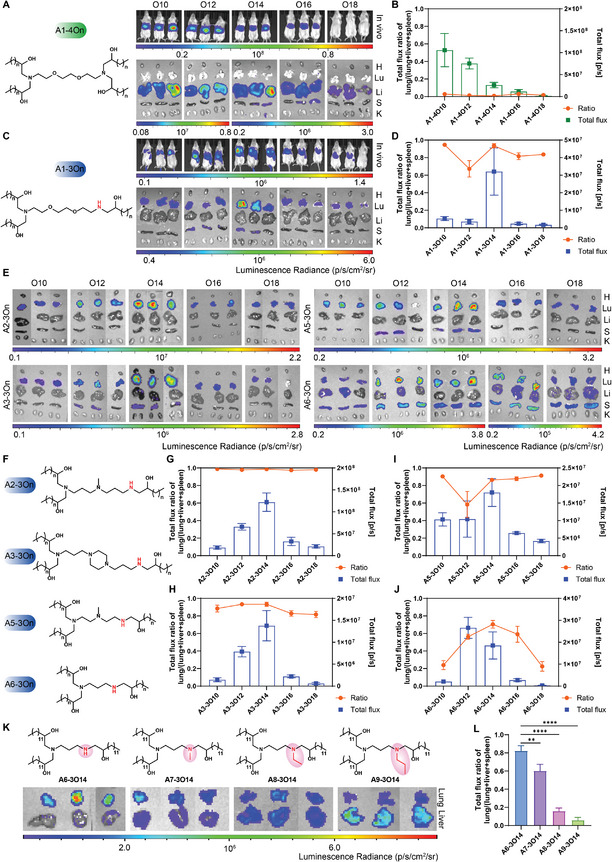
Assessment of the delivery efficiency and selectivity of LNPs. A) In/Ex vivo bioluminescent imaging of mice treated with A1‐4On LNPs. B) The total flux in the livers of 2A and lung‐selectivity of A1‐4On LNPs. C) In/Ex vivo bioluminescent imaging of mice treated with A1‐3On LNPs. D) The total flux in the lungs of 2C and lung‐selectivity of A1‐3On LNPs. E) Ex vivo evaluation of the expression efficiency of Luc mRNA in A2‐3On LNPs, A3‐3On LNPs, A5‐3On LNPs, and A6‐3On LNPs. The major organs were displayed in the order of heart (H), lung (Lu), liver (Li), spleen (S), and kidney (K) from top to bottom in Figure 2A,C,E. F) The general structures of A2‐3On, A3‐3On, A5‐3On, and A6‐3On. G–J) The total flux in the lungs of E and lung selectivity of A2‐3On LNPs, A3‐3On LNPs, A5‐3On LNPs, and A6‐3On LNPs. K) Examine the essential role of a secondary amine group for lung‐targeting delivery by adding different lengths of alkyls to the secondary amine group. The organs were displayed in the order of lung and liver from top to bottom in Figure 2K. L) The ratio of mRNA expression in the lung. Lung selectivity decreased as the blocking group adding to the ‐NH group in the head of A6‐3O14 shifted from ‐CH_3_, ‐CH_2_CH_3_ to ‐CH_2_CH_2_CH_3_. The statistical analysis was performed using one‐way ANOVA with Dunnett's multiple comparisons test, substantial differences between groups were indicated by ^*^ *p* < 0.05, ^**^ *p* < 0.01, ^***^ *p* < 0.001, and ^****^ *p* < 0.0001. All the groups were compared with A6‐3O14 in 2 K. In this figure, all the mice were administrated with the Luc mRNA‐loaded LNPs at a dose of 0.2 mg kg^−1^ through the tail vein. In/Ex vivo images were taken at 6 h post injection (*n* = 3). Lung‐selectivity was calculated by a total flux of lung/(total flux of lung + total flux of liver + total flux of spleen). Data are presented as mean ± s.d.

We started the validation of the structure‐function relationship by examining the effect of tail number, which might be the most influential factor on lung specificity. The expression of the Luc mRNA delivered by A1‐4On lipids with four tails was primarily found in the liver and only a feeble signal was observed in the corresponding spleen (**Figure** **2**A,B). In contrast, the expression of Luc mRNA delivered by A1‐3On LNPs with three tails was predominately detected in the lungs (Figure 2C,D). The expression efficiency of the three‐tail lipids A1‐3On reached a peak value at **A1‐3O14**.

To confirm the function of the three‐tail skeleton in the lipids, we maintained this vital feature and further evaluated the three‐tail lipids with heads A2, A3, A5, and A6. We discovered that all four series of lipids displayed excellent to fairly good mRNA expression predominantly in the lungs (Figure 2E–J). In contrast to the above results, pulmonary mRNA expression delivered by the four‐tailed skeleton lipids (A2‐4On, A3‐4On, A5‐4On, and A6‐4On) was greatly reduced (Figure S1, Supporting Information), with the exception of **A2‐4O18**. The four‐tail A2‐4O18 exhibited comparable lung‐selectivity to the A2‐3On series, which seems to indicate that longer tail‐length is beneficial for lung‐targeting delivery among the four‐tail A2‐4On LNPs. This is intriguing and worth further investigation in future work. These results validated that the number of tails affected the organ tropism. The top performance was shown in A2‐3On group, as the ratio of the lung for these lipids was all close to 1 (Figure 2E–G). This is consistent with the conclusion drawn from combinatorial screening, which indicated that the mixture carrying A2 heads displayed the highest lung selectivity. Moreover, lipids carrying three‐tail with 14 carbons (i.e., Ax‐3O14) demonstrated the highest mRNA expression efficacy in the lung in all groups (Figure 2G–J), except that the peak expression efficiency for A6‐3On group was found with **A6‐3O12**.

We realized that the structural variation between three‐tail and four‐tail lipids lies in the presence of a secondary amine (‐NH‐) group in the head of the three‐tail lipids, which might play a critical role in lung‐selective delivery together with the three‐tail skeleton. Then ionizable lipids with NH groups blocked by alkyl groups were synthesized to verify the essential role of a secondary amine in the head for lung targeting delivery. **A6‐3O14** was selected as a proxy compound, and then a methyl, ethyl, or a propyl group was added to the NH group in the head of A6 to yield **A7‐3O14**, **A8‐3O14**, and **A9‐3O14**, respectively. The lung‐targeting ability gradually declined as the alkyl group shifted from methyl to ethyl and propyl (Figure 2K,L), indicating the crucial role of NH in lung‐selective delivery. Recently, it has been reported that the presence of multiple secondary amine groups in two‐tail lipids could facilitate efficient mRNA delivery to spleen T cells.^[^ ^28^ ^]^

**Figure 3:**
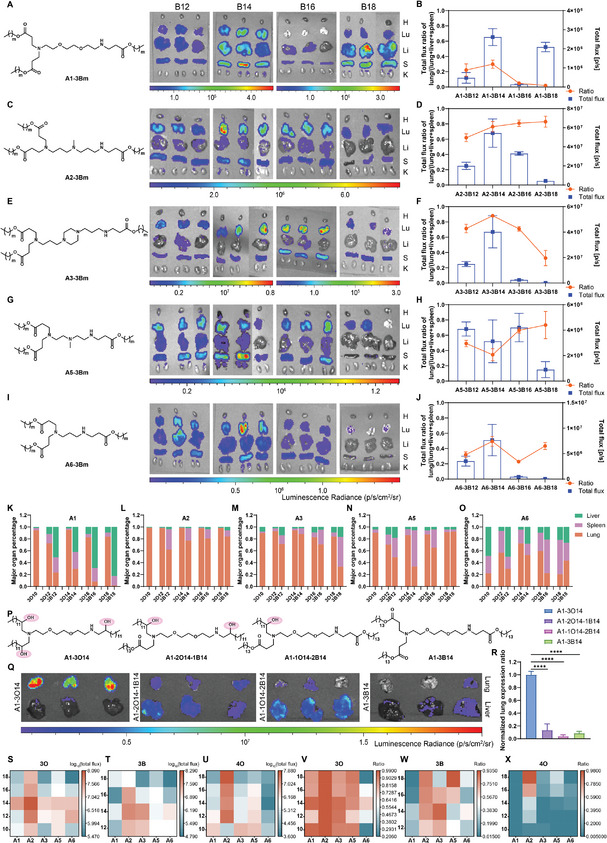
Evaluation of the impact of lipid tail structures on delivery efficiency and selectivity. A,C,E,G,I) Evaluation of the expression efficiency of Luc mRNA encapsulated by A1‐3Bm LNPs, A2‐3Bm LNPs, A3‐3Bm LNPs, A5‐3Bm LNPs, and A6‐3Bm LNPs, respectively. The major organs were displayed in the order of heart (H), lung (Lu), liver (Li), spleen (S), and kidney (K) from top to bottom in Figure 3A,C,E,G,I. B,D,F,H,J) Calculation of the ratio of mRNA expression in the lung by total flux of lung/(total flux of lung + total flux of liver + total flux of spleen) according to the ex vivo results from (A,C,E,G,I), respectively. K–O) Evaluation of the effect of tail structures on the organ‐selectivity by calculating the percentage of the total flux of Luc mRNA expression in the lung, liver, and spleen delivered by different three‐tail lipids. P) The structures of **A1‐3O14**, **A1‐2O14‐1B14**, **A1‐1O14‐2B14,** and **A1‐3B14**. Q) Evaluation of the critical role of three tails derived from epoxyalkanes in the ionizable lipids for the high‐performance lung‐specific delivery of mRNA. The incorporation of acrylates into tails led to shifting of mRNA expression from lung to liver. R) Calculation of the total flux ratio in the lung by total flux of lung/(total flux of lung + total flux of liver + total flux of spleen). The statistical analysis was performed using one‐way ANOVA with Dunnett's multiple comparisons test, substantial differences between groups were indicated by ^*^ *p* < 0.05, ^**^ *p* < 0.01, ^***^ *p* < 0.001, and ^****^ *p* < 0.0001. All the groups were compared with A1‐3O14. S–U) Heatmap of mRNA expression efficiency in the lung delivered by Ax‐3On LNPs, Ax‐3Bm LNPs and Ax‐4On LNPs. V–X) Heatmap of mRNA expression ratio (total flux of lung/(total flux of lung + total flux of liver + total flux of spleen)) in the lung delivered by Ax‐3On LNPs, Ax‐3Bm LNPs, and Ax‐4On LNPs. In this figure, all the mice were administrated with the Luc mRNA‐loaded LNPs at a dose of 0.2 mg kg^−1^ through the tail vein. Ex vivo images were taken at 6 h post‐injection (*n* = 3). Data are presented as mean ± s.d.

Subsequently, five amine heads carrying three‐tail lipids (Ax‐3Bm) derived from acrylates were selected to assess the impact of tail types on organ selectivity. Ex vivo imaging showed mRNA expression in multi‐organs including lung, liver, and spleen, regardless of head structures or tail lengths (**Figure** **3**A–J). The quantification of the mRNA expression ratio in these organs further suggested that Ax‐3Bm demonstrated lower lung selectivity than Ax‐3On, implying the vital role of hydroxy containing tails (Figure 3K–O). To further validate this hypothesis, **A1‐3O14** was selected as a proxy, and tails derived from epoxyalkanes were substituted with acrylates to generate **A1‐2O14‐1B14** and **A1‐1O14‐2B14**. It was found that the replacement of the tails of **A1‐3O14** with acrylate resulted in a significant dropping in lung selectivity, indicating that the simultaneous existence of three hydroxy groups in the tails was crucial for highly efficient lung‐targeted delivery (Figure 3P–R).

The heatmap of lung radiance (Figure 3S–U) and the mRNA expression ratio in the lung (Figure 3V–X) for Ax‐3On, Ax‐3Bm, and Ax‐4On suggested that lipids with A2 head (containing N‐methyl groups) showing better selectivity and efficacy than the others in each series. Therefore, lipids characterized by containing NH and N‐methyl groups in the heads, carrying hydroxyl groups in the tails, as well as three‐tail skeleton are likely to demonstrate high lung‐selectivity. All ionizable lipids involved were characterized by ^1^H NMR and MALDI‐TOF MS (Figures S2–S153, Supporting Information). Overall, the lipid **A2‐3O14** demonstrated optimal delivery efficiency and selectivity.

Finally, to validate the reproducibility of lung‐targeting properties, we adopted another three classical formulations (Table S1, Supporting Information) commonly used for SM‐102/MC3, C12‐200, and ALC‐0315 to prepare A2‐3O14 LNPs. As it is shown in Figure S154, Supporting Information, the high lung‐selective property was maintained regardless of the changes of formulation. All of the groups demonstrated high lung‐targeting ability.

### 2.3 Analysis of Potential Proteins That Mediated Organ‐Targeting Ability

According to the structure‐function relationship discussed above, some representative lipids with lung‐targeting ability, ranging from high to low (liver‐targeted) were selected for the proteomic assay. To directly compare the selectivity of lung and liver, the targeting ability was evaluated using the lung‐targeting index TAR, calculated by the equation “TAR = (total flux of lung – total flux of liver)/(total flux of lung + total flux of liver).” The value ranges from 1 to −1, indicating the shift of organ selectivity from lung to liver. **A2‐3O14**, **A2‐3B14**, **A2‐4O14**, **A1‐3O14**, **A1‐3B14**, and **A1‐4O14** represent the presence of NH+NCH_3_+OH, NH+NCH_3_, NCH_3_+OH, NH+OH, NH, and OH groups in the lipids, respectively. **A6‐3O14**, **A7‐3O14**, **A8‐3O14**, and **A9‐3O14** were selected to demonstrate the gradual shift of mRNA delivery from the lung to liver with the minimum variation in chemical structures. LNPs were incubated with mouse plasma, followed by extraction of the protein corona using a standard procedure. Label‐free quantitative technique (LFQ) was employed to identify and quantify the content of protein corona.^[^ ^29^, ^30^, ^31^ ^]^

**Figure 4:**
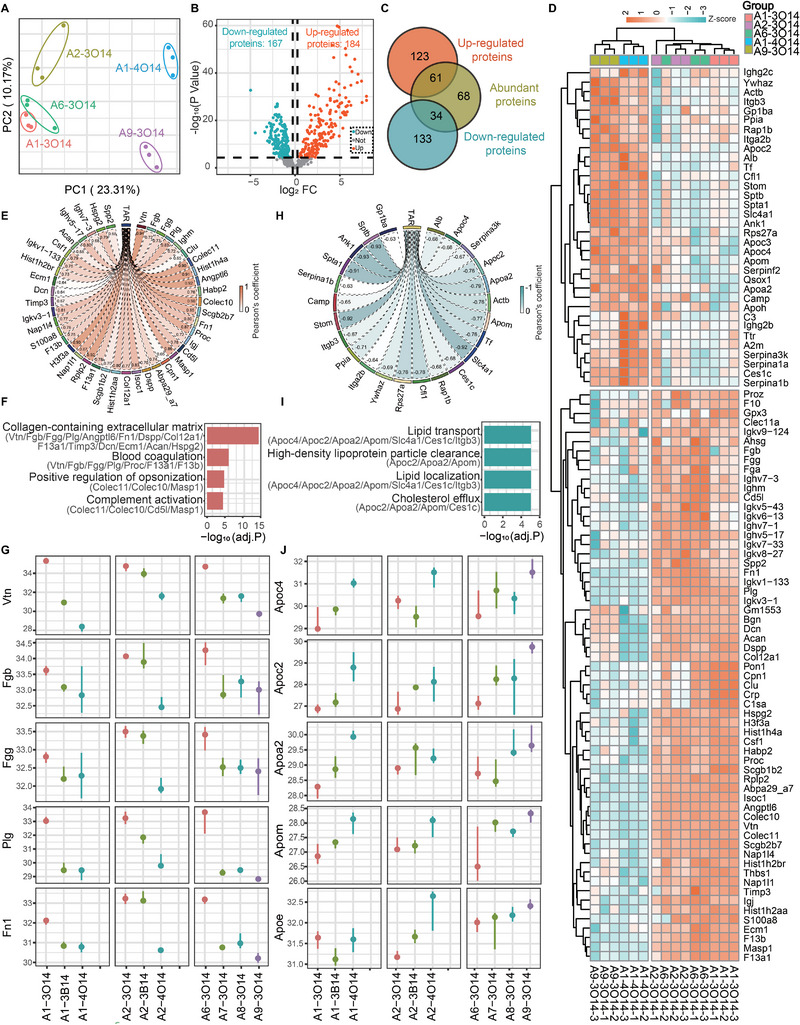
Exploration of the correlation between the proteins in the protein corona and organ selectivity. A) Principal component analysis (PCA) of proteins pulled down by 3 lung‐targeting LNPs (**A1‐3O14**, **A2‐3O14** and **A6‐3O14**) and 2 liver‐targeting LNPs (**A1‐4O14** and **A9‐3O14**). B) Volcano plot of all proteins. Proteins enriched in lung or liver targeting LNPs with a *p*‐value less than 0.05 and a fold change (FC) value greater than 1.2 were marked as up‐regulated or down‐regulated, respectively. C) Venn diagram of different proteins against abundant proteins (abundance> 0.1%). D) Heatmap of significantly regulated abundant proteins shown in the Venn diagram. E) Chord diagram of TAR (calculated from the ten selected LNPs) and significantly regulated abundant proteins (positive correlation). A p‐value from Pearson's test less than 0.05 was defined as a correlated protein. F) The biological functions of proteins listed in E were annotated by gene ontology (GO) enrichment. G) The abundance (log_2_ intensity) of the up‐regulated proteins (lung tropism) in the protein corona of the ten selected LNPs with varying degrees of lung‐targeting ability (*n* = 3). H) Chord diagram of TAR and significantly regulated abundant proteins (negative correlation). A *p*‐value from Pearson's test less than 0.05 was defined as a correlated protein. I) The biological functions of proteins displayed in H were annotated by GO enrichment. J) The abundance (log_2_ intensity) of the down‐regulated proteins (liver tropism) in the protein corona of the ten selected LNPs with varying degrees of lung‐targeting ability (*n* = 3).

Even though protein corona has been proposed to be vital for organ‐selective delivery, the correlation between protein corona and pulmonary delivery is still ambiguous. We first selected the typical examples to distinguish the most significant disparity between lung‐ and liver‐targeting LNPs. As shown in the principal component analysis (PCA) diagram (**Figure** **4**A), there was a distinct separation between the data for lung‐targeting LNPs (**A1‐3O14**, **A2‐3O14,** and **A6‐3O14**) and liver‐targeting LNP (**A1‐4O14** and **A9‐3O14**), which reflected the good quality of the data. Herein, the amount of protein absorbed by the two liver‐targeting LNPs was set as the baseline for the analysis. Proteins exhibiting a fold change (FC) > 1.2 and a *p* < 0.05 were marked as either up‐regulated (lung tropism) or down‐regulated ones (liver tropism). The statistical analysis of the proteomic data for the five representative LNPs indicated that 184 proteins were significantly up‐regulated and 167 proteins were significantly down‐regulated (Figure 4B). Following these results, proteins with abundances higher than 0.1% were selected to analyze the relationship between the abundance of these proteins and in vivo lung‐targeting property. The Venn diagram (Figure 4C) showed that 61 abundant proteins were up‐regulated and 34 abundant proteins were down‐regulated. Clustering analysis of these 95 proteins automatically classified the lung‐targeted and liver‐targeted LNPs into two main clusters (Figure 4D). The heat map of the cluster on the left represented the liver‐targeted group, while the one on the right was a lung‐targeted group. There was a significant difference in the abundance of the protein enriched in these two groups, and the potential proteins that facilitate lung‐targeted delivery can be identified.

Forty‐three up‐regulated proteins that significantly correlated with TAR (*p* < 0.05) were identified from the abundant proteins displayed in the Venn diagram, and demonstrated through a chordal graph (Figure 4E). Gene ontology (GO) enrichment analysis for proteins that are positively and negatively correlated with the target variable was conducted to identify the potential enriched pathways of the proteins involved in lung‐targeting delivery. The analysis revealed that the collagen‐containing extracellular matrix, blood coagulation, positive regulation of opsonization, and complement activation pathway were prominent (Figure 4F). It has been proposed that vitronectin was enriched in the protein corona of LNPs and then bound to endothelia cells with integrin αvβ3 receptor expression,^[^ ^32^, ^33^, ^34^ ^]^ leading to lung tropism. In addition to vitronectin (Vtn), the pulmonary targeting LNPs were also found to be in favor of adsorbing other RGD‐rich proteins, such as fibrinogen (Fgb/Fgg), fibronectin (Fn1), and dentin‐saliva‐phosphoprotein proprotein (Dspp). These proteins were shown to be in top correlation with the collagen‐containing extracellular matrix (Figure 4F,G). The Brenner group proposed that lung‐targeted LNP containing the positively charged DOTAP would adsorb negatively charged fibrinogen, implying the potential risk of thrombus formation.^[^ ^35^ ^]^ We also discovered that lung‐targeting LNPs could activate the coagulation pathway by adsorbing proteins such as fibrinogen, coagulation factor XIII (F13a1/F13b), and core proteoglycan (Dcn), posing a potential risk of thrombosis (Figure 4F,G; Figure S155, Supporting Information). The nonpermanently charged LNPs discovered here would also recruit fibrinolytic and anticoagulant proteins, including plasminogen (Plg), and protein C (the inactivator of coagulation factors Va and VIIIa) (Figure 4F,G; Figure S155, Supporting Information). According to the Brenner group's results, the application of clot prevention techniques such as the pre‐treatment of bivalirudin and decreasing the size of nanoparticles would prevent LNP‐induced clotting.^[^ ^35^ ^]^ The other representative proteins correlated with lung selectivity were also listed in Figure S155 (Supporting Information). The function of these proteins needs to be further confirmed. Further work will aim to validate the function of these high‐abundance proteins in mediating lung selective delivery through the corresponding gene knockout mouse models.

Furthermore, 24 down‐regulated proteins that significantly correlated with TAR (*p* < 0.05) were also identified (Figure 4H). The liver‐targeting LNPs primarily absorb proteins that are involved in lipid metabolism pathways, such as lipid transport, high‐density lipoprotein particle clearance, lipid localization, and cholesterol efflux (Figure 4I). The liver tropism was identified to be significantly associated with Apoc4, Apoc2, Apoa2, and Apom (Figure 4J). Apoe was also listed as a typical protein dominating liver tropism, although the FC of abundance was not significant according to our analysis. Apolipoproteins were enriched in the pathways regulating liver‐targeted delivery, which is consistent with previously discovered other liver‐targeting delivery systems.^[^ ^36^, ^37^, ^38^ ^]^ The dominant types of apolipoproteins appear to vary with the structure of ionizable lipids and the individual environment. Recently, the AstraZeneca team found that the hepatocyte targeting capability of Dlin‐MC3‐DMA LNPs may rely on Apoc4, Apoc2, Apoa2, and ApoB located in high‐density lipoproteins, very‐low‐density lipoproteins, and chylomicron lipoproteins in obese rats, instead of the well‐known Apoe.^[^ ^39^ ^]^ We discovered that Apoc4, Apoc2, Apoa2, and Apom were all associated with hepatic‐targeted delivery. The other proteins correlated with liver selectivity were also listed in Figure S156 (Supporting Information).

The physicochemical properties including LogP, LogD, Z‐average, pKa, and the morphology of representative LNPs were also characterized (Table S2 and Figures S157 and S158, Supporting Information). No significant dependency was found between the TAR and physicochemical properties, including LogP, LogD, Z‐average, and pKa. The zeta potentials in PBS and H_2_O exhibited a moderate to strong correlation, with R values of 0.66, and 0.76, respectively.

### 2.4 The Biodistribution and Biosafety of LNPs

**Figure 5:**
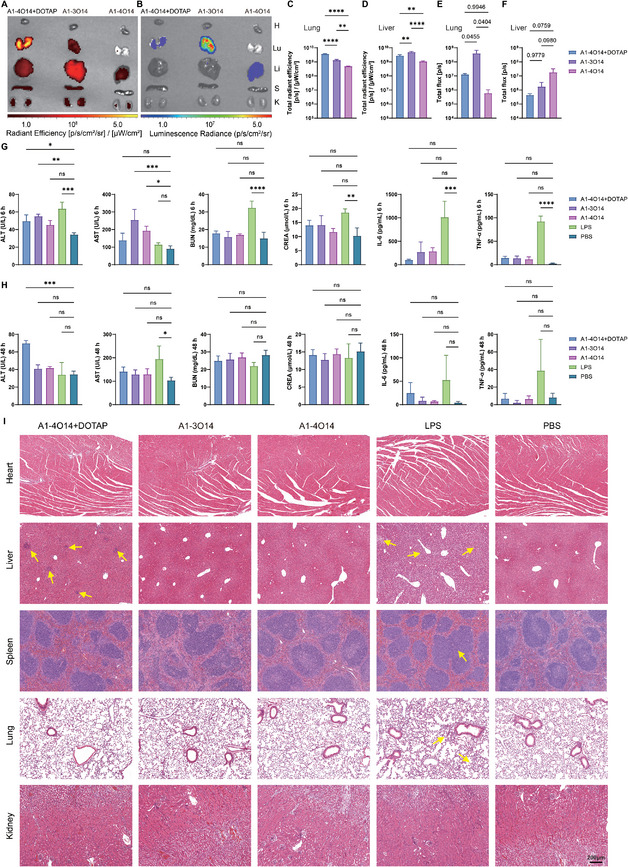
The biodistribution and biosafety of **A1‐3O14** LNPs. A) The accumulation of lung specific **A1‐4O14**+DOTAP LNPs, lung‐specific **A1‐3O14** LNPs, and liver‐specific **A1‐4O14** LNPs in the major organs was indicated by the intensity of Cy5 fluorescence. PBS was used as a reference group. B) The Cy5‐luc‐mRNA expression (0.5 mg kg^−1^) in the major organs was delivered by **A1‐4O14**+DOTAP LNPs, **A1‐3O14** LNPs, and **A1‐4O14** LNPs. C) Total radiance efficiency of Cy5‐fluorescence in the lungs from A. D) Total radiance efficiency of Cy5‐fluorescence in the livers from A. E) Total flux of Cy5‐luc‐mRNA expression in the lungs from B. F) Total flux of Cy5‐luc‐mRNA expression in the livers from B. Ex vivo images were taken 6 h post injection (*n* = 3). The major organs were displayed in the order of heart (H), lung (Lu), liver (Li), spleen (S), and kidney (K) from top to bottom in Figure 5A,B. G) The toxicity of **A1‐4O14**+DOTAP LNPs, **A1‐3O14** LNPs and **A1‐4O14** LNPs (0.8 mg kg^−1^) were measured at 6 h and H) at 48 h through the assessment of the liver function (alanine aminotransferase (ALT), and aspartate aminotransferase (AST)), kidney function (blood urea nitrogen (BUN), creatinine (CREA)), and inflammatory cytokines (IL‐6 and TNF‐α). LPS (5 mg kg^−1^) and PBS groups were administrated as a positive and a negative control, respectively. *n* = 3. C‐H, data are presented as mean ± s.d. The statistical analysis was performed using one‐way ANOVA with Dunnett's multiple comparisons test, substantial differences between groups were indicated by n.s. = not significant, ^*^ *p* < 0.05, ^**^ *p* < 0.01, ^***^ *p* < 0.001, and ^****^ *p* < 0.0001. I) Tissue sections of major organs were prepared for H&E staining 48 h post administration. Scale bar, 200 µm.

The accumulation of LNPs in organs was assessed. Cy5‐luc‐mRNA was encapsulated with lung‐targeting **A1‐3O14** LNPs. DOTAP and **A1‐4O14** were used to formulate lung‐selective positive control (**A1‐4O14**+DOTAP LNPs). The accumulation of LNPs was evaluated by measuring the fluorescence intensity of Cy5. As it was indicated by the fluorescence imaging, the accumulation in the lung for **A1‐4O14**+DOTAP LNPs surpassed that of **A1‐3O14** LNPs (**Figure** **5**A,C). While the expression efficacy of Cy5‐luc‐mRNA (indicated by the total flux of the bioluminescence) in the lung for **A1‐3O14** LNPs was higher than of **A1‐4O14**+DOTAP LNPs, indicating that a lower amount of accumulation resulted in higher mRNA expression efficacy in the lung for **A1‐3O14** LNPs (Figure 5B,E). Even though a larger amount of accumulation was found for **A1‐3O14** LNPs in the liver, the mRNA expression efficacy in the liver was still significantly lower than that in the corresponding lung (Figure 5D–F). The correlation between mRNA translation and LNP accumulation is usually nonlinear, which is affected by many factors such as the type of ionizable lipids and the route of administration.^[^ ^21^, ^40^ ^]^ Furthermore, as it is indicated from recent research,^[^ ^10^ ^]^ the presence of cholesterol was the main reason for high liver accumulation. Therefore, it is worth try to optimize the formulation and remove cholesterol from it to reduce the hepatic accumulation in future research.

Finally, the biosafety assessment of the LNPs was conducted. At 6 h, A1‐4O14+DOTAP, A1‐3O14, A1‐4O14 LNPs, and LPS treated mice exhibited liver injury with elevated levels of ALT and/or AST compared to the PBS control group. Mice treated with **A1‐3O14** and **A1‐4O14** LNPs showed recovery to normal levels at 48 h (Figure 5G,H). The positive control group treated with LPS also demonstrated significantly increased levels of inflammatory cytokines (IL‐6 and TNF‐*α*) as well as severe kidney injury (BUN and CREA) at 6 h. H&E staining of major organs obtained at 48 h post‐administration revealed severe lung, liver, and spleen injuries in the LPS‐injected mice group indicated by yellow arrows (Figure 5I). Liver injury (represented by yellow arrows) was observed in the **A1‐4O14**+DOTAP LNPs group. No apparent injuries were observed in the organ slices from the **A1‐3O14**, **A1‐4O14**, or PBS groups.

## 3 Conclusion 

In summary, we have designed and synthesized an ionizable lipid library that has a defined structure‐function relationship for efficient delivery of mRNA to lungs following I.V. administration. The proteomic analysis reveals a potential correlation between the RGD‐rich proteins (includes vitronectin, fibrinogen, fibronectin, and dentin‐saliva‐phosphoprotein proprotein) in the protein corona and lung‐homing capacity of LNPs. The findings in this work deepen the understanding of the correlation between the chemical structure of ionizable lipids, the protein corona, and the lung‐selective delivery ability of LNPs, which will facilitate the extension of mRNA therapy to pulmonary diseases.

## Conflict of Interest

The authors declare no conflict of interest.

## Ethic Approval Statement

All animal experiments were approved by the Institutional Animal Care and Use Committee of ShanghaiTech University (Approval of Animal Ethical and Welfare Number: 20230527001) and were consistent with the governmental regulations of China for the care and use of animals.

## Supporting information



Supporting Information

Raw data for proteomics

## Data Availability

The data that support the findings of this study are available from the corresponding author upon reasonable request.
