# Supplementary material for: Lung‐Specific mRNA Delivery by Ionizable Lipids with Defined Structure‐Function Relationship and Unique Protein Corona Feature
Source: Adv Sci (Weinh). 2025 Feb 18;12(14):2416525. doi: 10.1002/advs.202416525 (PMC11984862; doi:10.1002/advs.202416525)
Supplement: Supplementary file 1 — Supporting Information [file ADVS-12-2416525-s001.docx]

**Lung-specific mRNA Delivery by Ionizable Lipids with Defined Structure-function Relationship and Unique Protein Corona Feature**

Xiaoyan He,**^+^** Runyuan Wang,**^+^** Yan Cao,**^+^** Yan Ding, Yan Chang, Haoru Dong, Rong Xie, Guisheng Zhong, Huiying Yang, and Jianfeng Li^*^

Table of contents

1. Experimental section 2

2. Supplementary figures S17

3. Characterization of ionizable lipids figure S28

4. Supplementary table S185

5. Supplementary figures S15485

6. References91

**Experimental section**

**Materials**

Reagents and solvents were obtained from commercial sources. Amines, epoxides and acrylates were obtained from Bidepharm, Aladdin, Ding chem, Adamas or leyan. Unless otherwise noted, all synthesis reagents were purchased from commercial suppliers and used without further purification. All the glassware was dried in oven before usage. Crude products were purified through flash chromatography (Buchi). Mass spectra were measured with an Autoflex Speed MALDI-TOF mass spectrometer (Bruker). 2,5-dihydroxybenzoic acid was applied as matrix for the measurements. ^1^H-NMR spectra were performed on 500 MHz NMR spectrometers (Bruker), using the signal of the deuterated solvent as the internal standard. The chemical shifts were reported in part per million (ppm).

1,2-Dioleoyl-sn-glycero-3-phosphoethanolamine (DOPE) and DSPC (1,2-distearoyl-sn-glycero-3-phosphocholine) were purchased from Avanti Polar Lipids (Birmingham, AL). Cholesterol was purchased from Adamas-beta (Shanghai, China). Polyethylene glycol [PEG] 2000 dimyristoyl glycerol [DMG] (DMG-PEG) was purchased from MACKLIN (Shanghai, China). Firefly Luciferase mRNA (N1-Me-Pseudo UTP) and Firefly Luciferase mRNA were purchased from novoproteins (Suzhou, China). Cy5 Firefly Luciferase mRNA (5-moUTP) was purchased from APExBIO. Dialysis membranes (WMCO, 3.5 kDa) were purchased from Shanghai Yuanye Bio-Technology Co., Ltd. D-luciferin was purchased from Admas.

**General procedures for synthesizing ionizable lipids**

Amine and 1,2-epoxyalkane or acrylate were added to pressure-proof tube in a molar ratio of 1:3.5 to obtain both three- and four-tail lipids products. The mixture was sealed in the tube and heated to 120 ℃ under a solvent-free condition. The mixture was stirred until the starting materials, amine, was completely consumed, and then cooled to room temperature. Crude products were purified by flash chromatography (silica gel), eluting with gradually increased solvent polarity from petroleum ether/ethyl acetate, and to dichloromethane/methanol, until all the products were collected. A1-4On, A2-4On, A3-4On, A5-4On, A6-4On, A7-3On, A8-3On, A9-3On, Ax-3Bm and Ax-4Bm were eluted out with petroleum ether/ethyl acetate (the ratio shifted from 100/0 to 0/100) and the more polar product A1-3On, A2-3On, A3-3On, A5-3On, and A6-3On were eluted out by DCM/MeOH (the ratio shifted from 95/5 to 75/25). The products were obtained as pale yellow to white waxy solids or viscous oil. The products were confirmed by MALDI-TOF mass spectra and ^1^H NMR.

**Synthesis of** **A2-3O14 and A2-4O14**

N,N-Bis(3-aminopropyl)methylamine (200 mg, 1.38 mmol) and 1, 2-epoxytetradecane (1.02 g, 4.83 mmol), were mixed in a sealed, pressure-proof tube. The mixture was heated to 120 ℃ and stirred until the starting material N,N-Bis(3-aminopropyl)methylamine was completely consumed. Then the mixture was cooled and purified by flash chromatography (silica gel), eluting with gradually increased solvent polarity from petroleum ether/ ethyl acetate (100/0 to 0/100), and to DCM/MeOH (100/0 to 75/25), until all the products were collected. The products were obtained as waxy solids. **A2-3O14** and **A2-4O14** were separated with yields of 15.9% and 29.9% (calculated by referring to the starting material N,N-Bis(3-aminopropyl)methylamine), respectively.

**Synthesis of A1-2O14-1B14**

1,8-Diamino-3,6-dioxaoctane (230 mg, 1.55 mmol) and 1, 2-epoxytetradecane (656 mg, 3.2 mmol) were mixed and stirred at 100 ℃ until the starting material was consumed. Then tetradecyl acrylate (417 mg, 1.55 mmol) was added and the mixture was further stirred for 12 hours. The crude products were purified by column chromatography (silica gel) eluting with DCM/MeOH (100/0 to 90/10). The product (189 mg) was obtained as a beige oil with a yield of 14.4%.

**Synthesis of A1-1O14-2B14**

1,8-Diamino-3,6-dioxaoctane (230 mg, 1.55 mmol) and tetradecyl acrylate (833 mg, 3.1 mmol) were mixed and stirred in room temperature until the starting material was consumed. Then 1, 2-epoxytetradecane (328 mg, 1.55 mmol) was added and the reaction temperature was increased to 100 ℃. The mixture was stirred for another 12 hours. The crude products were purified by column chromatography (silica gel) eluting with DCM/MeOH (100/0 to 90/10). The product (213 mg) was obtained as a beige oil with a yield of 12.4%.

**Combinatorial screening of ionizable lipids**

The ionizable lipids were classified according to the structures of heads (five groups, A1M, A2 M, A3 M, A5 M, and A6 M), the structures of tails (two groups, 3O4O M, and 3B4BM), the length of tails (six groups, 8 M, 10 M, 12 M, 14 M, 16 M, 18 M) and the number of tails (two groups, 3O3B M, and 4O4B M). For example, A1 M represented the mixture of all ionizable lipids containing A1 heads. A2 M, A3 M, A5 M and A6 M were similar to A1 M with the changes of the lipid heads. 3O3B M and 4O4B M represented the mixture of ionizable lipids carrying three-tail and four-tail, respectively. 8 M and 10 M represented the mixture of all ionizable lipids formed from O8 and O10 with different heads, respectively. 12 M represented the mixture of all lipids formed from O12 and B12 with different heads. 14 M, 16 M and 18 M were similar to 12 M with the variation of the chain length of the epoxyalkanes and acrylates. 3B4B M and 3O4O M were the mixture of all lipids formed from different heads with acrylates or epoxyalkanes, respectively.

**LNPs formation and characterization**

The lipid mixture containing synthetic ionizable lipids, DOPE, cholesterol, and DMG-PEG2000 was prepared in ethanol at 50%: 10%: 38.5%: 1.5% (unless otherwise stated), and the mRNA was diluted in sodium citrate buffer (10 mM, pH = 3.0). The weight ratio of total lipids to mRNA was 20. The two phases were rapidly mixed by pipette with a volumetric ratio of ethanol: aqueous = 1:3, then it was dialyzed against 1× PBS (pH = 7.4) using a 3500 MWCO dialysis membrane at 4 ℃ for 2 h. In Figure 1, equal molar amounts of ionizable lipids were mixed in each group. After the mixing of the ethanol and aqueous buffer, the LNP was immediately diluted with 1× PBS (pH = 7.4) for the assessments. The nanoparticles were diluted into 800 μL 1x PBS, then particle size, polydispersity (PDI) and zeta potential were determined in PBS (0.4 μg mRNA dissolved in 20 μL) using a NanoZS Zetasizer (Malvern Instruments, Malvern, UK). The zeta potential in water was measured by the same diluted method. LogP and logD of the ionizable lipids were obtained from chemaxon website (calculators.cxn.io) according to the structure.

**Measurements of apparent pKa**

Apparent pKa of LNPs was evaluated by TNS assay as described previously^[1]^. Briefly, buffers with pH range 3 - 8.5 in 0.5 increments were prepared using 20 mM sodium phosphate, 25 mM sodium citrate, 20 mM ammonium acetate, and 150 mM NaCl, and then adjusted with 0.1 N NaOH or 0.1 N HCl. Each well of a black 96-well plate was added with 100 μL buffered solution, 10 μL LNP, and 1 μL 0.188 mg/mL TNS. The plates were balanced at room temperature for 20 minutes. Fluorescence intensity was obtained with an excitation at 325 nm and an emission at 435 nm (Spark, Tecan). The normalized fluorescence was evaluated by a curve fit analysis with sigmoid function, resulting in a fluorescence titration curve S. Apparent pKa of each LNPs was calculated as the pH value when the fluorescence intensity reached half of the maximum.

**Animal experiments**

All animal experiments were approved by the Institutional Animal Care and Use Committee of ShanghaiTech University (Approval of Animal Ethical and Welfare Number: 20230527001) and were consistent with the governmental regulations of China for the care and use of animals. Male ICR (15 - 20 g) mice and Male C57BL/6 mice (18-20 g) were purchased from Shanghai Jihui Experimental Animal Breeding Co., Ltd.

**In vivo Luc mRNA delivery**

Six hours after the i.v. injection of LNPs to male ICR mice (n = 3 per group), the mice were injected with D-luciferin potassium (150 mg kg^-1^, i.p.) and imaged using an IVIS Lumina system (Perkin Elmer). Then, the organs were isolated and imaged with the same method.

**Isolation of protein corona enriched in LNPs**

The centrifugal tube was pre-treated with K_2_-EDTA. Mice whole blood was collected from the orbit, followed by centrifugation for 10 min at the speed of 3000 rpm to obtain mouse plasma. Subsequently, the plasma was centrifuged at 3000 rpm for 5 min at 4 ℃ to further remove the residual red blood cells. Finally, the plasma was centrifuged at 13000 g for 5 min at 4 ℃ to remove large particles that may exist. Freshly prepared LNP suspension was incubated with mouse plasma for 1 h at 37 ℃ to mimic the in vivo dynamic protein corona coating process. After centrifuging at 16000 g for 30 min at 4 ℃, the protein corona precipitated at the bottom layer of the solution. Then, the bottom layer was washed three times with cold PBS at 16000 g for 15 min at 4 ℃ to collect the precipitates for further analysis.

**Proteomic assay**

The protocol was adopted from the previously published paper ^[2]^. It is described as follows. A six-fold volume of ice-cold acetone was added to the protein corona precipitate. Subsequently, it was placed at -20 ℃ overnight to precipitate the proteins and dissolve the lipids. After centrifuging at 16000 g, 4 ℃ for 15 min, lipids were removed. The protein mixture was re-suspended in a 1 M Urea/50 mM NH_4_HCO_3_ solution, followed by the adjustment of the concentration of DTT to 5 mM. The solution was incubated at 37 ℃ for 1 h. 500 mM IAM was added to make the final concentration of IAM to be 10 mM, and then the solution was incubated in dark for 45 minutes at room temperature. Trypsin was added to digest the proteins at 37 ℃ overnight. The next morning, the concentration of TFA was adjusted to 0.4% to digest the protein. The pH of the solution was measured and maintained at 2-4. After that, the protein digest was centrifuged at 12000 g for 10 min and the salt removal operation was performed according to the kit (Thermo, 84850).

The extracted peptides were then dried in vacuum and dissolved in 0.1% formic acid for mass spectrometry. Peptides were separated and analyzed on an Easy-nLC 1200 system coupled to an Orbitrap Fusion (Thermo Scientific). About 0.5 µg of peptides were separated in a home-made column (75 µm x 15 cm) packed with C18 AQ (5 µm, 300 Å, Michrom BioResources, Auburn, CA, USA) at a flow rate of 250 nL/min. Mobile phase A (0.1% formic acid) and mobile phase B (0.1% formic acid in 80% ACN) were used to establish a 60 min gradient comprised of 50 min of 6-34% B, 3 min of 34-38% B, 1 min of 38-90% B and 6 min of 90% B. Peptides were then ionized by electrospray at 2.1 kV. A full MS spectrum (350-1400 m/z range) was acquired at a resolution of 120,000 at m/z 200 and a maximum ion accumulation time of 50 ms. Dynamic exclusion was set to 30 s. Resolution for HCD MS/MS spectra was set to 15,000 at m/z 200. The AGC of MS and MS2 were set at 5E5 and 5E4, respectively. Isolation width of 1.6 m/z units and a maximum ion accumulation time of 50 ms were used for MS2. Single and unassigned charged ions were excluded from MS/MS. For HCD, normalized collision energy was set to 28%.

The raw data were processed and analyzed by MaxQuant 1.6.5.0 (Max-Planck-Institute of Biochemistry, Martinsried, Germany) with MS tolerance of 4.5 ppm, and MS/MS tolerance of 20 ppm. The UniProt mouse protein database (release 2016_07, 49863 sequences) and database for proteomics contaminants from MaxQuant were used for database searches. Reversed database searches were used to evaluate false discovery rate (FDR) of peptide and protein identifications. Two missed cleavage sites of trypsin were allowed. Oxidation (M), Acetyl (Protein N-term) and Deamidation (NQ) were set as variable modifications. The FDR of both peptide identification and protein identification is set to be 1%. The option of “Second peptides” and “Match between runs” were enabled.

**Analysis of Proteomic data**

Data was transformed and normalized using variance stabilizing normalization using the DEP 3.18 package of Bioconductor and DEP2 from Github^[3,4]^. The difference between protein absorbed on lung-targeting and liver-targeting LNPs was analyzed using Limma 3.58.1^[5]^. P value was set to be less than 0.05. Fold change (FC) value was set to be greater than 1.2. Differentially enriched proteins with an abundance over 0.1% were selected for downstream analysis. A total of 95 significantly regulated proteins with high abundance were displayed by heatmap using pheatmap 1.0.12. Pearson's test was used to analyze the correlation between these proteins and targeting index (TAR). A p-value less than 0.05 was defined as a correlated protein, and circlize 0.4.16 was used to draw a correlation chord chart^[6]^. The biological functions of these proteins were annotated by GO enrichment. A dotted line chart was used to display the trend of protein expression between different groups. All diagrams in Figure 4 were illustrated by ggplot2 3.4.3^[7]^.

**In vivo toxicity evaluation**

Male C57BL/6 mice (18-20 g) were randomly divided into five groups with n = 3 per group. **A1-3O14** LNPs, **A1-4O14** LNPs, **A1-4O14**+DOTAP LNPs were selected as the lung-targeting, liver-targeting and positive control of lung-targeting groups, respectively. Lipopolysaccharide (5 mg kg^−1^) and PBS were injected as the positive and the negative controls, respectively. We selected a relatively high dose of mRNA (0.8 mg kg^−1^ luciferase mRNA) for i.v. injection. After 6 and 48 h, whole blood was collected and the serum was separated. Then the liver function (AST and ALT), renal function (BUN and CREA) and serum cytokines (IL-6 and TNF-α) were measured by Servicebio, Wuhan, China. Tissue (heart, liver, spleen, lung and kidney) sections with H&E staining were performed by Servicebio, Wuhan, China, and the sections were scanned by Leica Aperio VERSA 8 then analyzed with Image J Fiji and ImageScope x64.

**In vivo accumulation of LNPs/Cy5 luciferase mRNA formulations**

**A1-3O14** LNPs and **A1-4O14** LNPs were formulated at ionizable lipid/DOPE/cholesterol/DMG-PEG2000 molar ratio of 50/10/38.5/1.5. **A1-4O14**+DOTAP were formulated at **A1-4O14**/DOTAP/DOPE/cholesterol/DMG-PEG2000 molar ratio of 25/50/5/19.25/0.75. These LNPs encapsulating Cy5-luc mRNA (0.5 mg kg^−1^) were i.v. injected to ICR male mice. 6 h post administration, the mice were sacrificed and organs were isolated. The fluorescence and bioluminescence of the organs were imaged using the IVIS Spectrum system.

**TEM imaging of LNPs**

To prepare the sample for TEM measurement, 3 μL of LNPs solution was applied to a 300 mesh ultra-thin carbon support film with hydrophilic treatment, then volatilized overnight. The image was collected on a JEM-1400 plus Transmission Electron Microscope.

**Statistical analyses**

Statistical analyses were performed using GraphPad Prism and Origin Pro 2018b. Normal distribution and homogeneity of variance were tested. The statistical analysis of 1C-D was performed using one-way ANOVA Turkey’s multiple comparisons test, substantial differences between groups were indicated by *p < 0.05, **p < 0.01, ***p < 0.001, and ****p < 0.0001. The none significant difference between groups was not shown in figure 1C-1D. The statistical analysis of 1E-F was performed using unpaired t test, substantial differences between groups were indicated by *p < 0.05, **p < 0.01, ***p < 0.001, and ****p < 0.0001. The none significant difference between groups was not shown in figure 1F. For figure 2, 3 and 5, multiple datasets were analyzed by ordinary one-way ANOVA according to Dunnett’s multiple comparisons test. All data are reported as mean ± s.d. (n.s. = not significant, *P < 0.05; **P<0.01; ***P < 0.001; ****P<0.0001).

**Supplementary figures**


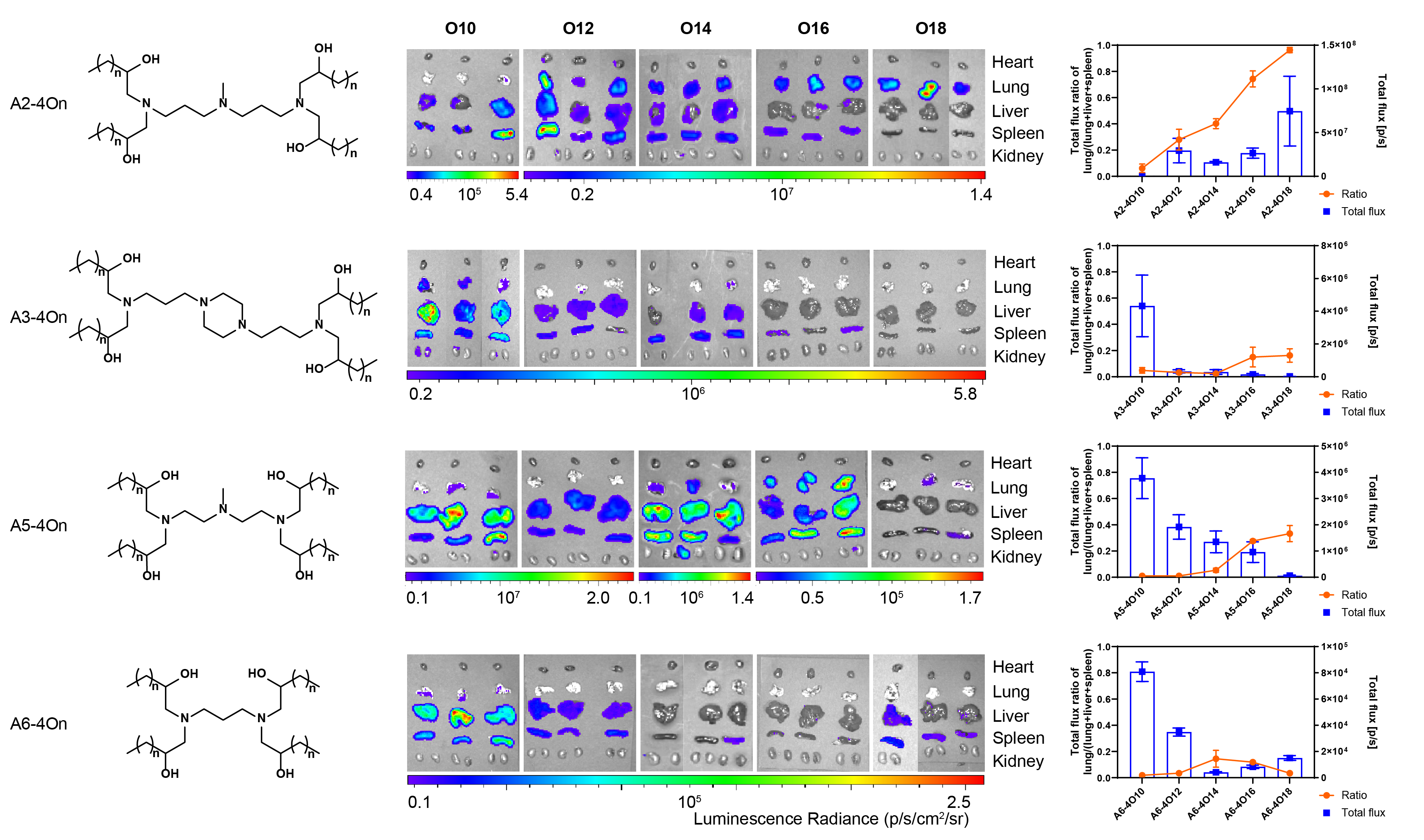


**Figure S1.** Ex vivo bioluminescence images after i.v. administration of A2-4On, A3-4On, A5-4On, and A6-4On LNPs. The total flux in the lung and lung-selectivity of the corresponding LNPs. The mice (n = 3) were administrated with the Luc mRNA-loaded LNPs at a dose of 0.2 mg kg^-1^ through the tail vein. Ex vivo images were taken at 6 h post injection. Lung-selectivity was calculated by total flux of lung/(total flux of lung + total flux of liver + total flux of spleen). Data are presented as mean ± s.d.

**Characterization of ionizable lipids**

**Figure S2.** ^1^H NMR of **A1-3O10**. ^1^H NMR (500 MHz, Chloroform-d) δ 3.84 – 3.41 (m, 12H), 3.02 – 2.67 (m, 4H), 2.67 – 2.53 (m, 2H), 2.50 – 2.40 (m, 2H), 2.34 (dd, *J* = 12.9, 10.3 Hz, 1H), 1.23-1.49 (m, 42H), 0.88 (t, *J* = 6.9 Hz, 9H).


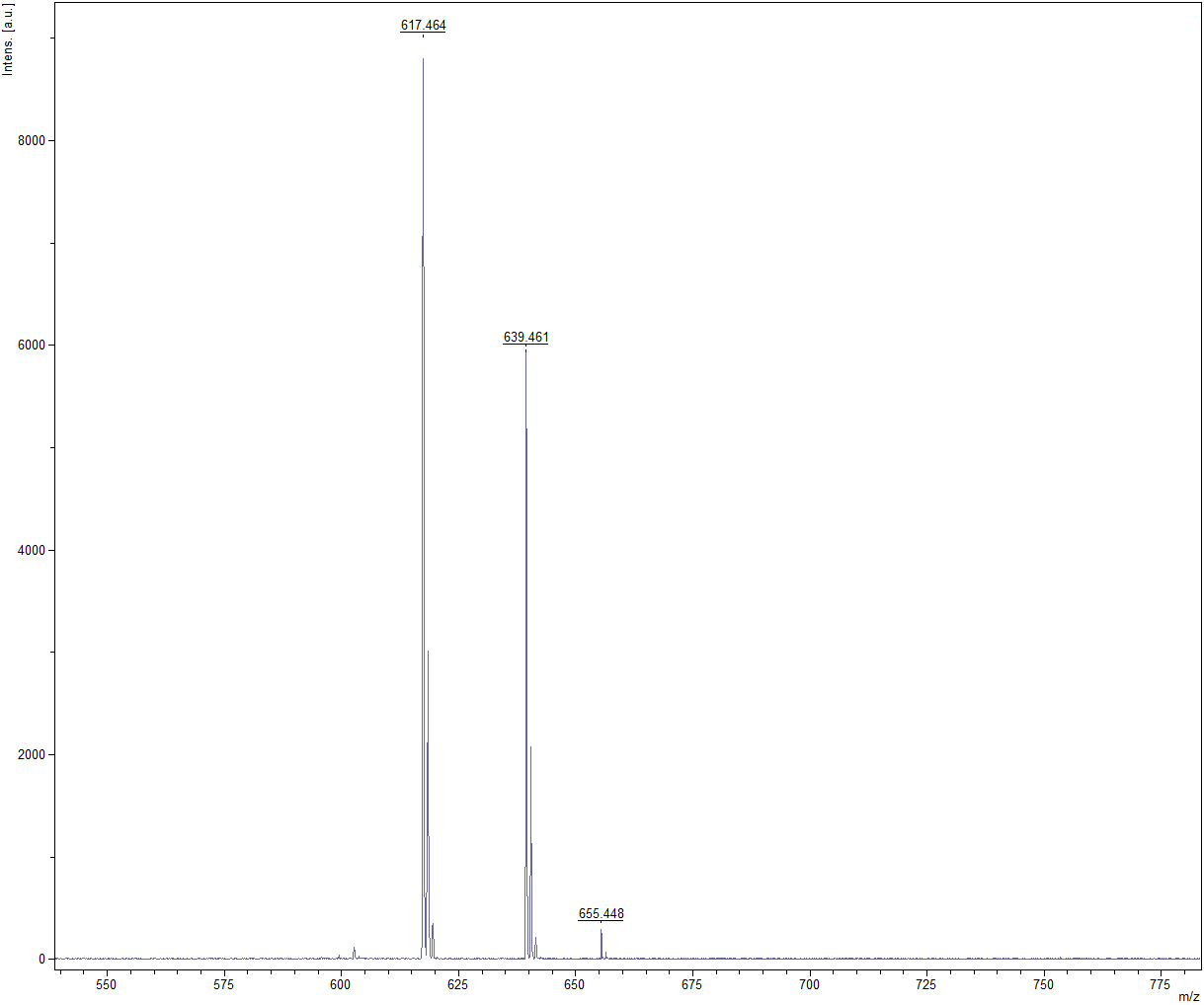


**Figure S3.** Mass spectrum of **A1-3O10**. MALDI-TOF MS m/z of [M + H]^+^ calculated for C_36_H_77_N_2_O_5_: 617.579; Found: 617.464.

**Figure S4.** ^1^H NMR of **A1-4O10**. ^1^H NMR (500 MHz, Chloroform-d) δ 3.71 – 3.37 (m, 12H), 2.77 (m, 3H), 2.60 – 2.49 (m, 2H), 2.46 – 2.20 (m, 7H), 1.56 – 1.37 (m, 8H), 1.34 – 1.19 (m, 48H), 0.88 (t, *J* = 6.7 Hz, 12H).


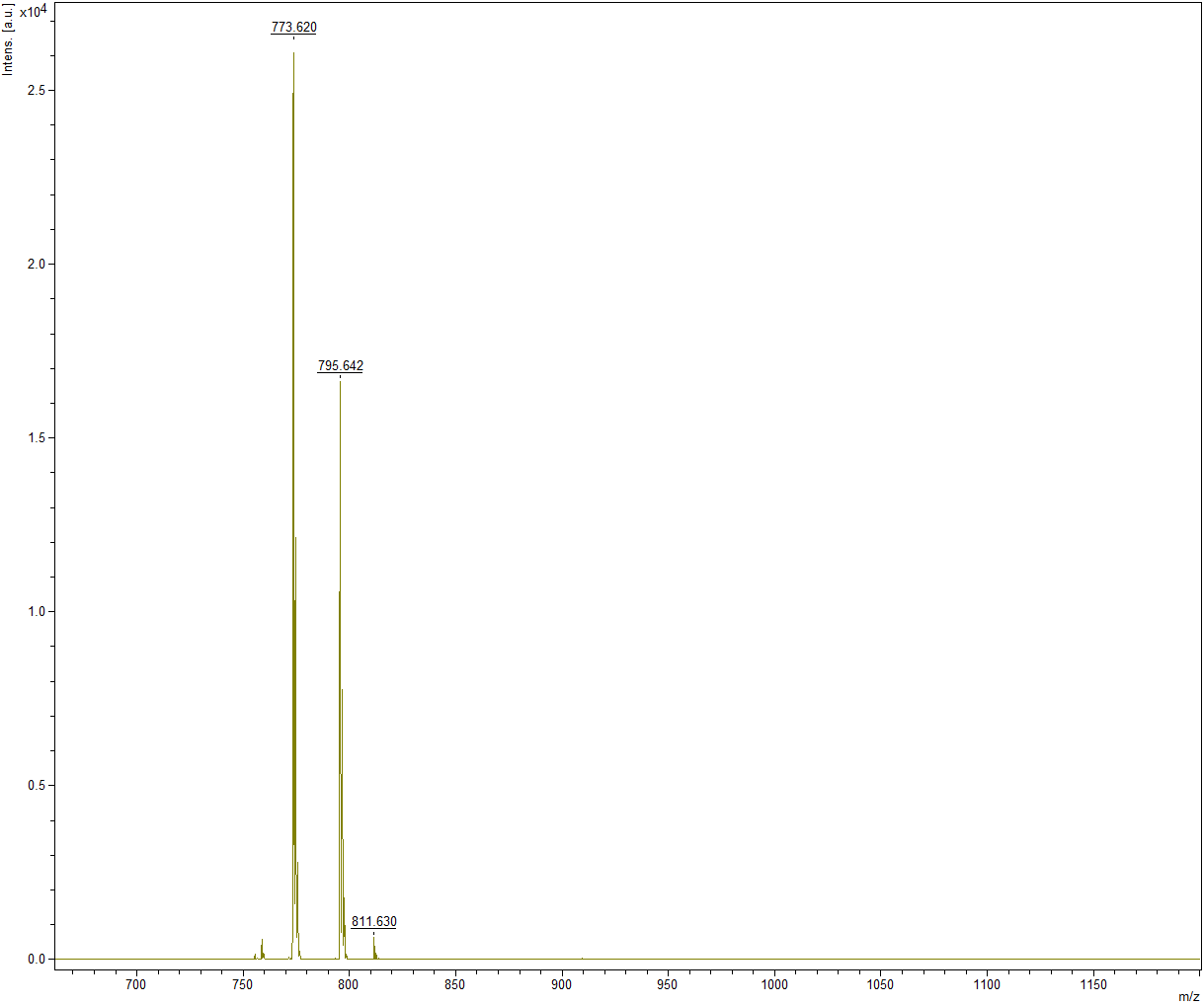


**Figure S5.** Mass spectrum of **A1-4O10**. MALDI-TOF MS m/z of [M + H]^+^ calculated for C_46_H_97_N_2_O_6_: 773.730; Found: 773.620.

**Figure S6.** ^1^H NMR of **A1-3O12**. ^1^H NMR (500 MHz, Chloroform-d) δ 3.78 – 3.47 (m, 11H), 3.02 – 2.31 (m, 10H), 1.39-1.48 (m, 6H), 1.31-1.26 (m, 48H), 0.88 (t, *J* = 6.9 Hz, 9H).

**Figure S7.** Mass spectrum of **A1-3O12**. MALDI-TOF MS m/z of [M + H]^+^ calculated for C_42_H_89_N_2_O_5_: 701.673; Found: 701.530.

**Figure S8.** ^1^H NMR of **A1-4O12**. ^1^H NMR (500 MHz, Chloroform-d) δ 3.76 – 3.26 (m, 12H), 3.05 – 2.21 (m, 12H), 1.57 – 1.36 (m, 8H), 1.36 – 1.17 (m, 64H), 0.88 (t, *J* = 6.9 Hz, 12H).


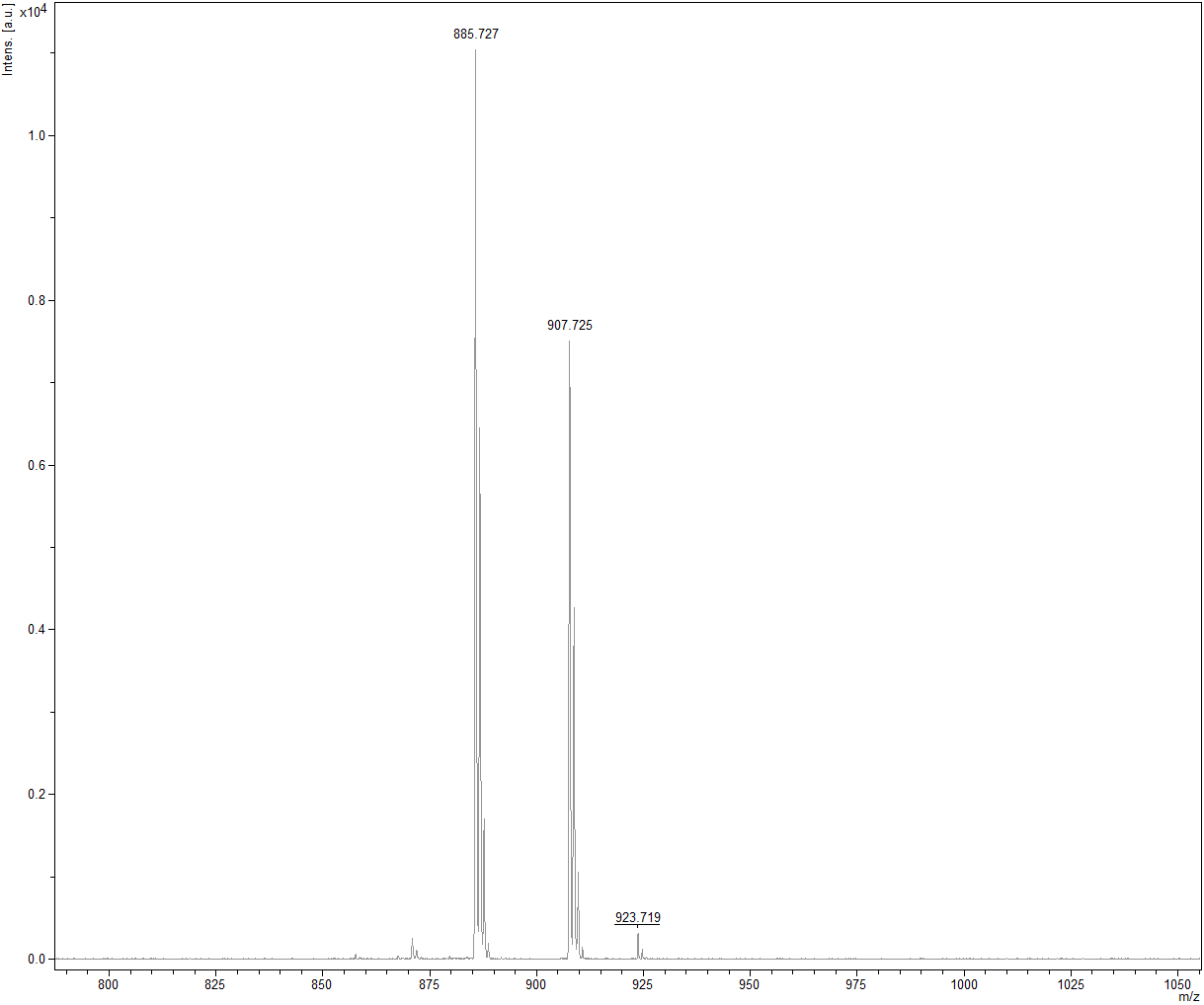


**Figure S9.** Mass spectrum of **A1-4O12**. MALDI-TOF MS m/z of [M + H]^+^ calculated for C_54_H_113_N_2_O_6_: 885.855; Found: 885.727.

**Figure S10.** ^1^H NMR of **A1-3O14**. ^1^H NMR (500 MHz, CDCl_3_) δ 3.86 – 3.42 (m, 11H), 3.08 – 2.74 (m, 5H), 2.69 – 2.56 (m, 2H), 2.47 – 2.32 (m, 3H), 1.50 – 1.40 (m, 6H), 1.35-1.14 (m, 60H), 0.88 (t, *J* = 6.8 Hz, 9H).


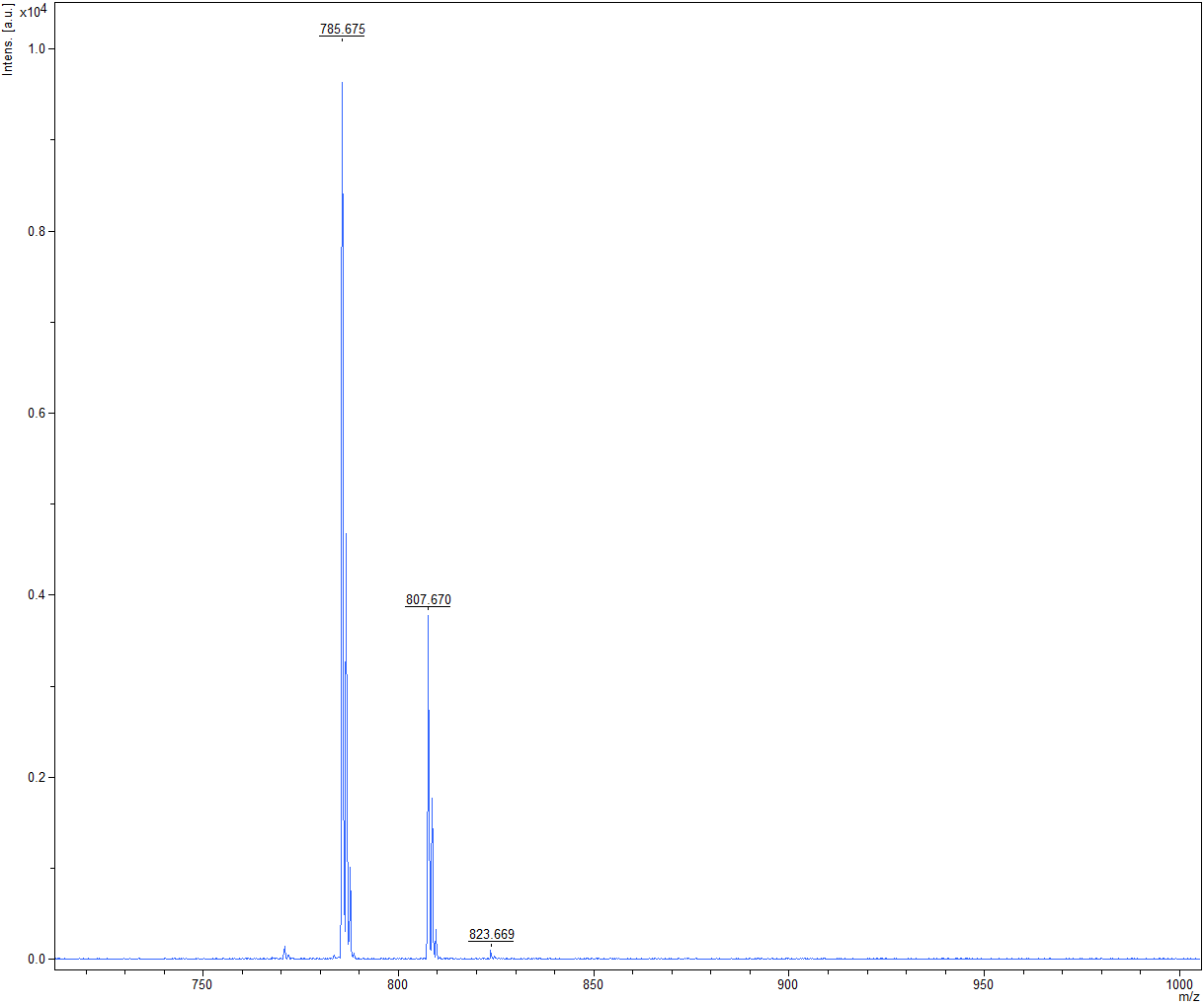


**Figure S11.** Mass spectrum of **A1-3O14**. MALDI-TOF MS m/z of [M + H]^+^ calculated for C_48_H_101_N_2_O_5_: 785.767; Found: 785.675.

**Figure S12.** ^1^H NMR of **A1-4O14**. ^1^H NMR (500 MHz, Chloroform-d) δ 3.73 – 3.36 (m, 12H), 3.01 – 2.65 (m, 4H), 2.64 – 2.50 (m, 2H), 2.48 – 2.30 (m, 6H), 1.60 – 1.12 (m, 88H), 0.88 (t, *J* = 6.8 Hz, 12H).


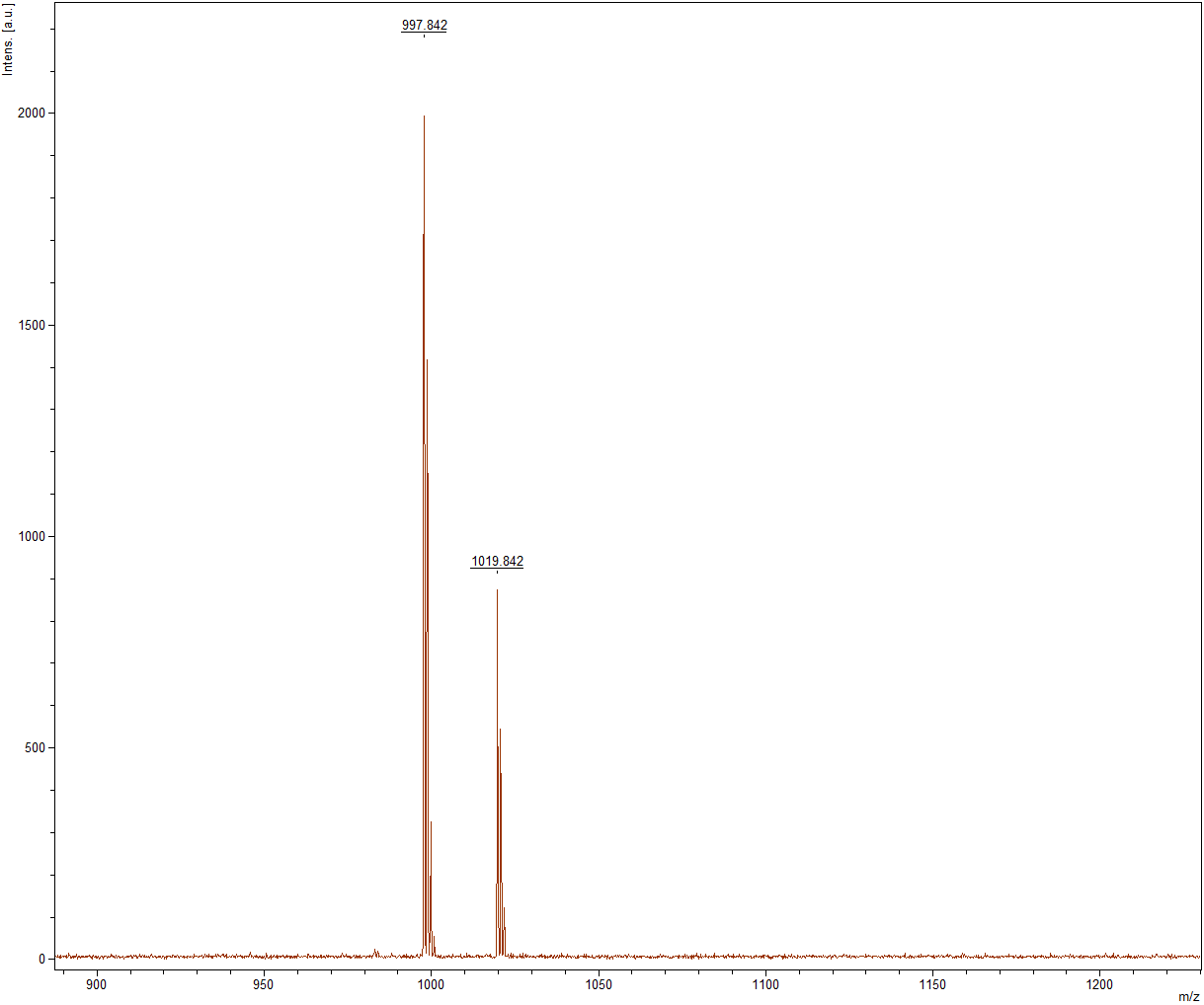


**Figure S13.** Mass spectrum of **A1-4O14**. MALDI-TOF MS m/z of [M + H]^+^ calculated for C_62_H_129_N_2_O_6_: 997.981; Found: 997.842.

**Figure S14.** ^1^H NMR of **A1-3O16**. ^1^H NMR (500 MHz, Chloroform-d) δ 4.08 – 3.68 (m, 8H), 3.61 – 3.36 (m, 4H), 3.21 – 2.56 (m, 6H), 2.54 – 2.22 (m, 3H), 1.59 – 1.01 (m, 78H), 0.88 (t, *J* = 6.9 Hz, 9H).


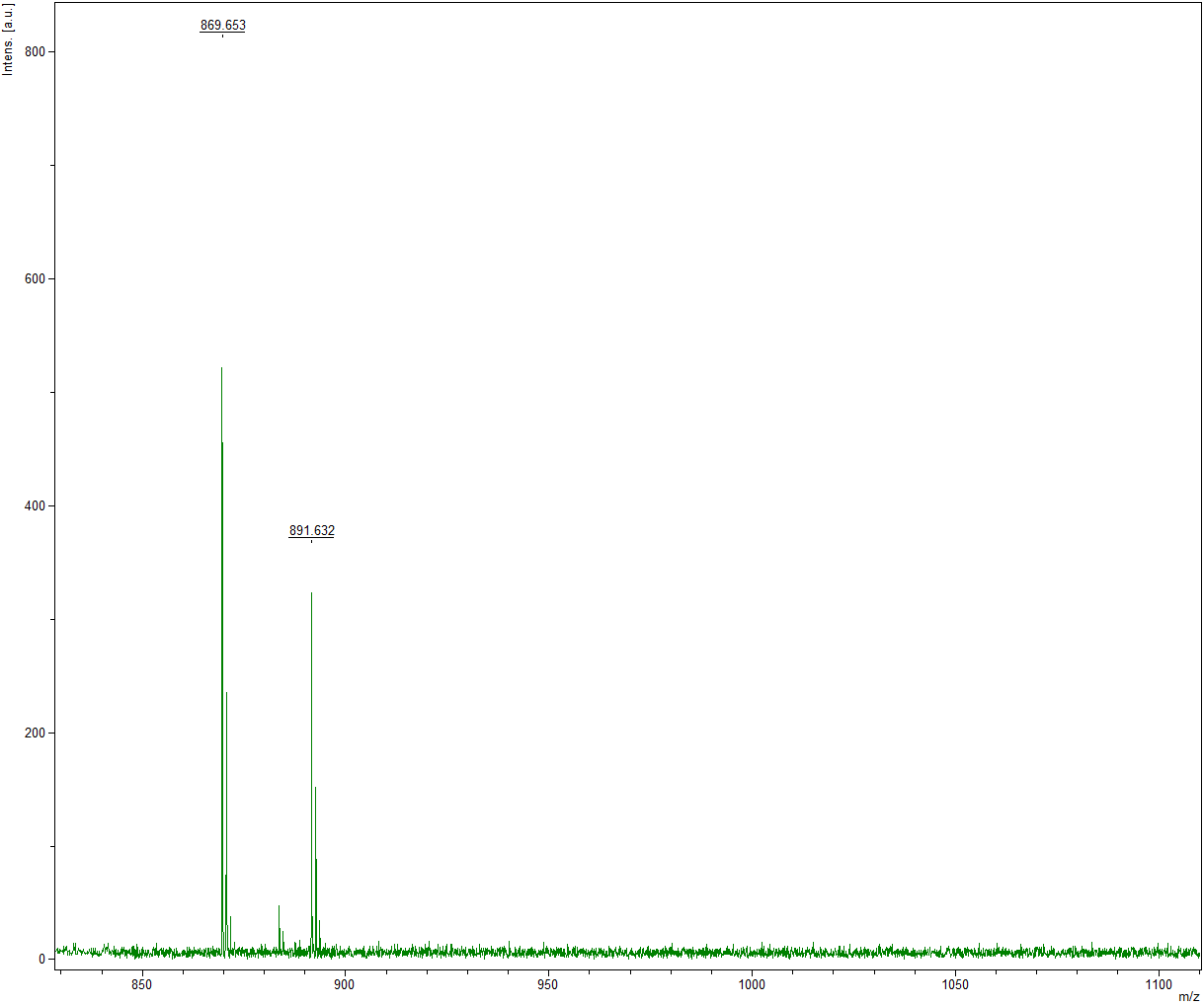


**Figure S15.** Mass spectrum of **A1-3O16**. MALDI-TOF MS m/z of [M + H]^+^ calculated for C_54_H_113_N_2_O_5_: 869.860; Found: 869.653.

**Figure S16.** ^1^H NMR of **A1-4O16**: ^1^H NMR (500 MHz, Chloroform-d) δ 3.71 – 3.38 (m, 12H), 3.00 – 2.64 (m, 4H), 2.54 (ddd, *J* = 23.2, 13.3, 2.7 Hz, 2H), 2.46 – 2.24 (m, 6H), 1.56 – 1.09 (m, 104H), 0.88 (t, *J* = 6.9 Hz, 12H).


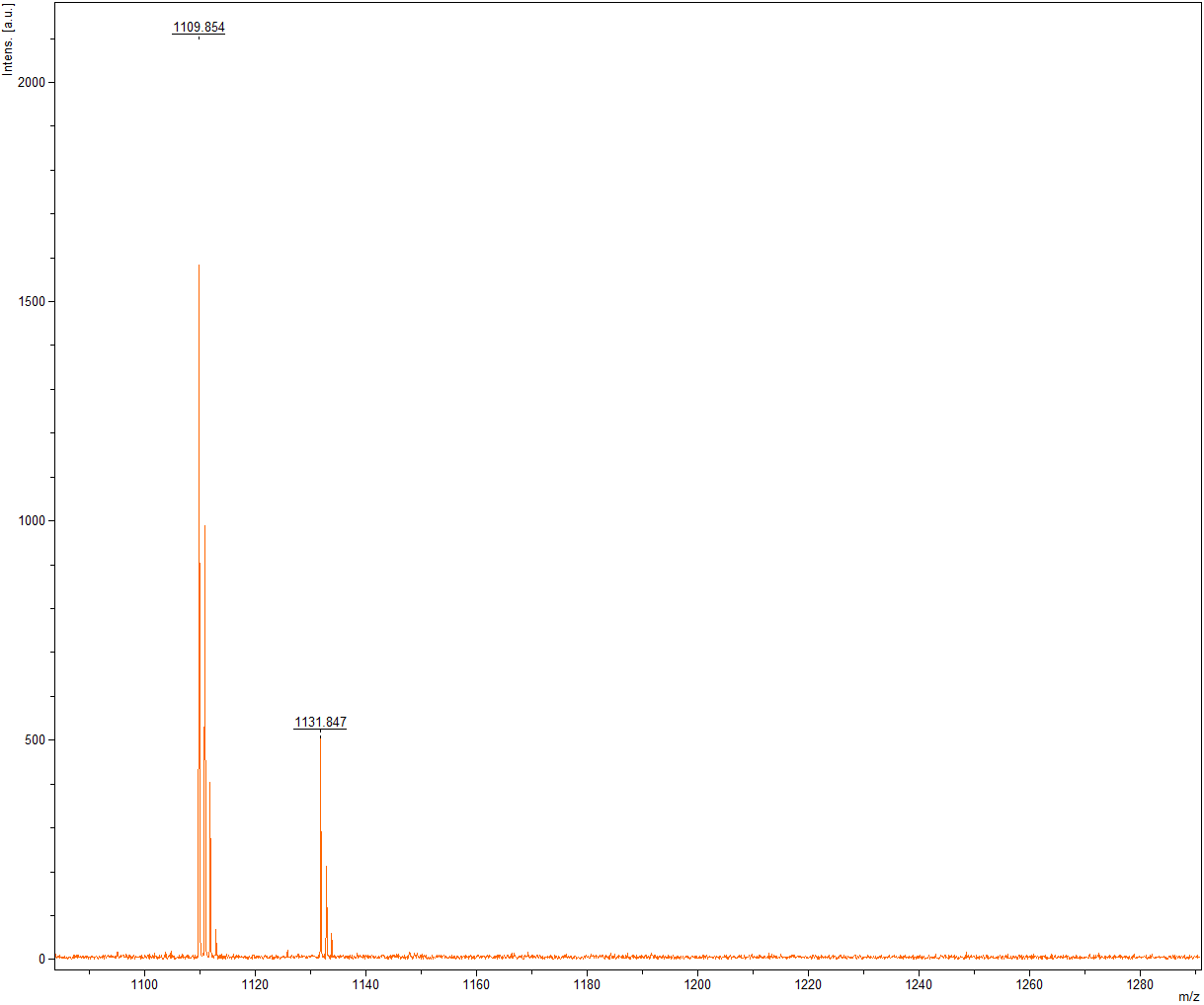


**Figure S17.** Mass spectrum of **A1-4O16**. MALDI-TOF MS m/z of [M + H]^+^ calculated for C_70_H_145_N_2_O_6_: 1110.106; Found: 1109.854.

**Figure S18.** ^1^H NMR of **A1-3O18**: ^1^H NMR (500 MHz, Chloroform-d) δ 3.78 – 3.28 (m, 12H), 2.97 – 2.76 (m, 4H), 2.65 – 2.50 (m, 2H), 2.47 – 2.25 (m, 3H), 1.52 – 1.38 (m, 6H), 1.37 – 1.08 (m, 84H), 0.88 (t, *J* = 6.9 Hz, 9H).


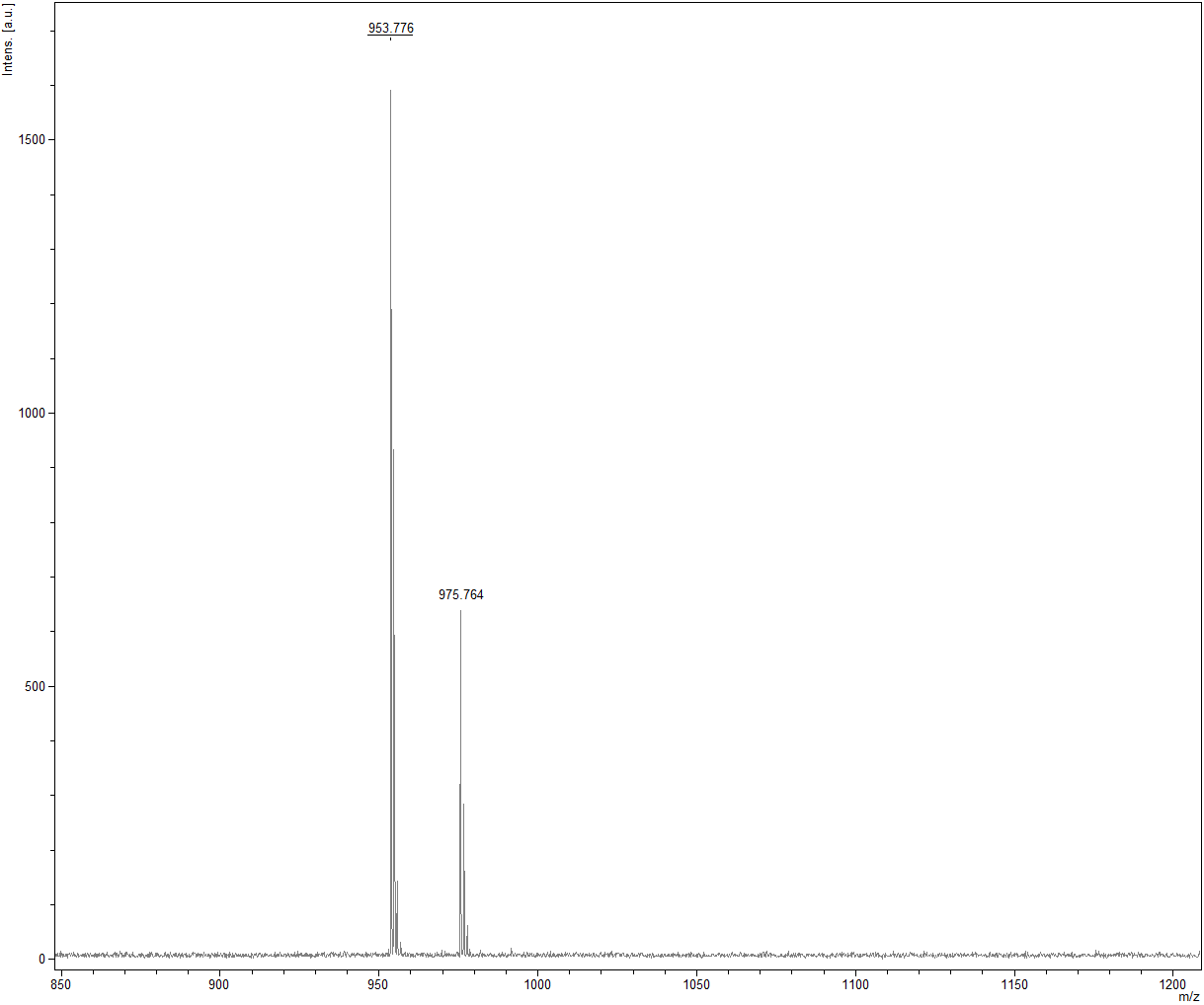


**Figure S19** Mass spectrum of **A1-3O18**. MALDI-TOF MS m/z of [M + H]^+^ calculated for C_60_H_125_N_2_O_5_: 953.954; Found: 953.776.

**Figure S20.** ^1^H NMR of **A1-4O18**. ^1^H NMR (500 MHz, Chloroform-d) δ 3.75 – 3.36 (m, 12H), 3.04 – 2.65 (m, 4H), 2.62 – 2.50 (m, 2H), 2.48 – 2.22 (m, 6H), 1.57 – 1.07 (m, 120H), 0.88 (t, *J* = 6.9 Hz, 12H).


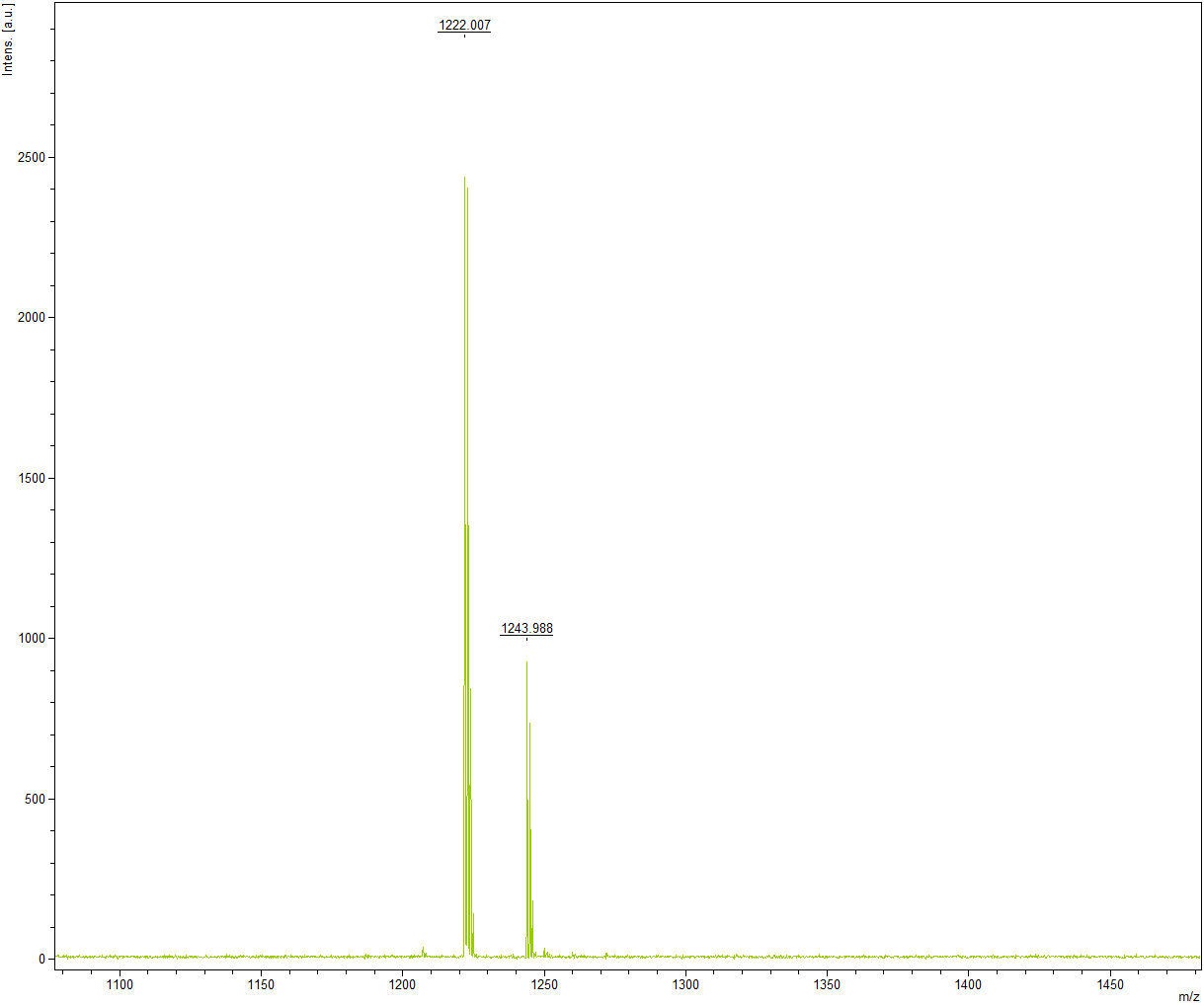


**Figure S21.** Mass spectrum of **A1-4O18**. MALDI-TOF MS m/z of [M + H]^+^ calculated for C_78_H_161_N_2_O_6_: 1222.231; Found: 1222.007.

**Figure S22.** ^1^H NMR of **A2-3O10**: ^1^H NMR (500 MHz, Chloroform-d) δ 3.71 – 3.57 (m, 3H), 2.77 – 2.55 (m, 5H), 2.51 – 2.27 (m, 9H), 2.19 (s, 3H), 1.70 (dt, *J* = 13.9, 7.2 Hz, 4H), 1.52 – 1.10 (m, 42H), 0.88 (t, *J* = 7.0 Hz, 9H).


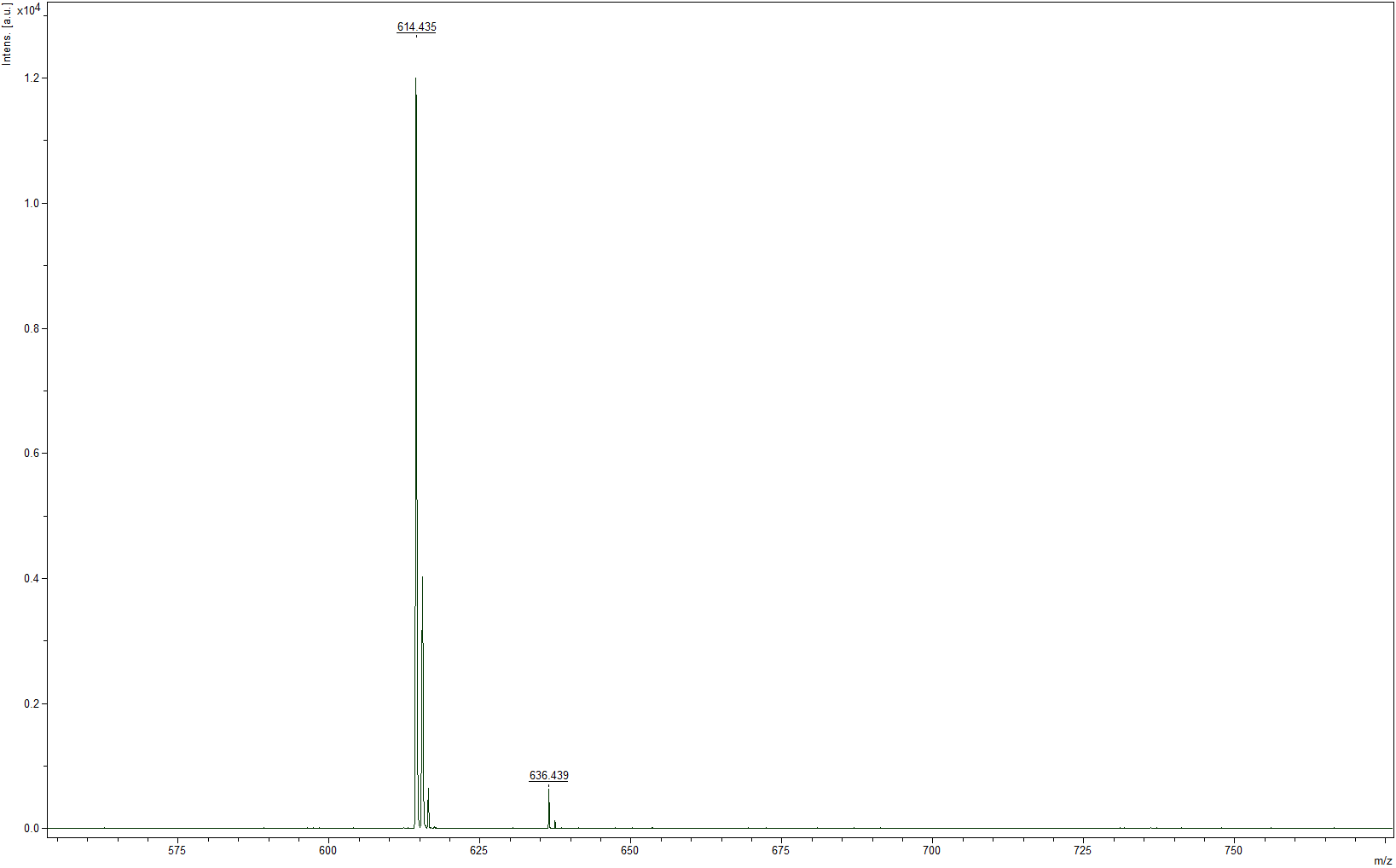


**Figure S23.** Mass spectrum of **A2-3O10**. MALDI-TOF MS m/z of [M + H]^+^ calculated for C_37_H_80_N_3_O_3_: 614.615; Found: 614.435.

**Figure S24.** ^1^H NMR of **A2-4O10**. ^1^H NMR (500 MHz, Chloroform-d) δ 3.74 – 3.49 (m, 4H), 2.76 – 2.12 (m, 19H), 1.86 – 1.54 (m, 4H), 1.50 – 1.17 (m, 56H), 0.88 (t, *J* = 6.9 Hz, 12H).


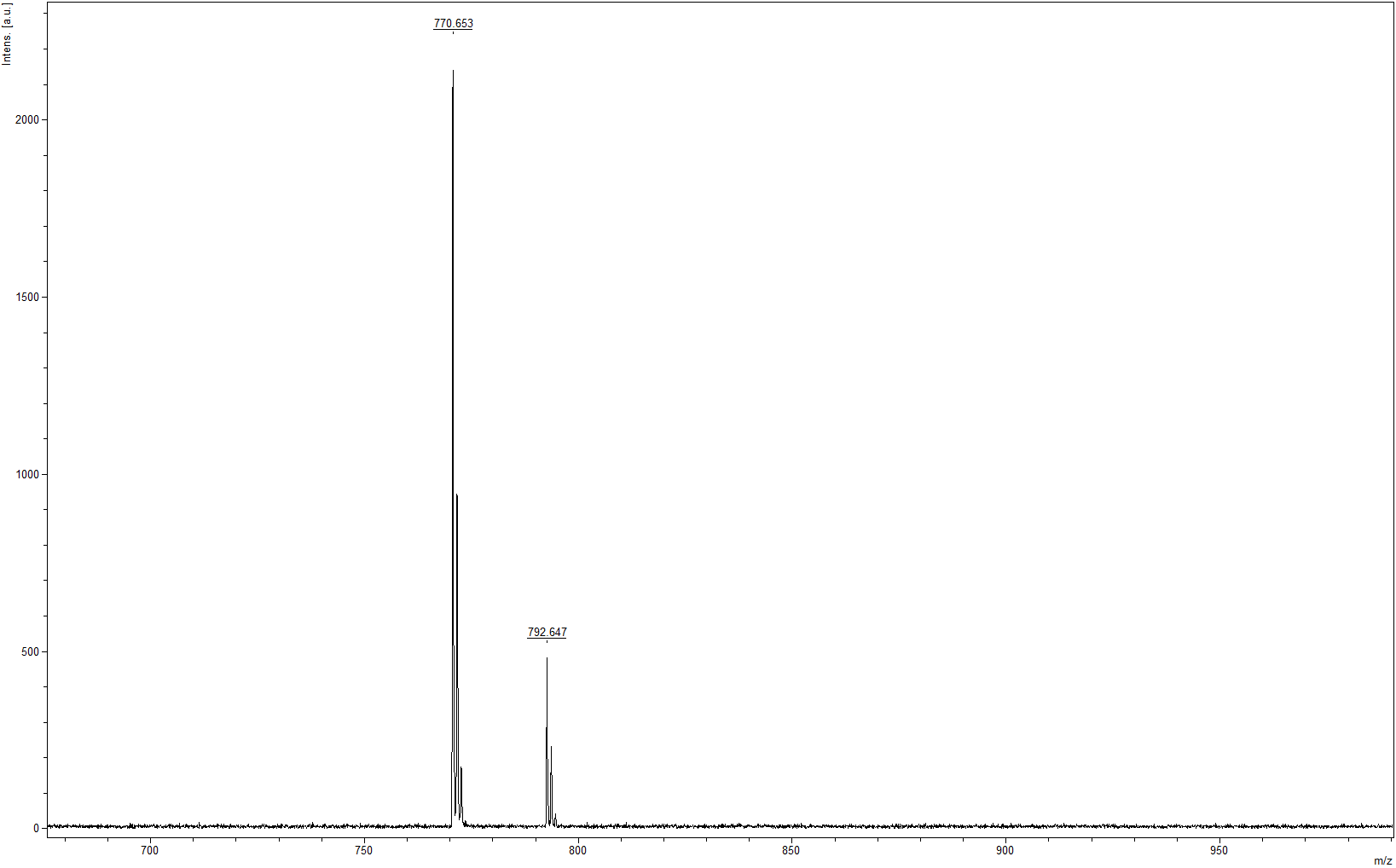


**Figure S25.** Mass spectrum of **A2-4O10**. MALDI-TOF MS m/z of [M + H]^+^ calculated for C_47_H_100_N_3_O_4_: 770.767; Found: 770.653.

**Figure S26.** ^1^H NMR of **A2-3O12**: ^1^H NMR (500 MHz, Chloroform-d) δ 3.77 – 3.54 (m, 3H), 2.89 – 2.55 (m, 5H), 2.53 – 2.27 (m, 9H), 2.19 (s, 3H), 1.78 – 1.55 (m, 4H), 1.50 – 1.13 (m, 54H), 0.88 (t, *J* = 6.9 Hz, 9H).


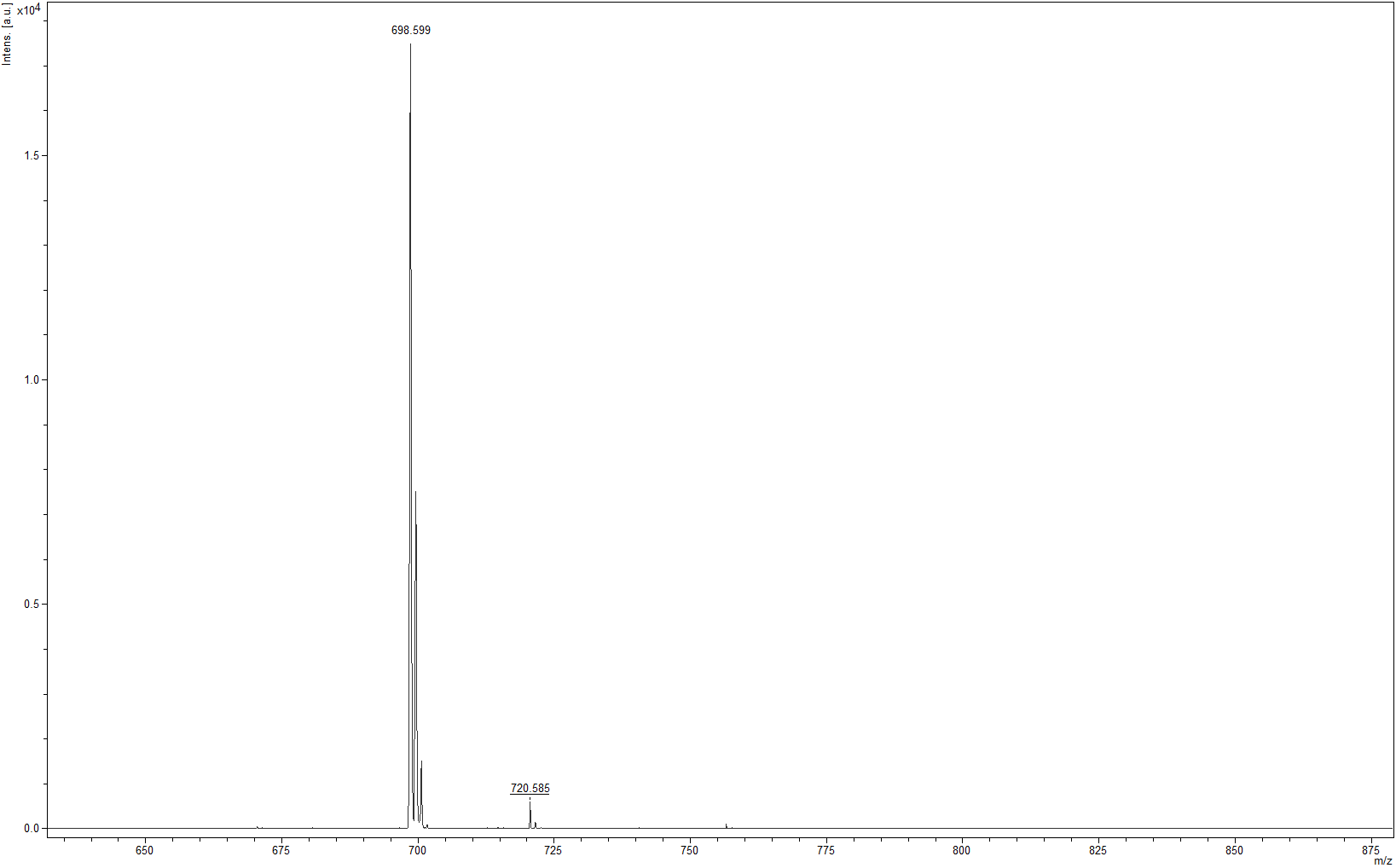


**Figure S27.** Mass spectrum of **A2-3O12**. MALDI-TOF MS m/z of [M + H]^+^ calculated for C_43_H_92_N_3_O_3_: 698.709; Found: 698.599.

**Figure S28.** ^1^H NMR of **A2-4O12**. ^1^H NMR (500 MHz, Chloroform-d) δ 3.62 (dqd, *J* = 20.8, 6.8, 5.9, 3.1 Hz, 4H), 2.83 – 2.15 (m, 19H), 1.88 – 1.55 (m, 4H), 1.52 – 0.99 (m, 72H), 0.88 (t, *J* = 6.9 Hz, 12H).


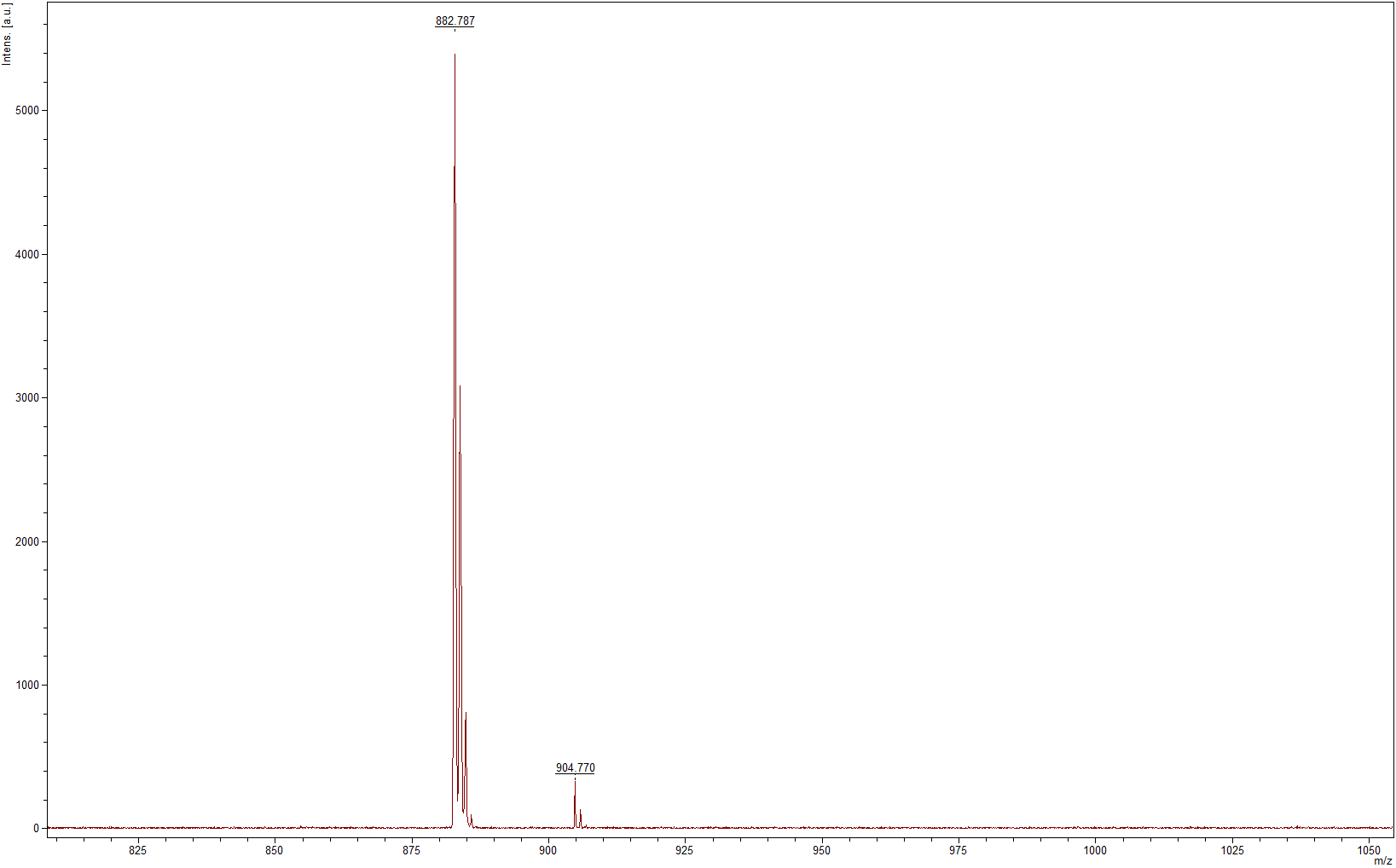


**Figure S29.** Mass spectrum of **A2-4O12**. MALDI-TOF MS m/z of [M + H]^+^ calculated for C_55_H_116_N_3_O_4_: 882.892; Found: 882.787.

**Figure S30.** ^1^H NMR of **A2-3O14**. ^1^H NMR (500 MHz, CDCl_3_) δ 3.74 – 3.54 (m, 3H), 2.78 – 2.52 (m, 5H), 2.50 – 2.26 (m, 9H), 2.18 (s, 3H), 1.75 – 1.52 (m, 4H), 1.47 – 1.11 (m, 66H), 0.88 (t, *J* = 6.9 Hz, 9H).


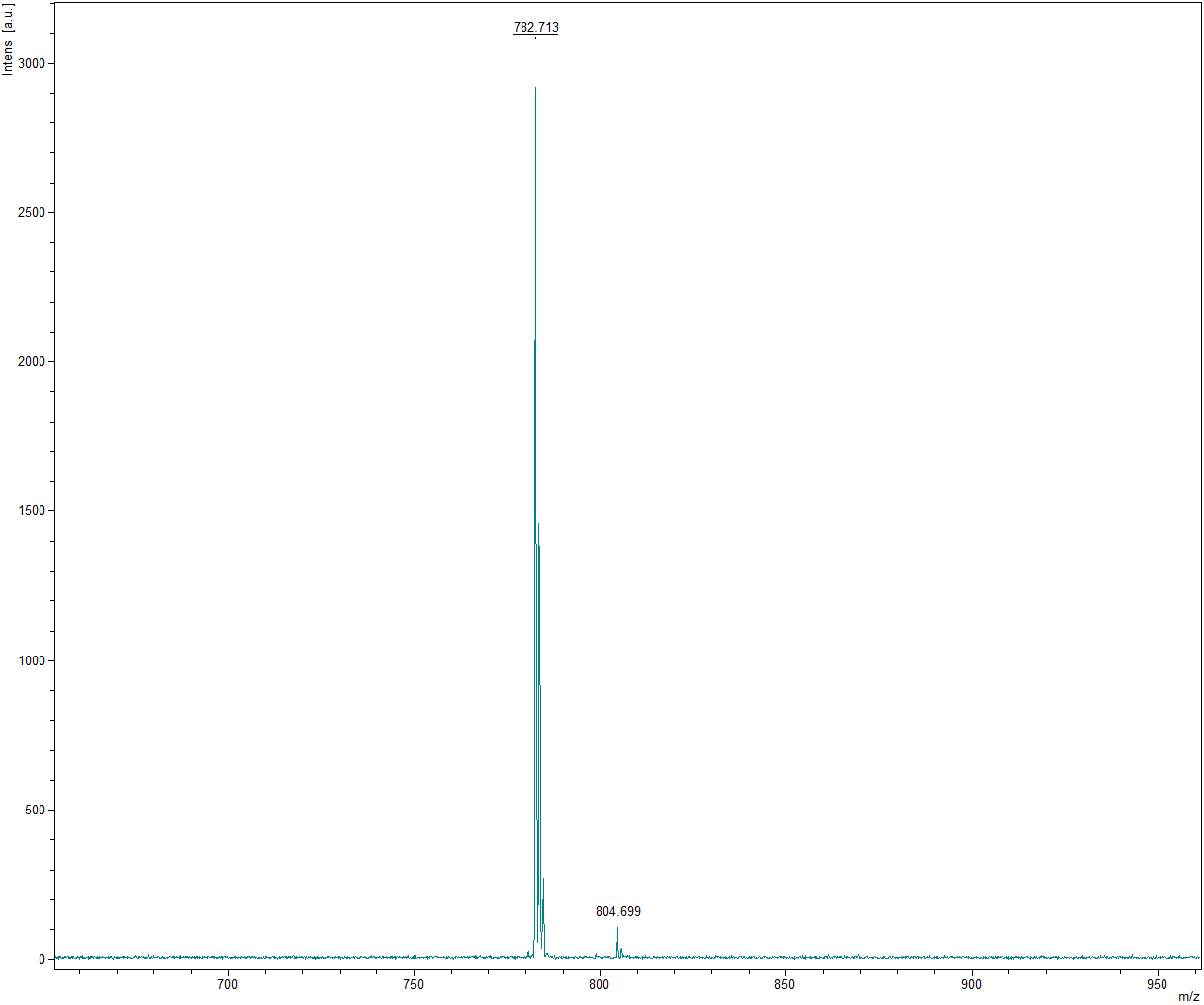


**Figure S31.** Mass spectrum of **A2-3O14**. MALDI-TOF MS m/z of [M + H]^+^ calculated for C_49_H_104_N_3_O_3_: 782.803; Found: 782.713.

**Figure S32.** ^13^C NMR of **A2-3O14**. ^13^C NMR (126 MHz, CDCl_3_) δ 70.21, 69.28, 67.70, 62.40, 61.24, 56.12, 55.38, 54.45, 53.55, 48.04, 41.95, 35.27, 35.07, 32.06, 29.82, 29.50, 26.73, 25.87, 24.70, 22.82, 14.26.

**Figure S33.** ^1^H NMR of **A2-4O14**. ^1^H NMR (500 MHz, CDCl_3_) δ 3.75 – 3.54 (m, 4H), 2.83 – 2.20 (m, 19H), 1.83 – 1.54 (m, 4H), 1.50 – 1.18 (m, 88H), 0.88 (t, *J* = 6.9 Hz, 12H).


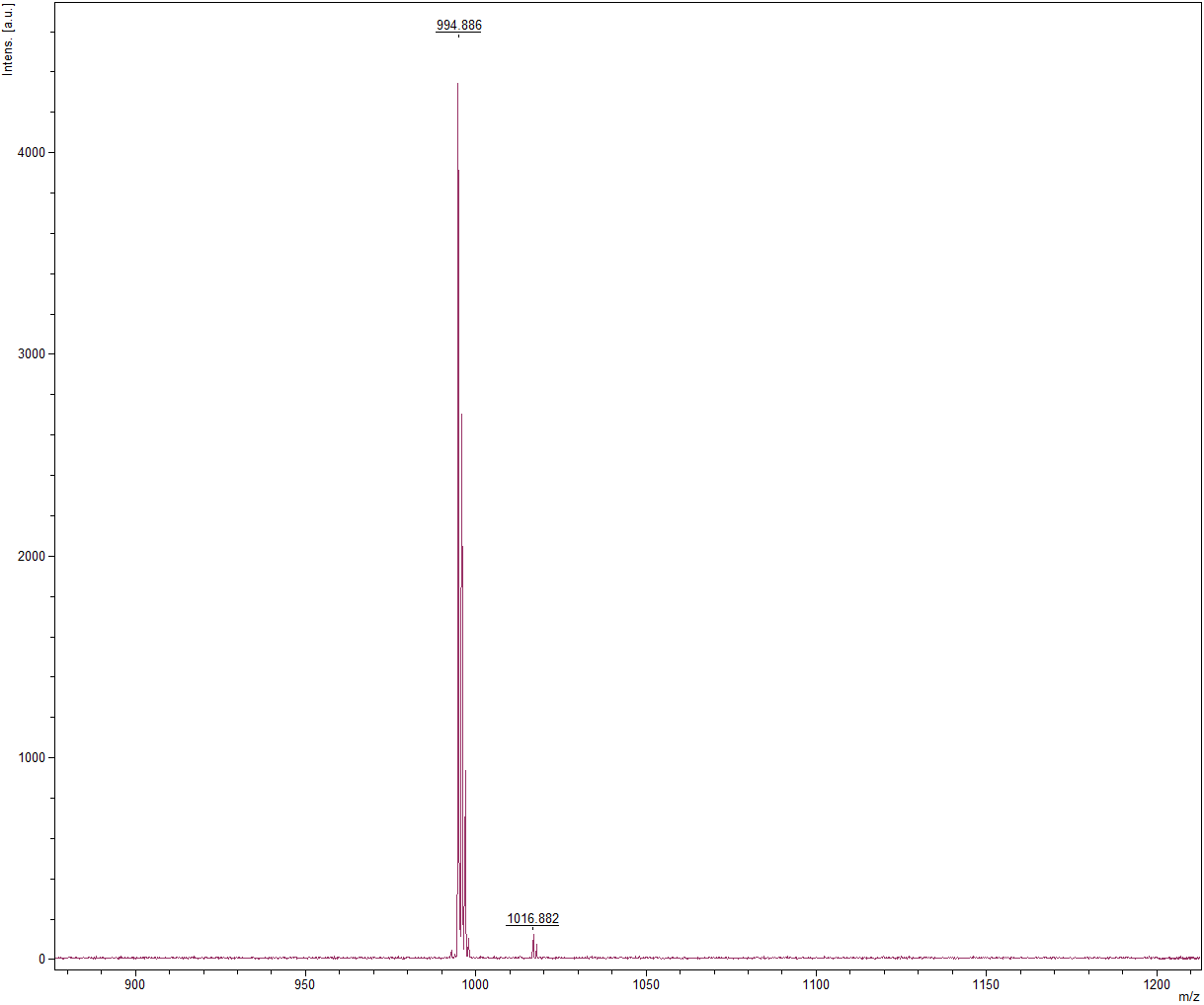


**Figure S34.** Mass spectrum of **A2-4O14**. MALDI-TOF MS m/z of [M + H]^+^ calculated for C_63_H_132_N_3_O_4_: 995.017; Found: 994.886.

**Figure S35.** ^1^H NMR of **A2-3O16**. ^1^H NMR (500 MHz, Chloroform-d) δ 3.81 – 3.50 (m, 3H), 2.93 – 2.54 (m, 5H), 2.51 – 2.23 (m, 9H), 2.19 (s, 3H), 1.85 – 1.54 (m, 4H), 1.19-1.46 (m, 78H), 0.88 (t, *J* = 6.9 Hz, 9H).


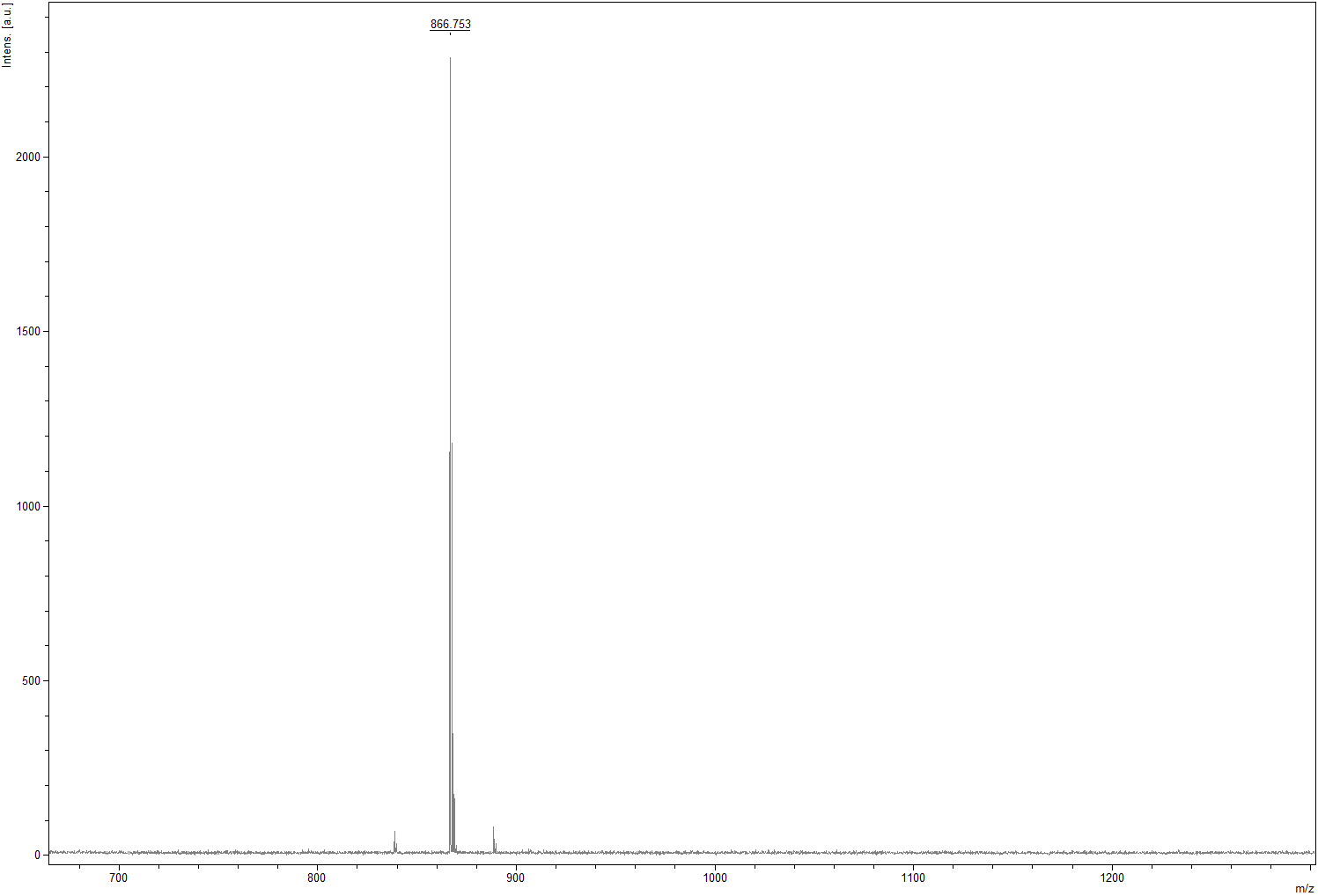


**Figure S36.** Mass spectrum of **A2-3O16**. MALDI-TOF MS m/z of [M + H]^+^ calculated for C_55_H_116_N_3_O_3_: 866.897; Found: 866.753.

^^

**Figure S37.** ^1^H NMR of **A2-4O16**. ^1^H NMR (500 MHz, Chloroform-d) δ 3.69 – 3.55 (m, 4H), 2.74 – 2.14 (m, 19H), 1.78 – 1.54 (m, 4H), 1.14-1.45 (m, 104H), 0.88 (t, *J* = 6.9 Hz, 12H).


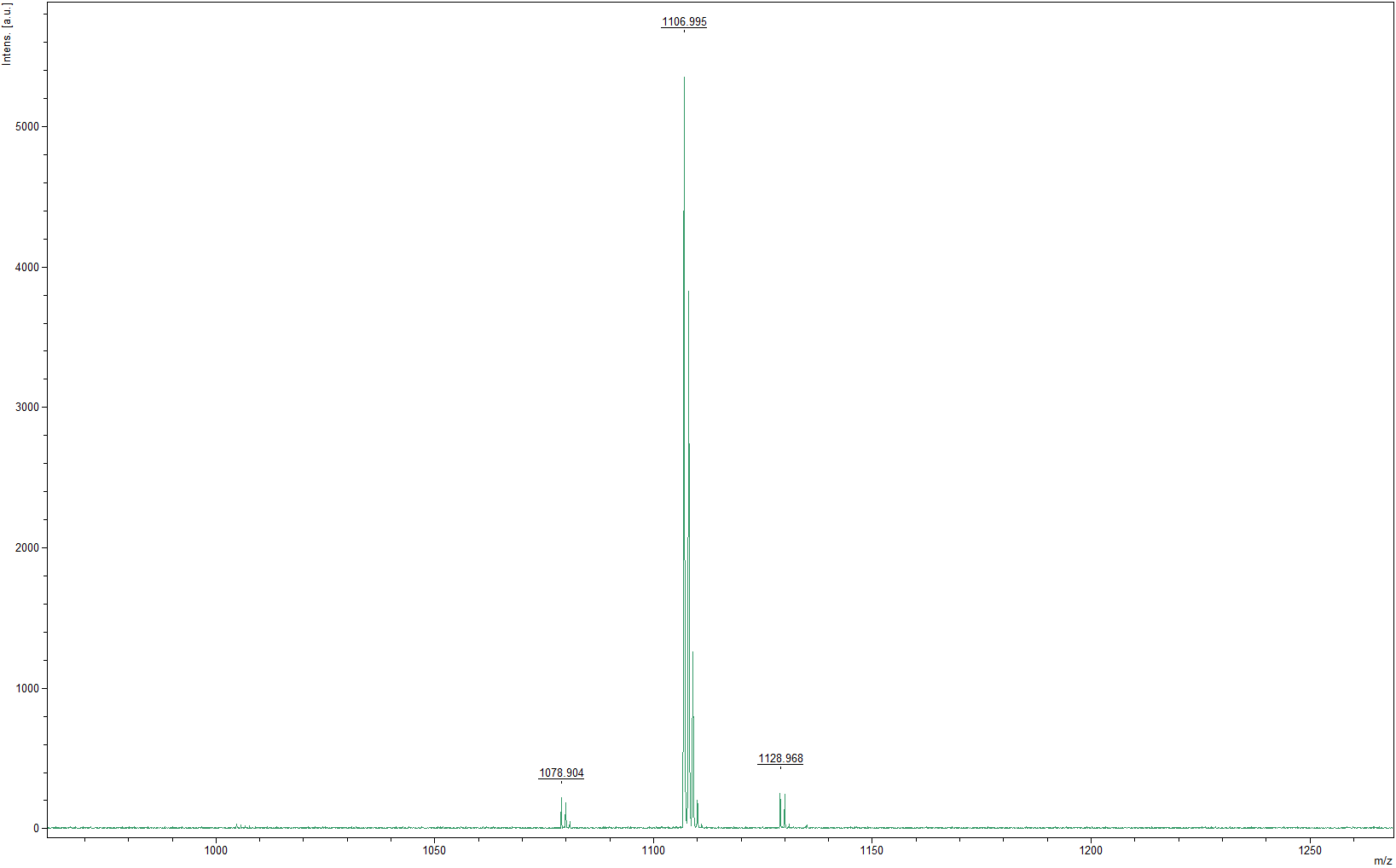


**Figure S38.** Mass spectrum of **A2-4O16**. MALDI-TOF MS m/z of [M + H]^+^ calculated for C_73_H_148_N_3_O_4_: 1107.143; Found: 1106.995.

**Figure S39.** ^1^H NMR of **A2-3O18**: ^1^H NMR (500 MHz, Chloroform-d) δ 3.70 – 3.55 (m, 3H), 2.87 – 2.55 (m, 5H), 2.54 – 2.24 (m, 9H), 2.20 (s, 3H), 1.77-1.60 (m, 4H), 1.46 – 1.15 (m, 90H), 0.88 (t, *J* = 6.9 Hz, 9H).


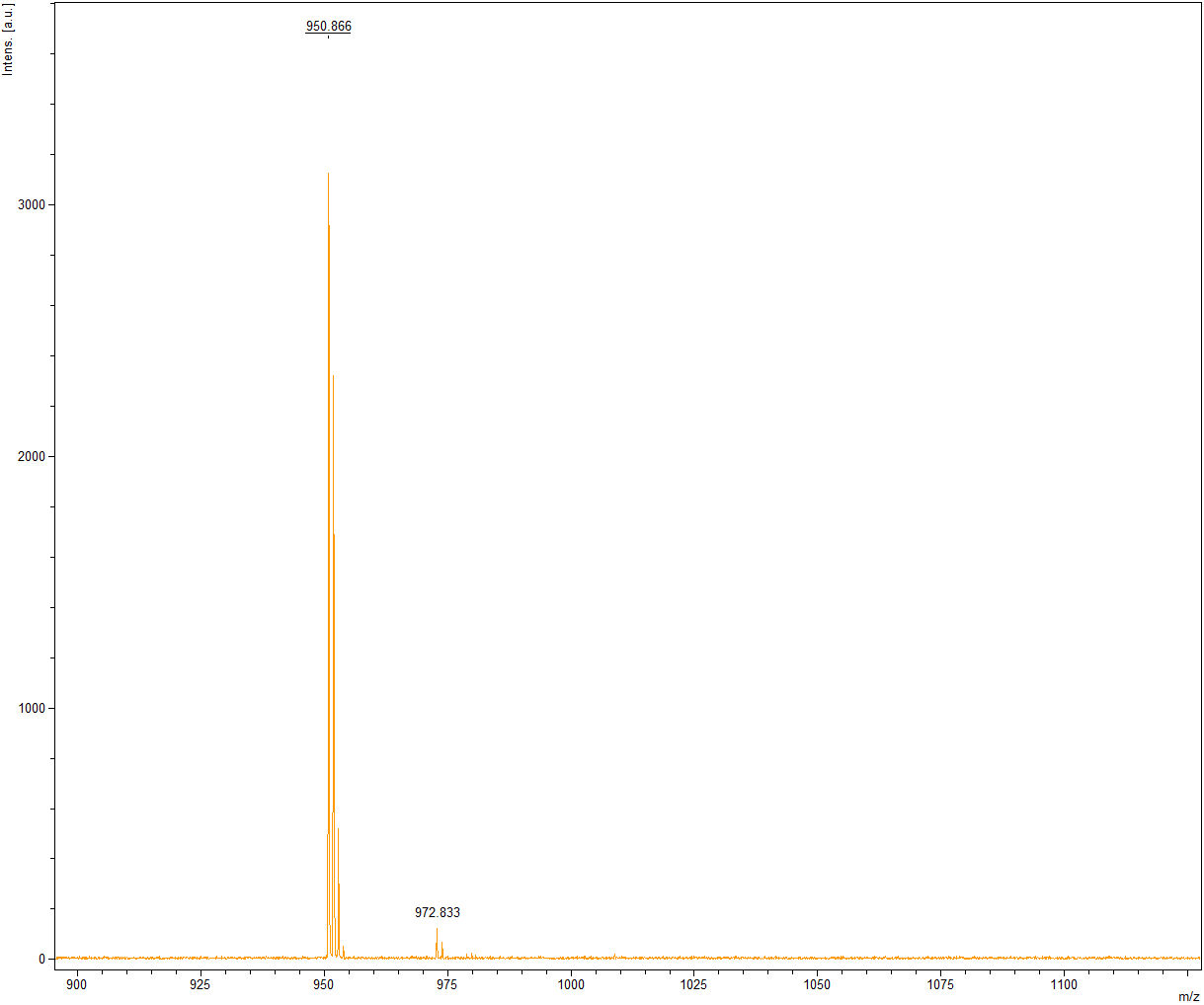


**Figure S40.** Mass spectrum of **A2-3O18**. MALDI-TOF MS m/z of [M + H]^+^ calculated for C_61_H_128_N_3_O_3_: 950.991; Found: 950.866.

**Figure S41.** ^1^H NMR of **A2-4O18**: ^1^H NMR (500 MHz, Chloroform-d) δ 3.73 – 3.49 (m, 4H), 2.74 – 2.10 (m, 19H), 1.73 – 1.56 (m, 4H), 1.45 – 1.13 (m, 120H), 0.88 (t, *J* = 6.9 Hz, 12H).


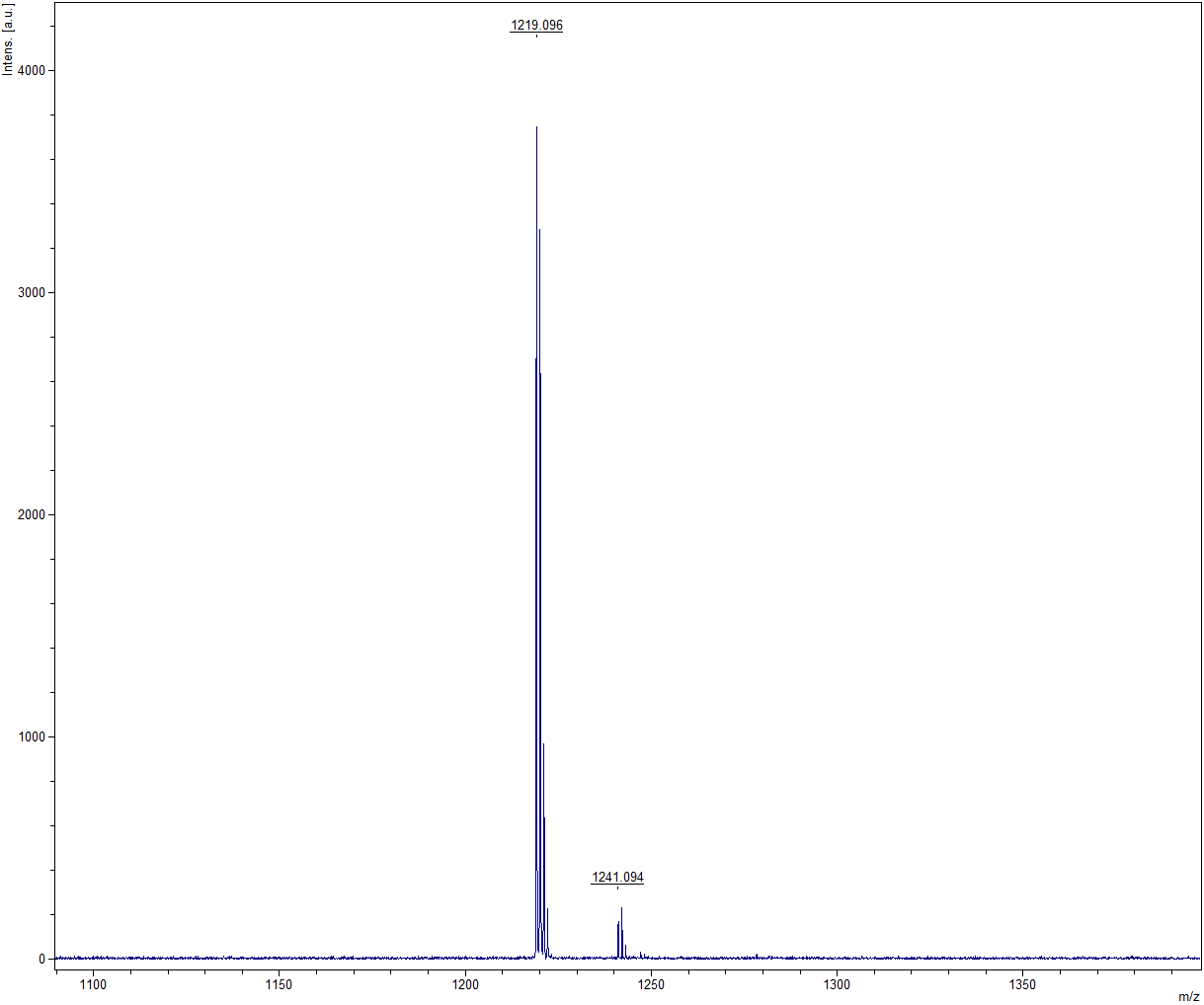


**Figure S42.** Mass spectrum of **A2-4O18**. MALDI-TOF MS m/z of [M + H]^+^ calculated for C_79_H_164_N_3_O_4_: 1219.268; Found: 1219.096.

**Figure S43.** ^1^H NMR of **A3-3O10**. ^1^H NMR (500 MHz, Chloroform-d) δ 3.69 – 3.54 (m, 3H), 3.05 – 2.10 (m, 22H), 1.75 – 1.58 (m, 4H), 1.49 – 1.21 (m, 42H), 0.88 (t, *J* = 6.8 Hz, 9H).


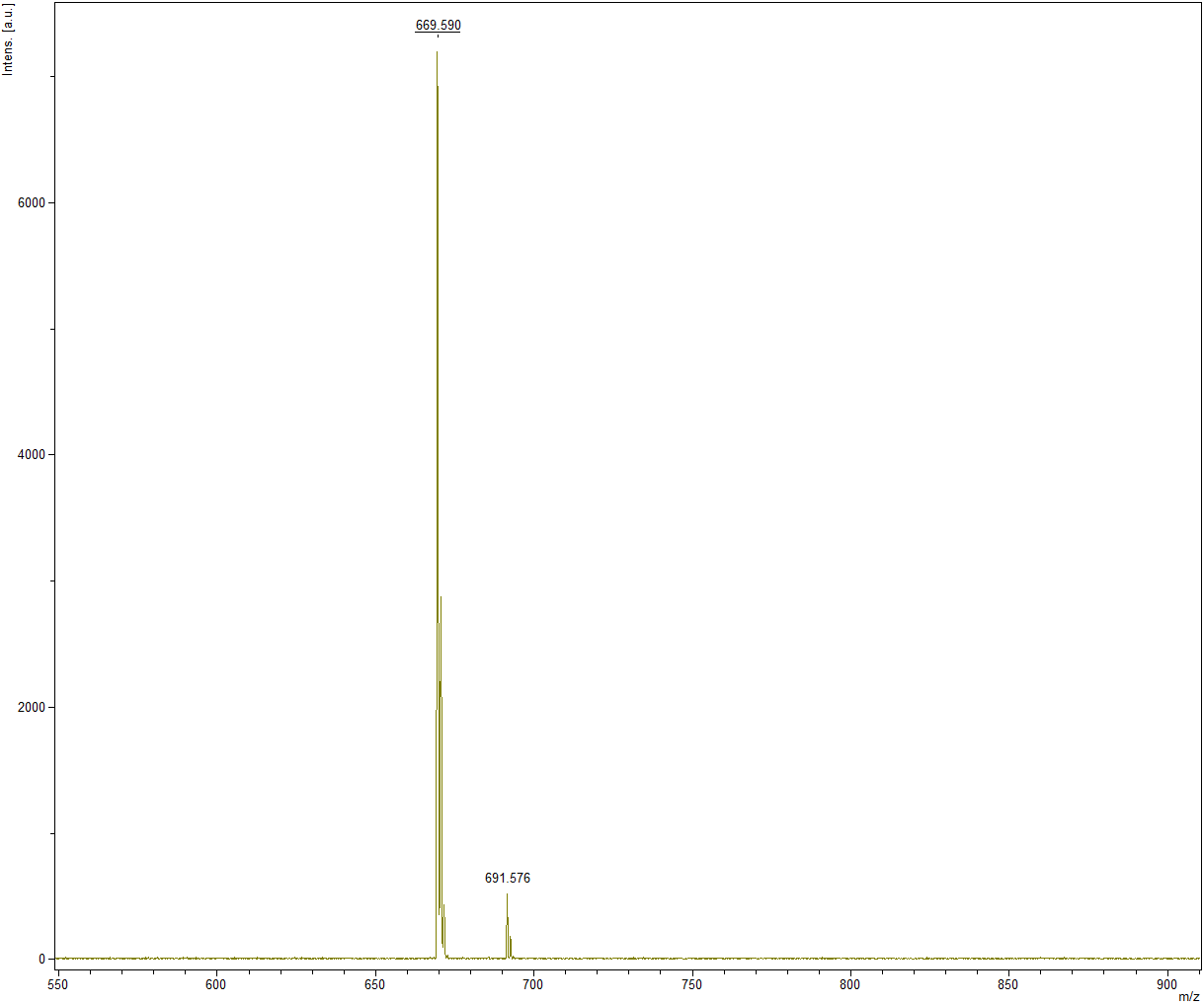


**Figure S44.** Mass spectrum of **A3-3O10**. MALDI-TOF MS m/z of [M + H]^+^ calculated for C_40_H_85_N_4_O_3_: 669.658; Found: 669.590.

**Figure S45.** ^1^H NMR of **A3-4O10**. ^1^H NMR (500 MHz, Chloroform-d) δ 3.68 – 3.58 (m, 4H), 3.19 – 2.17 (m, 24H), 1.93 – 1.54 (m, 4H), 1.50 – 1.24 (m, 56H), 0.88 (t, *J* = 6.9 Hz, 12H).


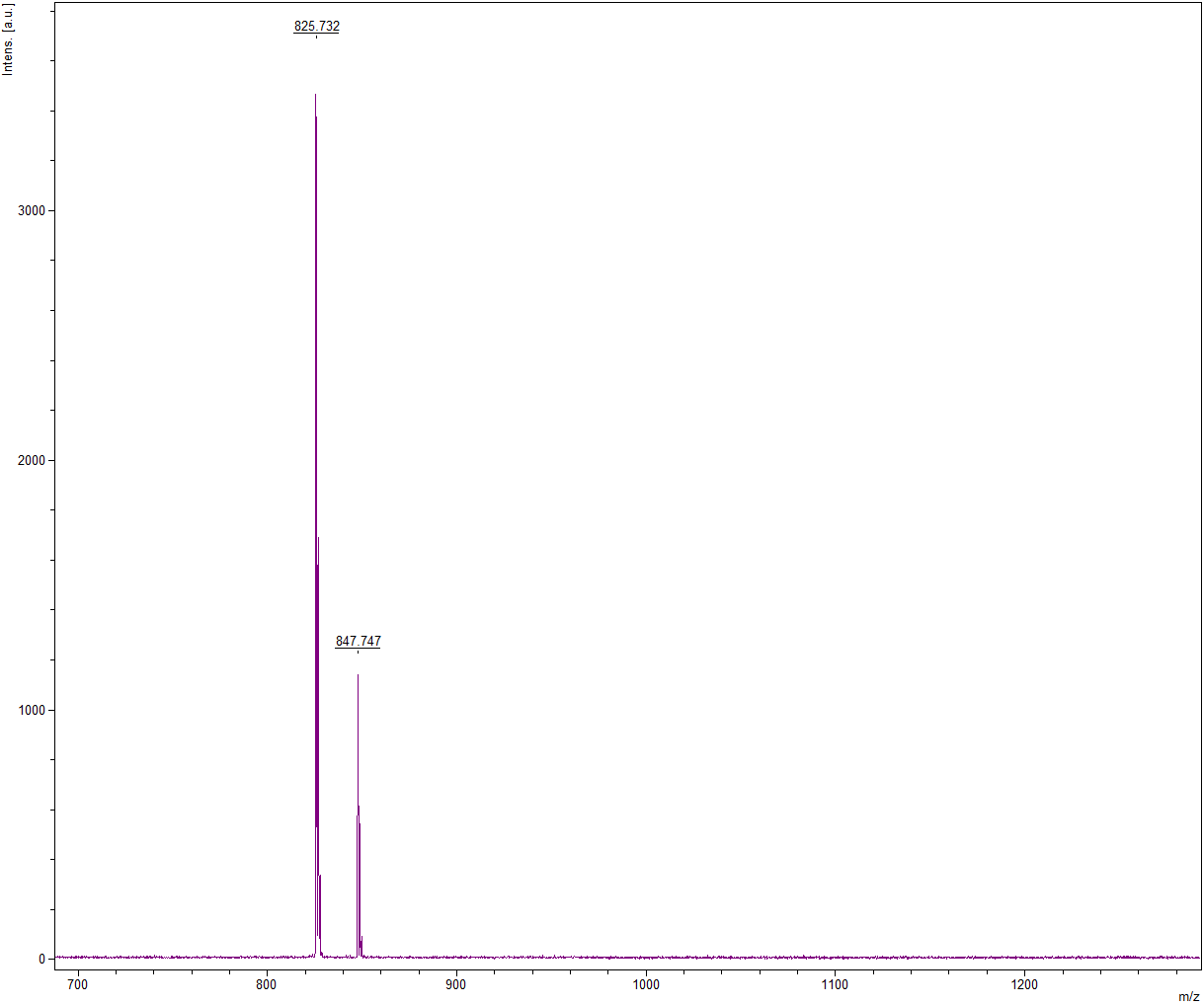


**Figure S46.** Mass spectrum of **A3-4O10**. MALDI-TOF MS m/z of [M + H]^+^ calculated for C_50_H_105_N_4_O_4_: 825.809; Found: 825.732.

**Figure S47.** ^1^H NMR of **A3-3O12**: ^1^H NMR (500 MHz, Chloroform-d) δ 3.67 – 3.56 (m, 3H), 3.02 – 2.12 (m, 22H), 1.72 – 1.58 (m, 4H), 1.51 – 1.25 (m, 54H), 0.88 (t, *J* = 6.9 Hz, 9H).


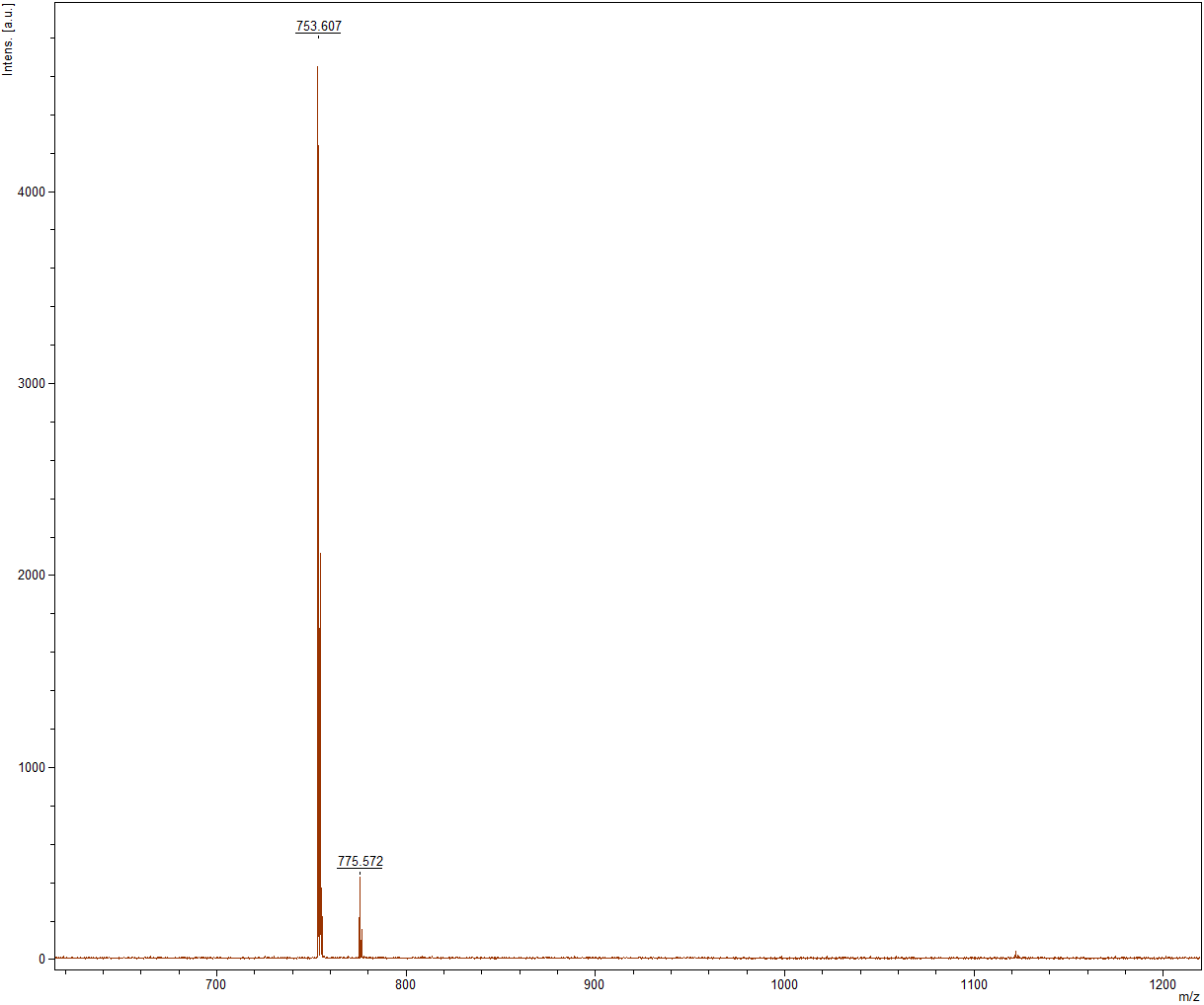


**Figure S48.** Mass spectrum of **A3-3O12.** MALDI-TOF MS m/z of [M + H]^+^ calculated for C_46_H_97_N_4_O_3_: 753.752; Found: 753.607.

**Figure S49.** ^1^H NMR of **A3-4O12**. ^1^H NMR (500 MHz, Chloroform-d) δ 3.78 – 3.57 (m,4H), 3.25 – 2.13 (m, 24H), 1.89 –1.63 (m, 4H), 1.52 – 1.23 (m, 72H), 0.88 (t, *J* = 6.8 Hz, 12H).


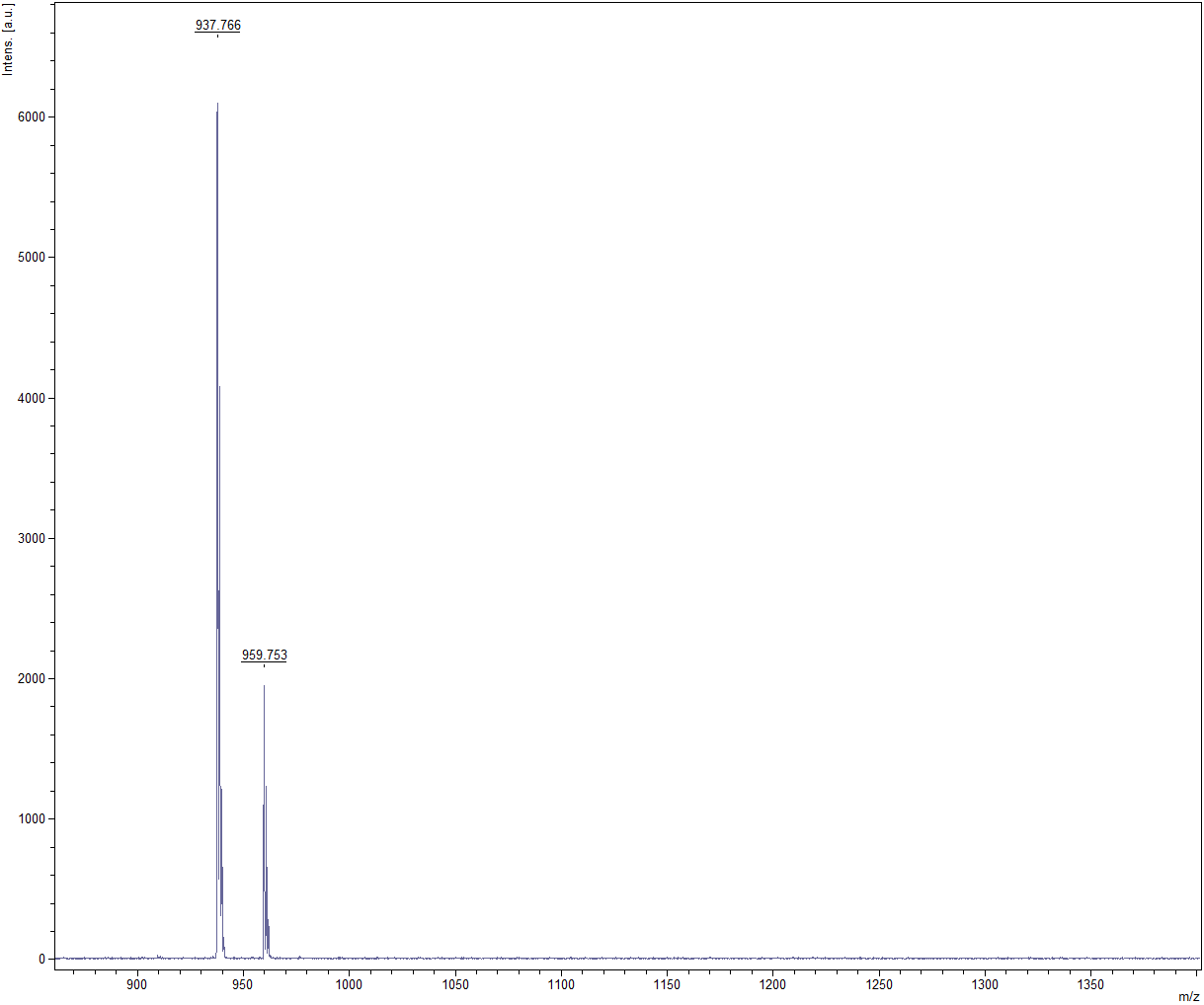


**Figure S50.** Mass spectrum of **A3-4O12**. MALDI-TOF MS m/z of [M + H]^+^ calculated for C_58_H_121_N_4_O_4_: 937.934; Found: 937.766.

**Figure S51.** ^1^H NMR of **A3-3O14**. ^1^H NMR (500 MHz, CDCl_3_) δ 3.75 – 3.51 (m, 3H), 3.02 – 2.19 (m, 22H), 1.85 – 1.52 (m, 4H), 1.49 –1.24 (m, 66H), 0.88 (t, *J* = 6.9 Hz, 9H).


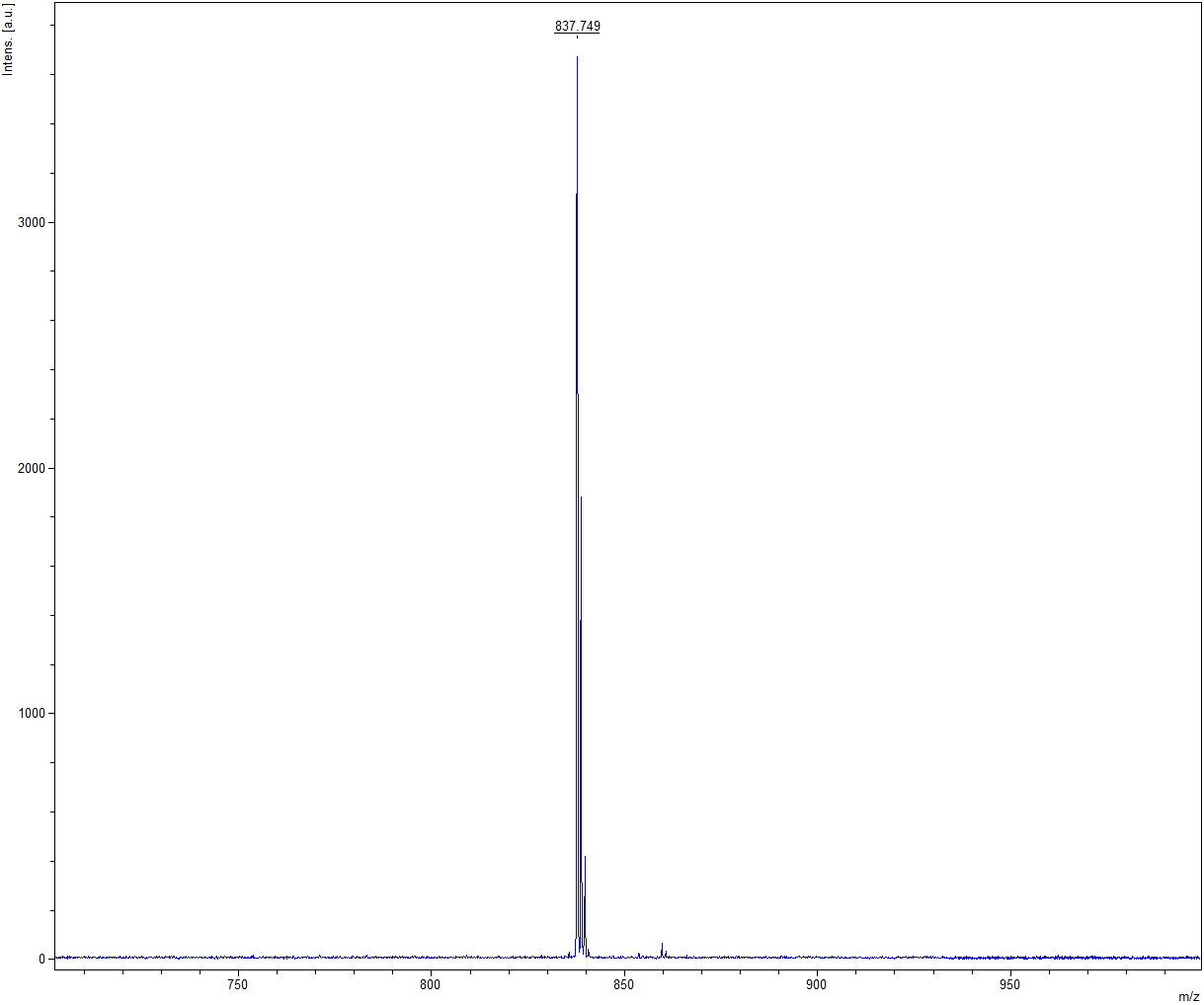


**Figure S52.** Mass spectrum of **A3-3O14**. MALDI-TOF MS m/z of [M + H]^+^ calculated for C_52_H_109_N_4_O_3_: 837.845; Found: 837.749.

**Figure S53.** ^1^H NMR of **A3-4O14**. ^1^H NMR (500 MHz, Chloroform-d) δ 3.72 – 3.56 (m, 4H), 2.99 – 2.10 (m, 24H), 1.79 – 1.54 (m, 4H), 1.50 – 1.05 (m, 88H), 0.88 (t, *J* = 6.9 Hz, 12H).


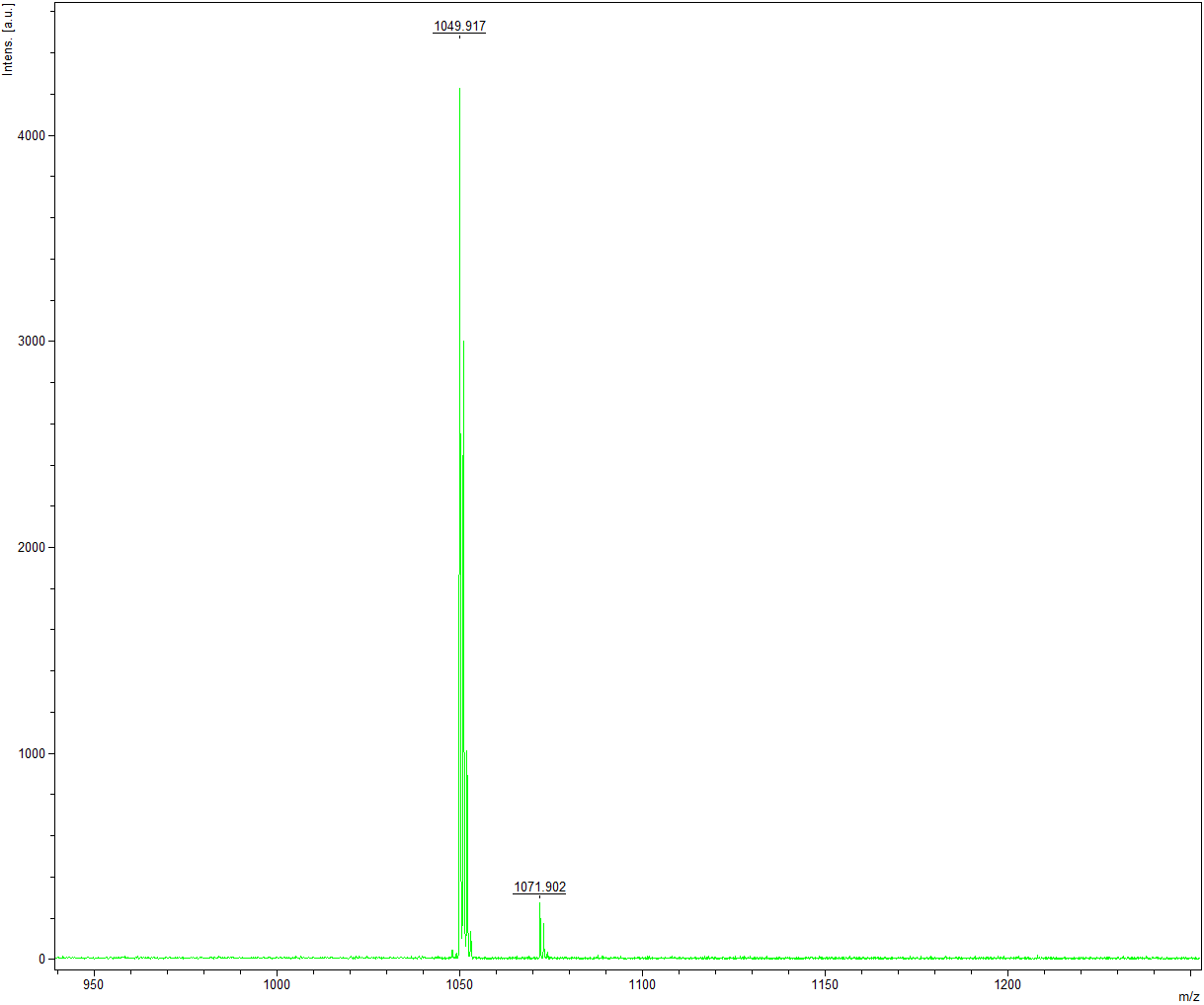


**Figure S54.** Mass spectrum of **A3-4O14**. MALDI-TOF MS m/z of [M + H]^+^ calculated for C_66_H_137_N_4_O_4_: 1050.060; Found: 1049.917.

^^

**Figure S55.** ^1^H NMR of **A3-3O16**. ^1^H NMR (500 MHz, Chloroform-d) δ 3.69 – 3.52 (m, 3H), 2.82 – 2.21 (m, 22H), 1.76 – 1.53 (m, 4H), 1.52 – 1.21 (m, 78H), 0.88 (t, *J* = 6.9 Hz, 9H).


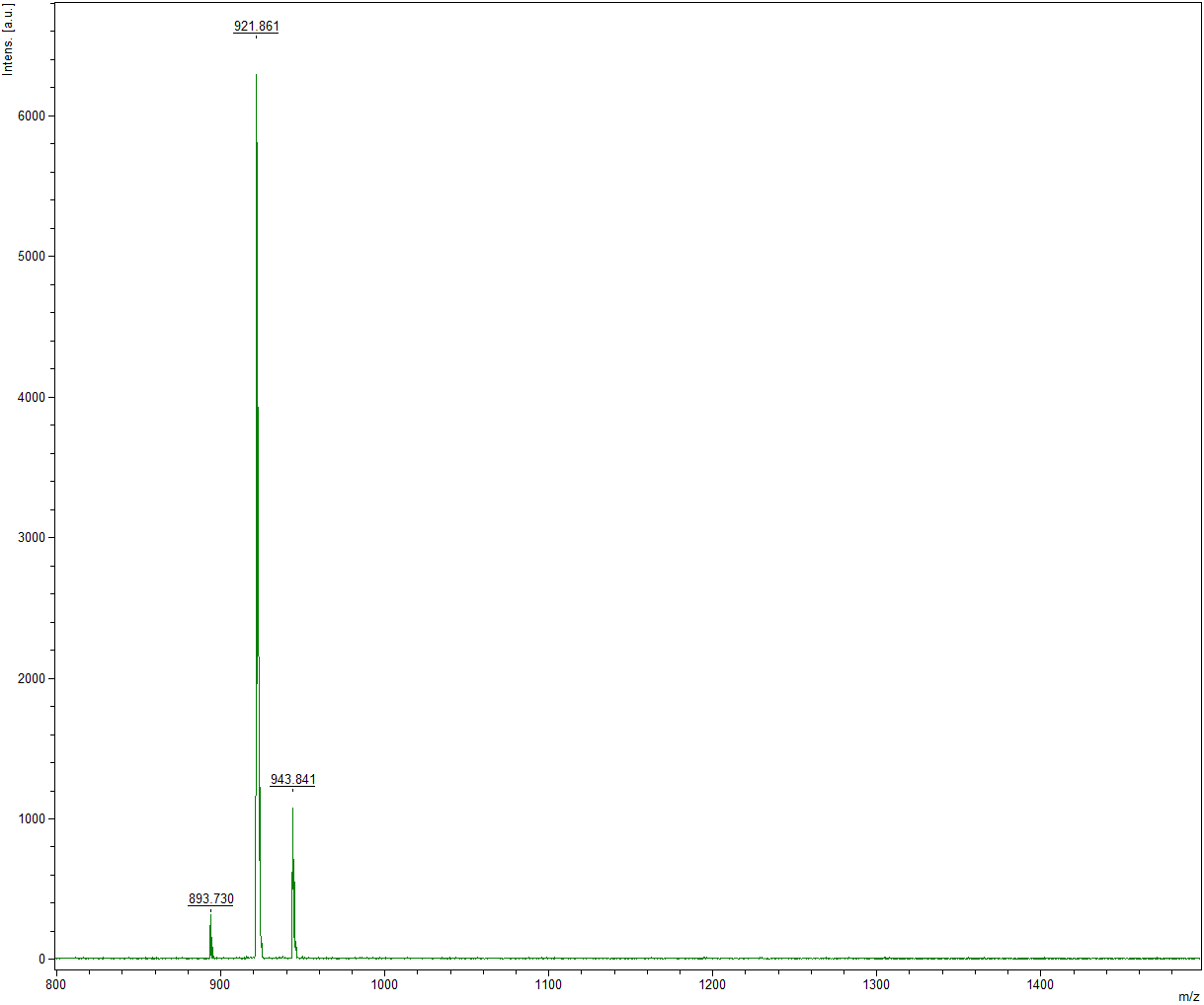


**Figure S56.** Mass spectrum of **A3-3O16**. MALDI-TOF MS m/z of [M + H]^+^ calculated for C_58_H_121_N_4_O_3_: 921.939; Found: 921.861.

**Figure S57.** ^1^H NMR of **A3-4O16**. ^1^H NMR (500 MHz, Chloroform-d) δ 3.82 – 3.64 (m, 4H), 3.27 – 2.34 (m, 24H), 1.84 – 1.70 (m, 4H), 1.52 – 1.12 (m, 104H), 0.88 (t, *J* = 6.8 Hz, 12H).


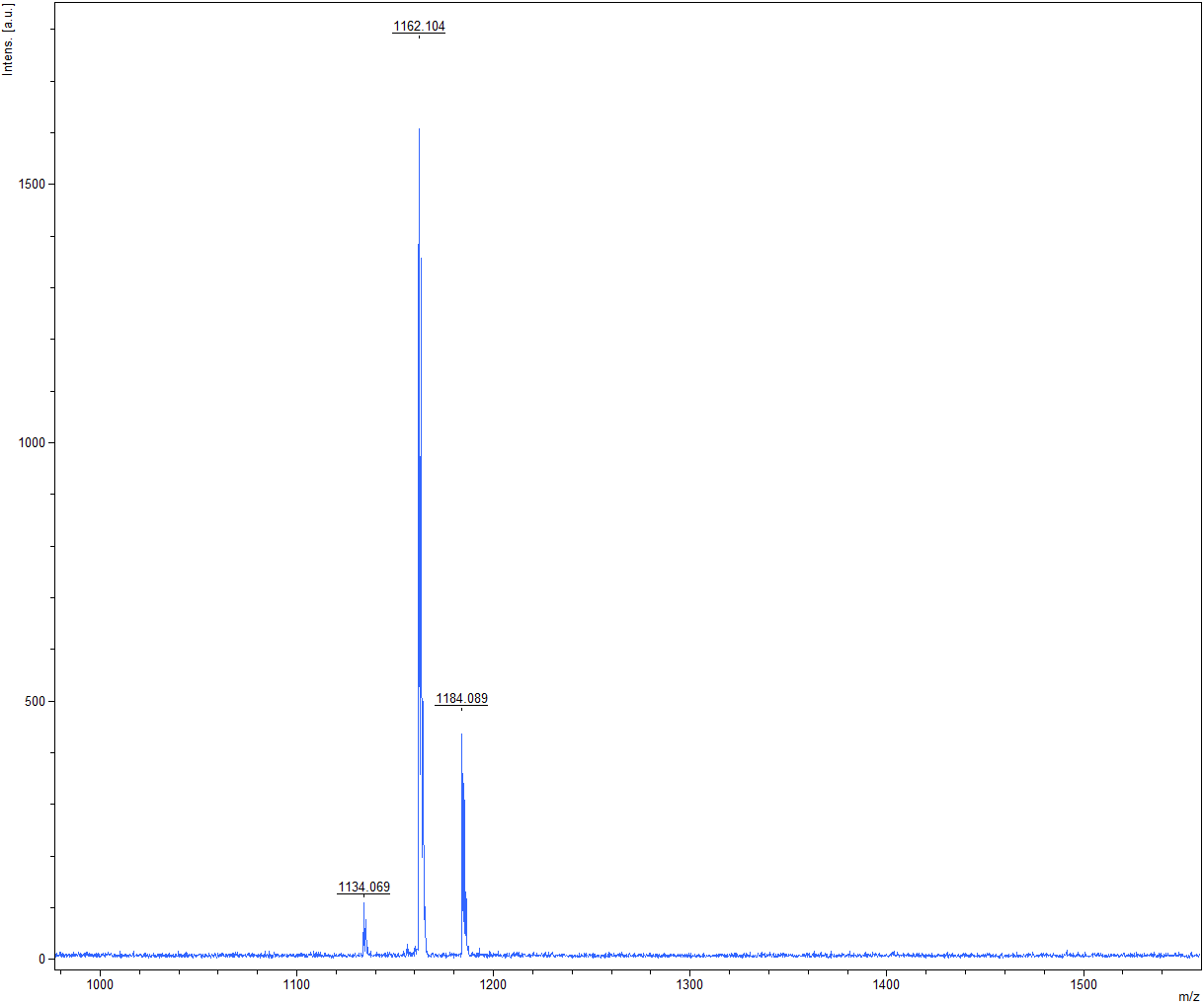


**Figure S58.** Mass spectrum of **A3-4O16**. MALDI-TOF MS m/z of [M + H]^+^ calculated for C_74_H_153_N_4_O_4_: 1162.185; Found: 1162.104.

**Figure S59.** ^1^H NMR of **A3-3O18**. ^1^H NMR (500 MHz, Chloroform-d) δ 3.70 – 3.49 (m, 3H), 2.83 – 2.21 (m, 22H), 1.74 – 1.57 (m, 4H), 1.52 – 1.21 (m, 90H), 0.88 (t, *J* = 6.8 Hz, 9H).


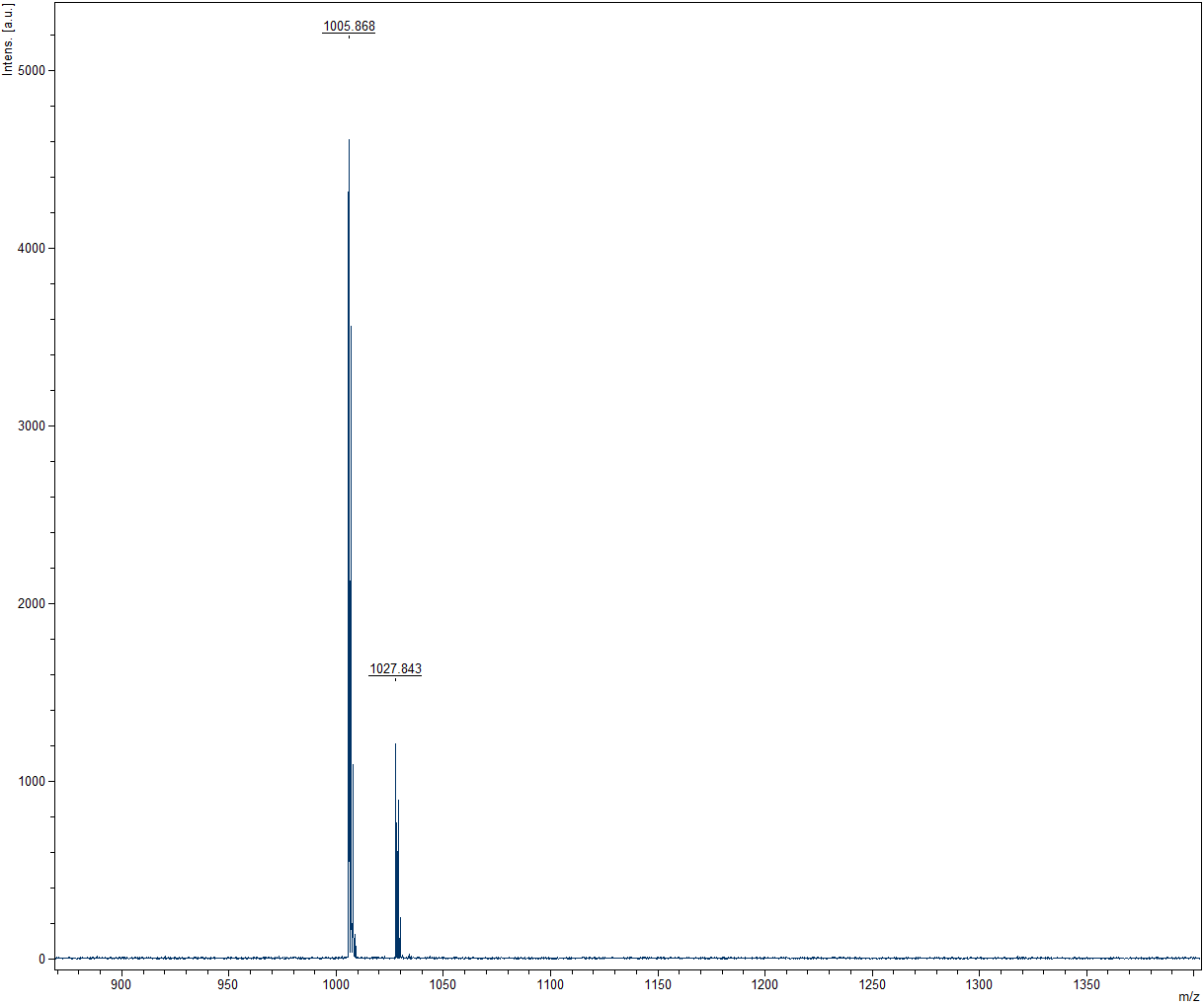


**Figure S60.** Mass spectrum of **A3-3O18**. MALDI-TOF MS m/z of [M + H]^+^ calculated for C_64_H_133_N_4_O_3_: 1006.033; Found: 1005.868.

**Figure S61.** ^1^H NMR of **A3-4O18**. ^1^H NMR (500 MHz, Chloroform-d) δ 3.73 – 3.57 (m, 4H), 3.06 – 2.20 (m, 24H), 1.83 – 1.58 (m, 4H), 1.49 – 1.23 (m, 120H), 0.88 (t, *J* = 6.8 Hz, 12H).


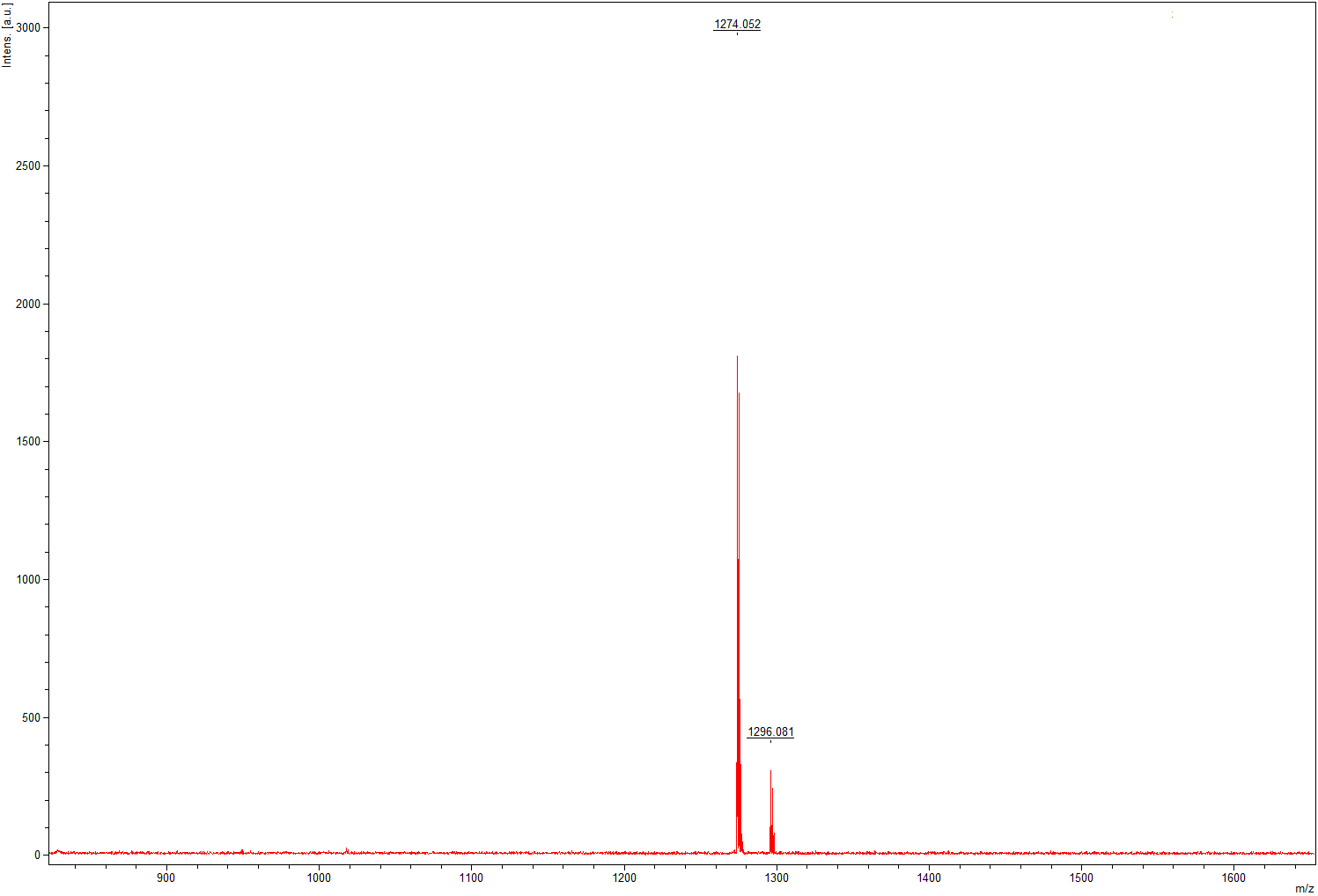


**Figure S62.** Mass spectrum of **A3-4O18**. MALDI-TOF MS m/z of [M + H]^+^ calculated for C_82_H_169_N_4_O_4_: 1274.310; Found: 1274.052.

**Figure S63.** ^1^H NMR of **A5-3O10**. ^1^H NMR (500 MHz, Chloroform-d) δ 3.74 – 3.54 (m, 3H), 2.95 – 2.15 (m, 17H), 1.48 – 1.23 (m, 42H), 0.88 (t, *J* = 7.0 Hz, 9H).


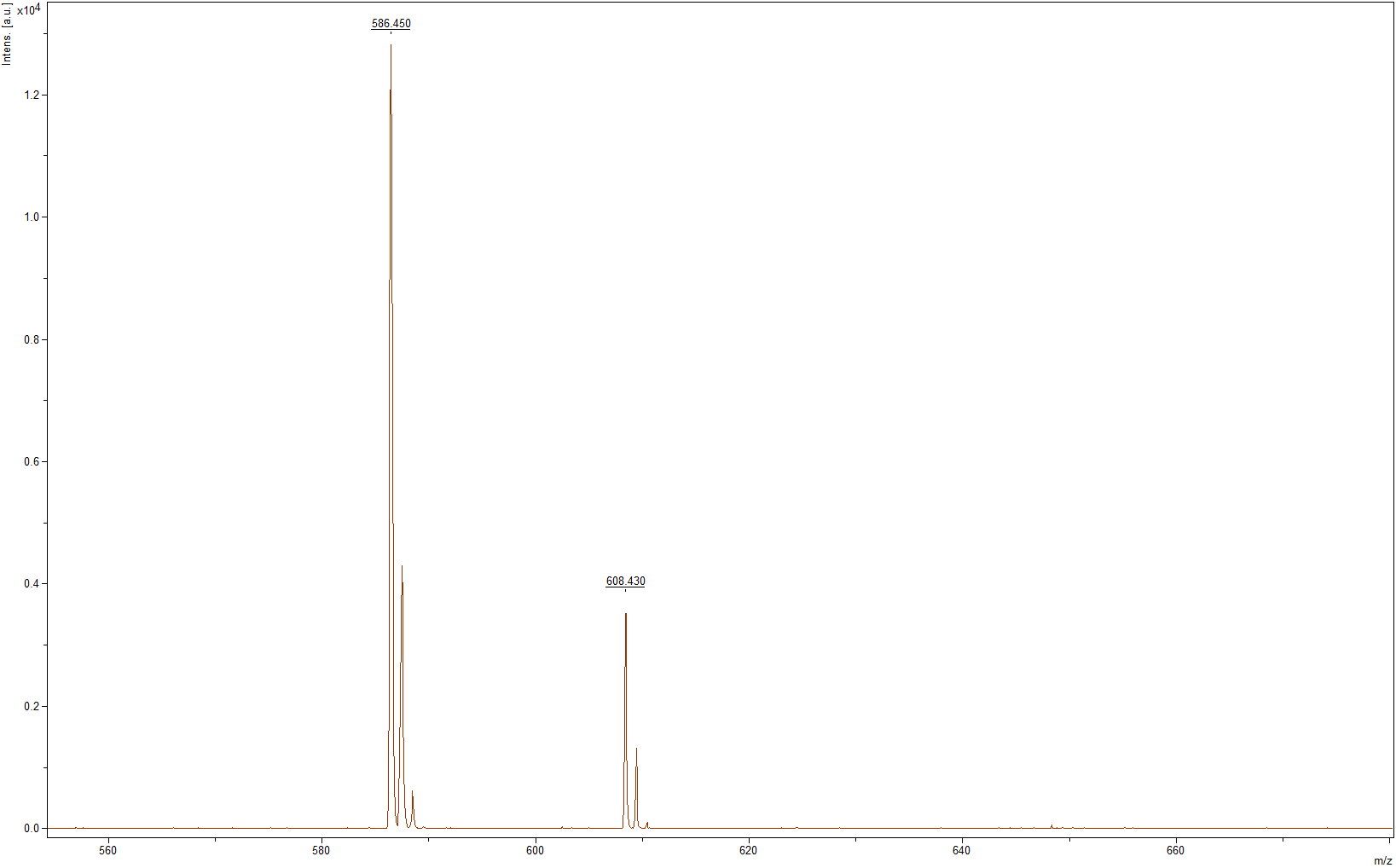


**Figure S64.** Mass spectrum of **A5-3O10**. MALDI-TOF MS m/z of [M + H]^+^ calculated for C_35_H_76_N_3_O_3_: 586.584; Found: 586.450.

**Figure S65.** ^1^H NMR of **A5-4O10**. ^1^H NMR (500 MHz, Chloroform-d) δ 3.85 – 3.45 (m, 4H), 2.88 – 2.03 (m, 19H), 1.50 – 1.23 (m, 56H), 0.87 (t, *J* = 6.9 Hz, 12H).


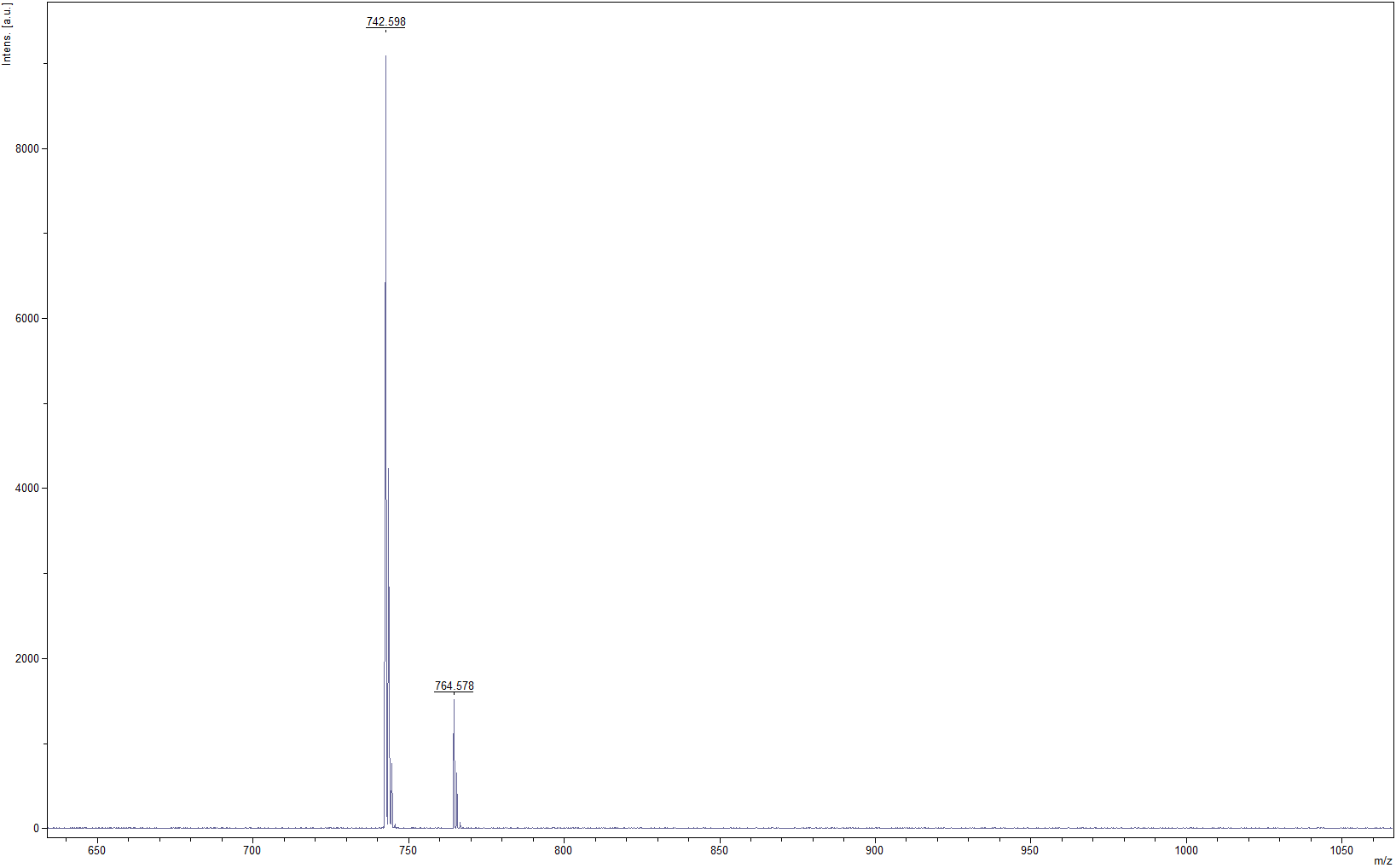


**Figure S66.** Mass spectrum of **A5-4O10**. MALDI-TOF MS m/z of [M + H]^+^ calculated for C_45_H_96_N_3_O_4_: 742.736; Found: 742.598.

**Figure S67.** ^1^H NMR of **A5-3O12**. ^1^H NMR (500 MHz, Chloroform-d) δ 3.70 – 3.55 (m, 3H), 2.89 – 2.17 (m, 17H), 1.52 – 1.16 (m, 54H), 0.88 (t, *J* = 6.8 Hz, 9H).


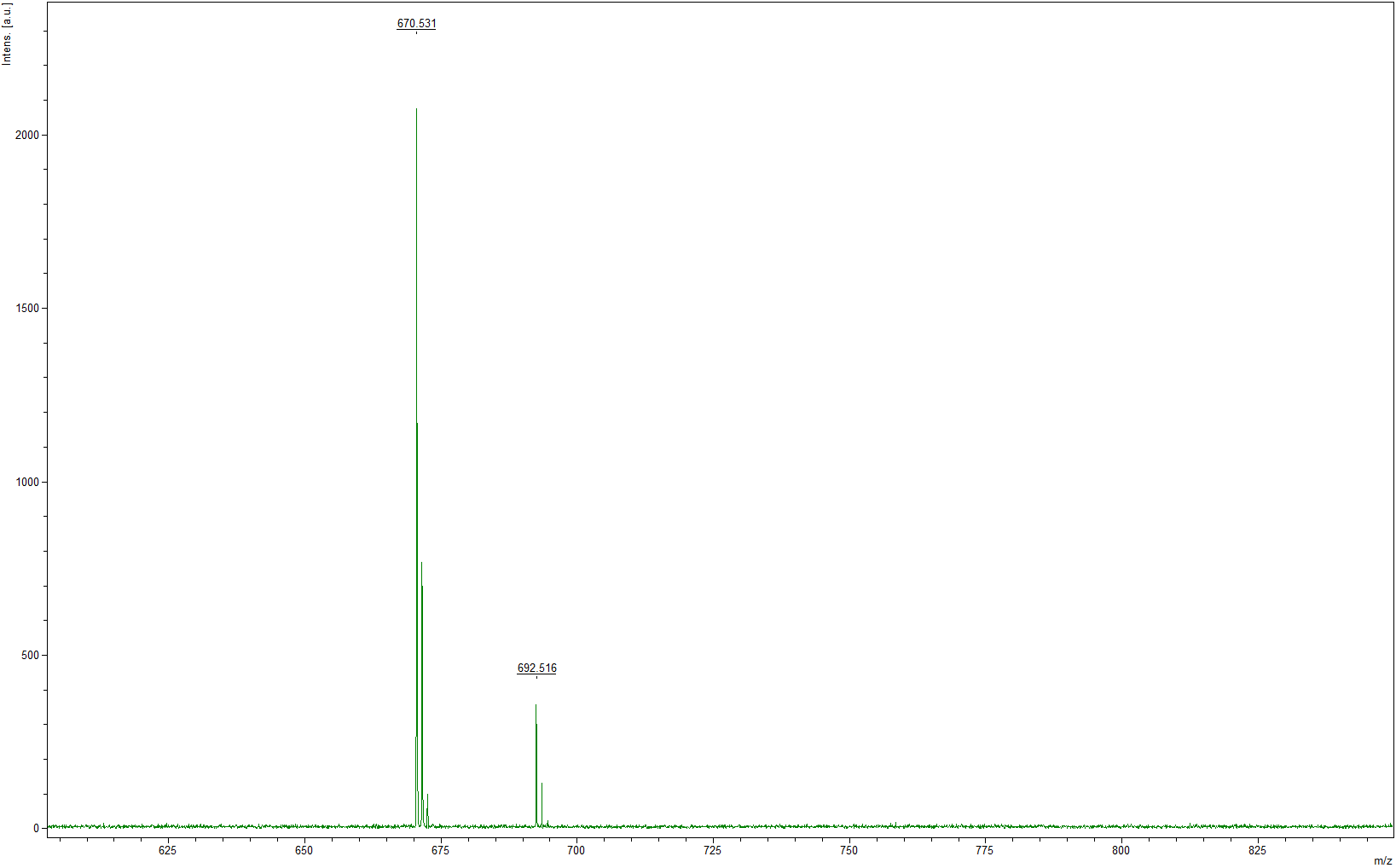


**Figure S68.** Mass spectrum of **A5-3O12**. MALDI-TOF MS m/z of [M + H]^+^ calculated for C_41_H_88_N_3_O_3_: 670.678; Found: 670.531.

**Figure S69.** ^1^H NMR of **A5-4O12**. ^1^H NMR (500 MHz, Chloroform-d) δ 3.64 – 3.54 (m, 4H), 2.98 – 1.99 (m, 19H), 1.81 – 1.08 (m, 72H), 0.88 (t, *J* = 6.9 Hz, 12H).


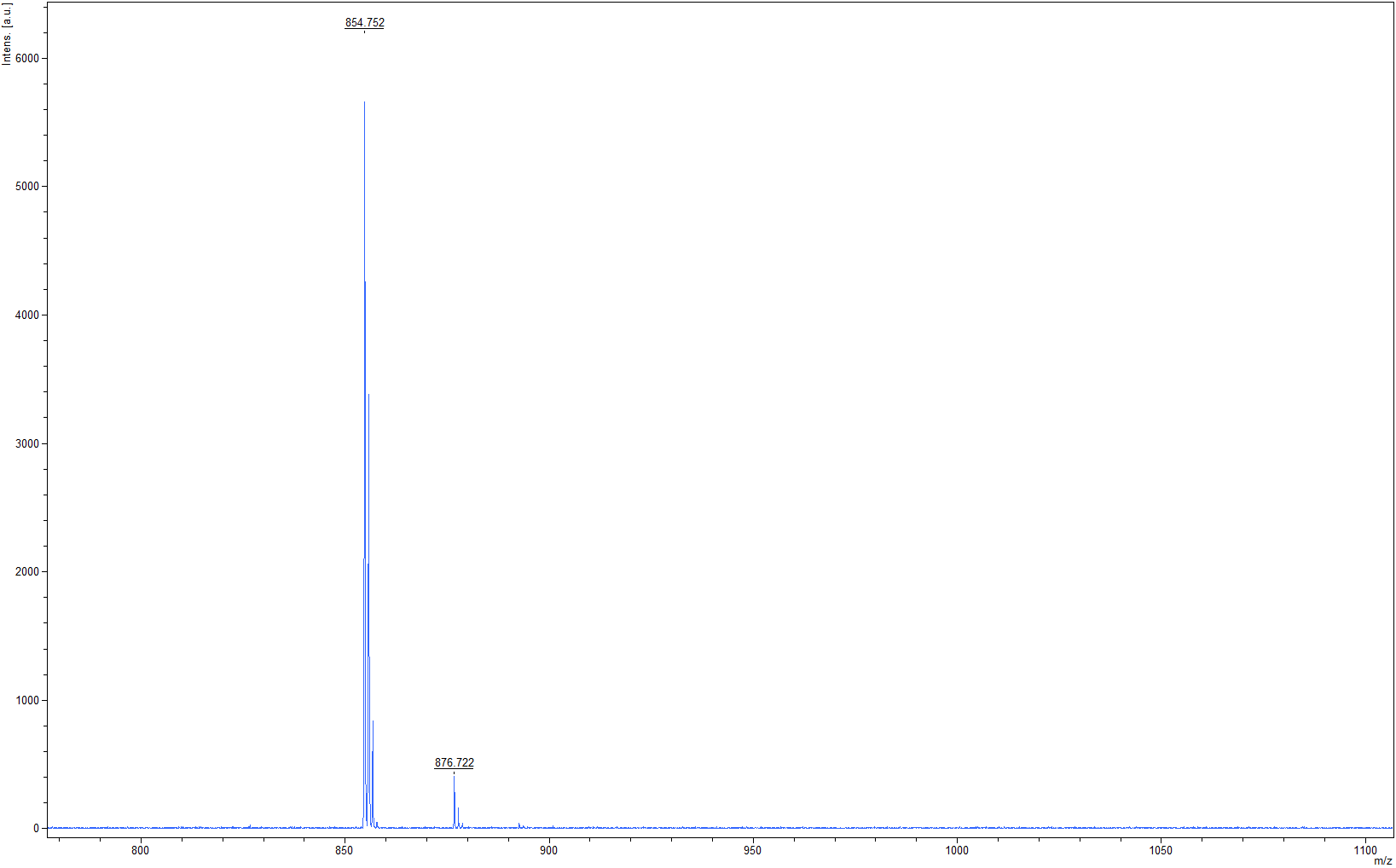


**Figure S70.** Mass spectrum of **A5-4O12**. MALDI-TOF MS m/z of [M + H]^+^ calculated for C_53_H_112_N_3_O_4_: 854.861; Found: 854.752.

**Figure S71.** ^1^H NMR of **A5-3O14**. ^1^H NMR (500 MHz, CDCl_3_) δ 3.70 – 3.53 (m, 3H), 2.91 – 2.16 (m, 17H), 1.51 – 1.04 (m, 66H), 0.88 (t, *J* = 6.9 Hz, 9H).


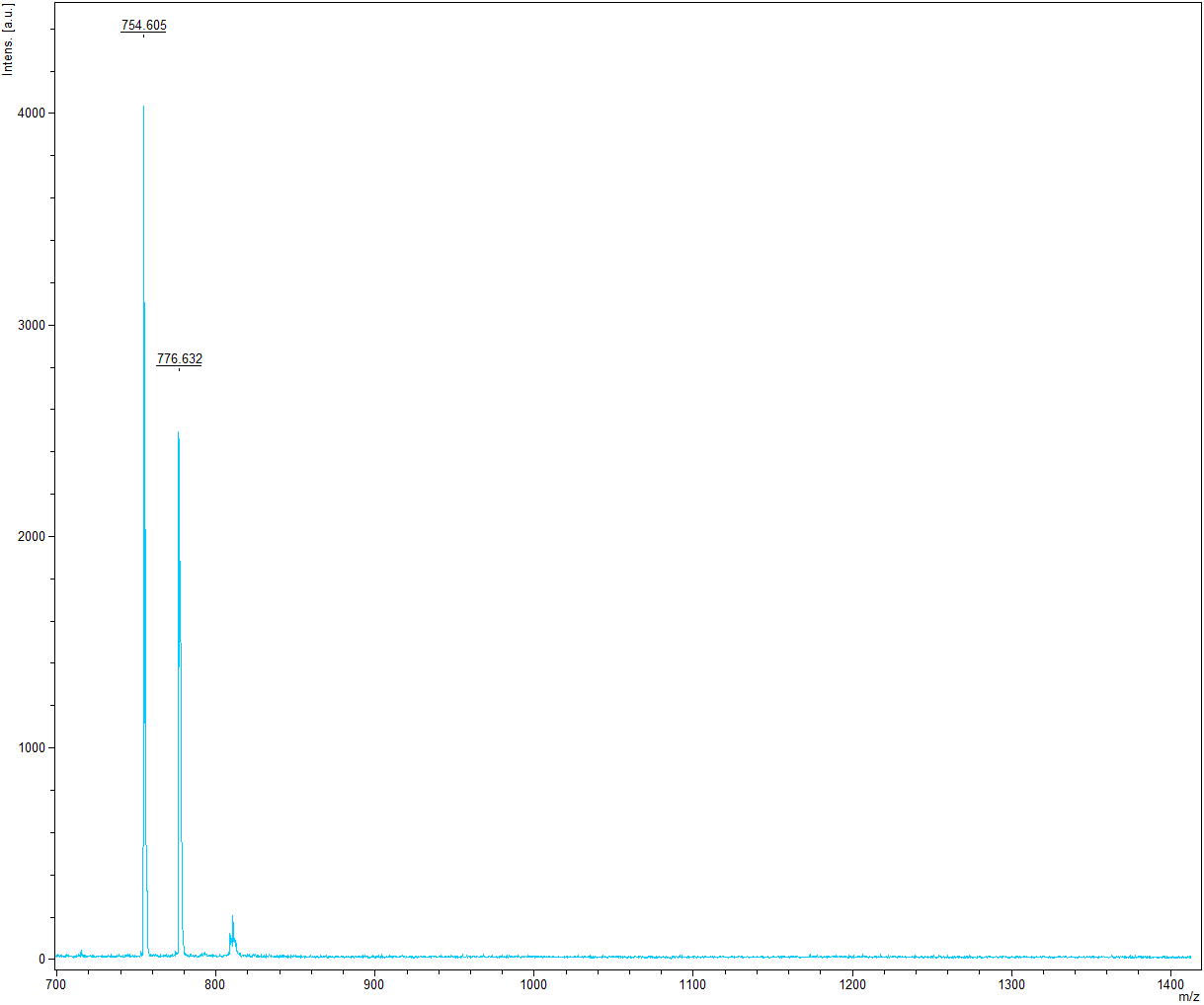


**Figure S72.** Mass spectrum of **A5-3O14**. MALDI-TOF MS m/z of [M + H]^+^ calculated for C_47_H_100_N_3_O_3_: 754.772; Found: 754.605.

**Figure S73.** ^13^C NMR of **A5-3O14**. ^13^C NMR (126 MHz, CDCl_3_) δ70.99, 70.68, 70.21, 68.23, 65.60, 62.89, 56.96, 56.11, 54.97, 54.05, 46.68, 41.92, 34.94, 32.08, 30.05, 29.84, 29.52, 25.93, 22.84, 14.27.

**Figure S74.** ^1^H NMR of **A5-4O14**. ^1^H NMR (500 MHz, Chloroform-d) δ 3.75 – 3.46 (m, 4H), 2.91 – 1.93 (m, 19H), 1.59 – 1.21 (m, 88H), 0.88 (t, *J* = 6.9 Hz, 12H).


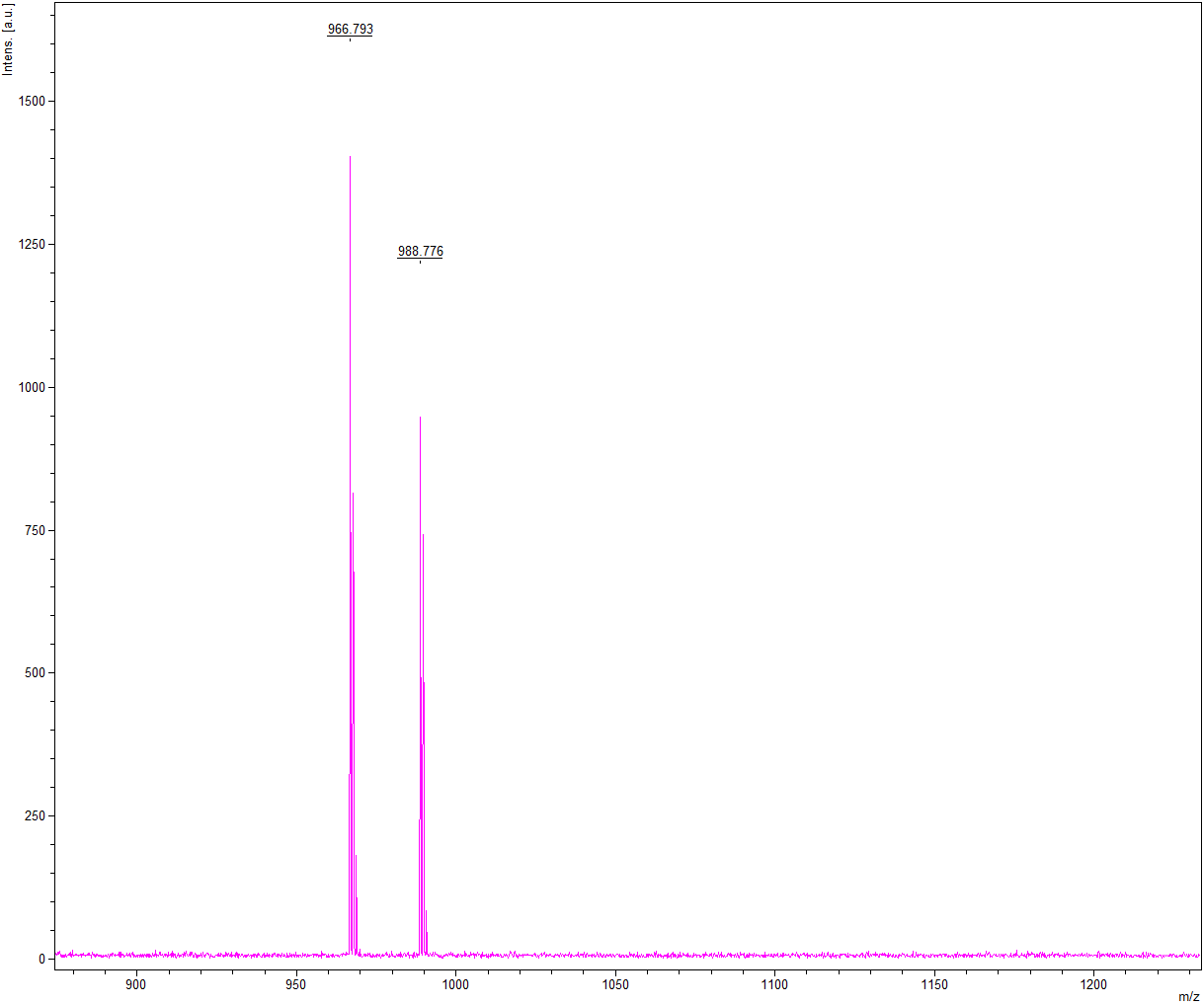


**Figure S75.** Mass spectrum of **A5-4O14**. MALDI-TOF MS m/z of [M + H]^+^ calculated for C_61_H_128_N_3_O_4_: 966.986; Found: 966.793.

**Figure S76.** ^1^H NMR of **A5-3O16**. ^1^H NMR (500 MHz, Chloroform-d) δ 3.69 – 3.54 (m, 3H), 2.90 – 2.20 (m, 17H), 1.45 – 1.20 (m, 78H), 0.88 (t, *J* = 6.9 Hz, 9H).


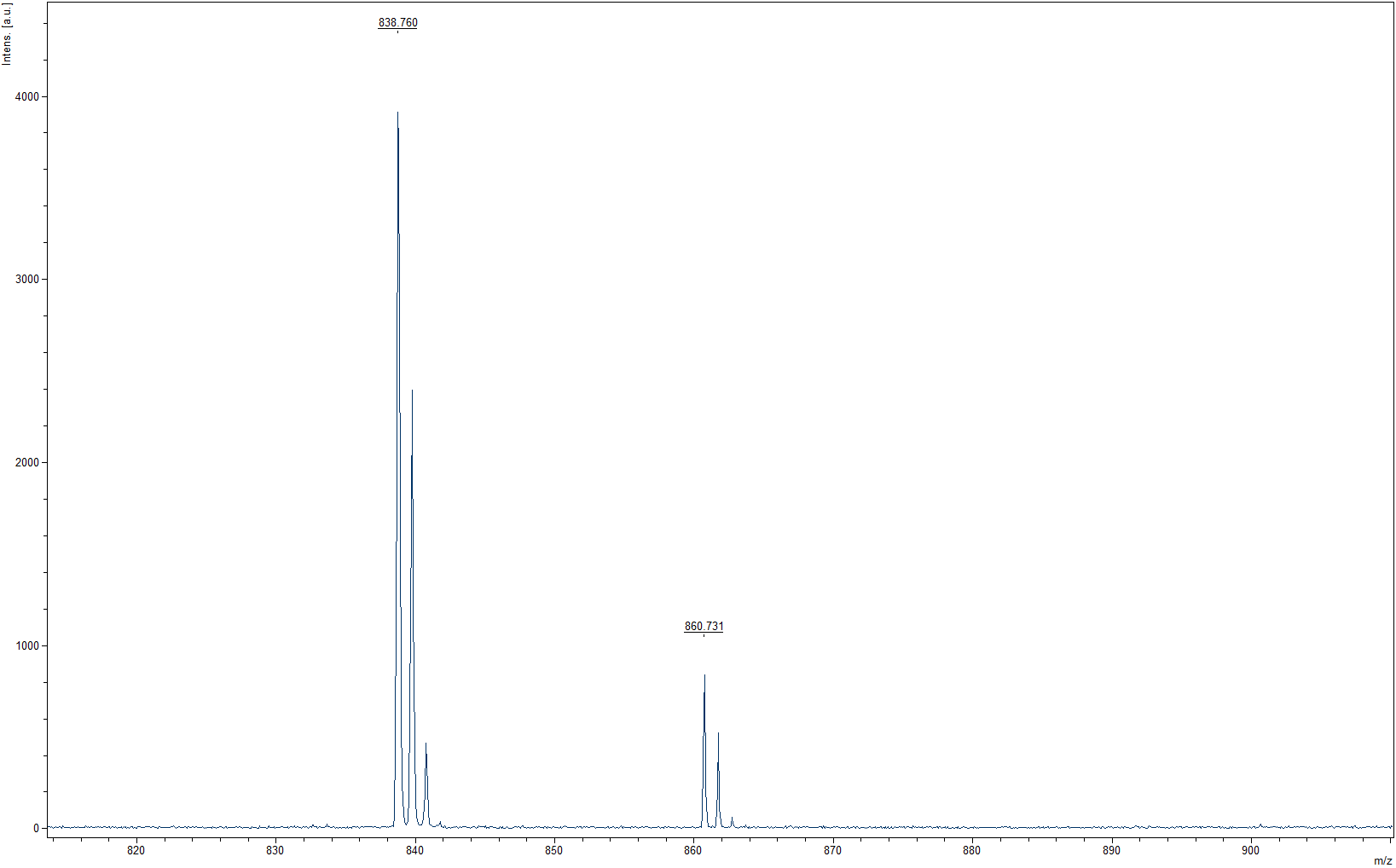


**Figure S77.** Mass spectrum of **A5-3O16**. MALDI-TOF MS m/z of [M + H]^+^ calculated for C_53_H_112_N_3_O_3_: 838.866; Found: 838.760.

**Figure S78.** ^1^H NMR of **A5-4O16**. ^1^H NMR (500 MHz, Chloroform-d) δ 3.68 – 3.46 (m, 4H), 2.93 – 2.05 (m, 19H), 1.58 – 1.11 (m, 104H), 0.88 (t, *J* = 6.9 Hz, 12H).


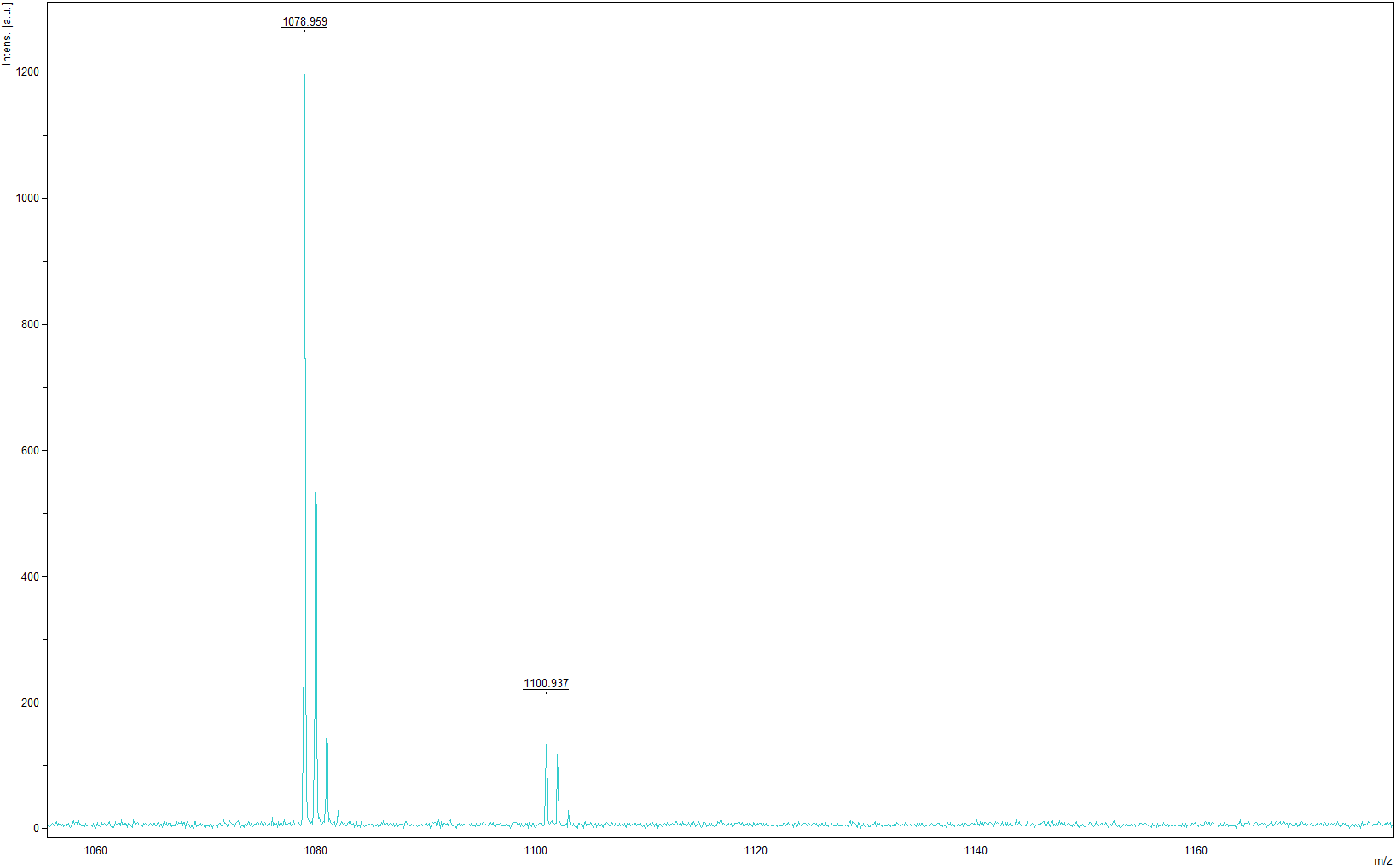


**Figure S79.** Mass spectrum of **A5-4O16**. MALDI-TOF MS m/z of [M + H]^+^ calculated for C_69_H_144_N_3_O_4_: 1079.111; Found: 1078.959.

**Figure S80.** ^1^H NMR of **A5-3O18**. ^1^H NMR (500 MHz, Chloroform-d) δ 3.70 – 3.54 (m, 3H), 2.98 – 2.07 (m, 17H), 1.45 – 1.20 (m, 90H), 0.88 (t, *J* = 6.9 Hz, 9H).


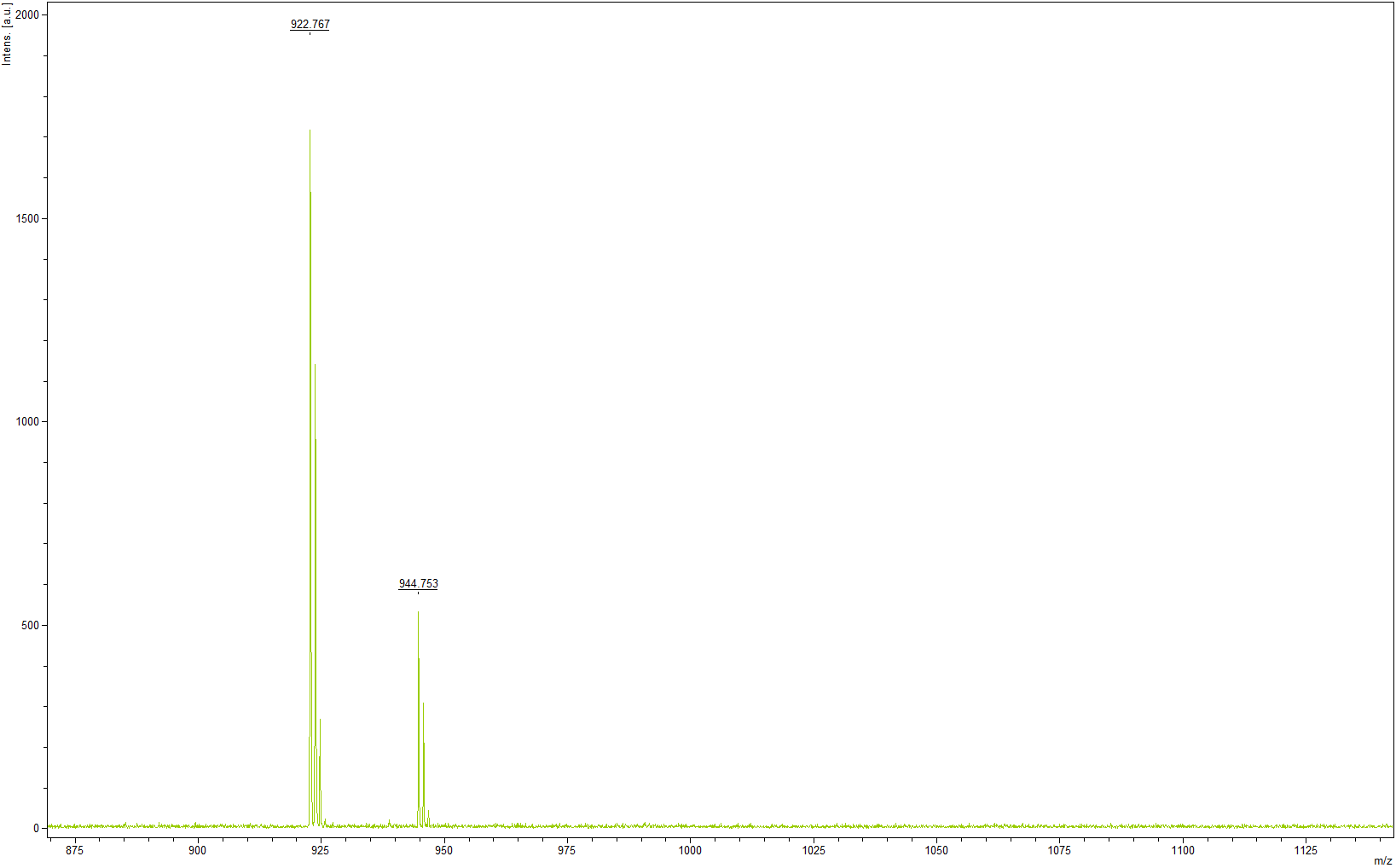


**Figure S81.** Mass spectrum of **A5-3O18**. MALDI-TOF MS m/z of [M + H]^+^ calculated for C_59_H_124_N_3_O_3_: 922.960; Found: 922.767.

**Figure S82.** ^1^H NMR of **A5-4O18**. ^1^H NMR (500 MHz, Chloroform-d) δ 3.70 – 3.52 (m, 4H), 3.06 – 2.08 (m, 19H), 1.62 – 1.05 (m, 120H), 0.88 (t, *J* = 6.9 Hz, 12H).


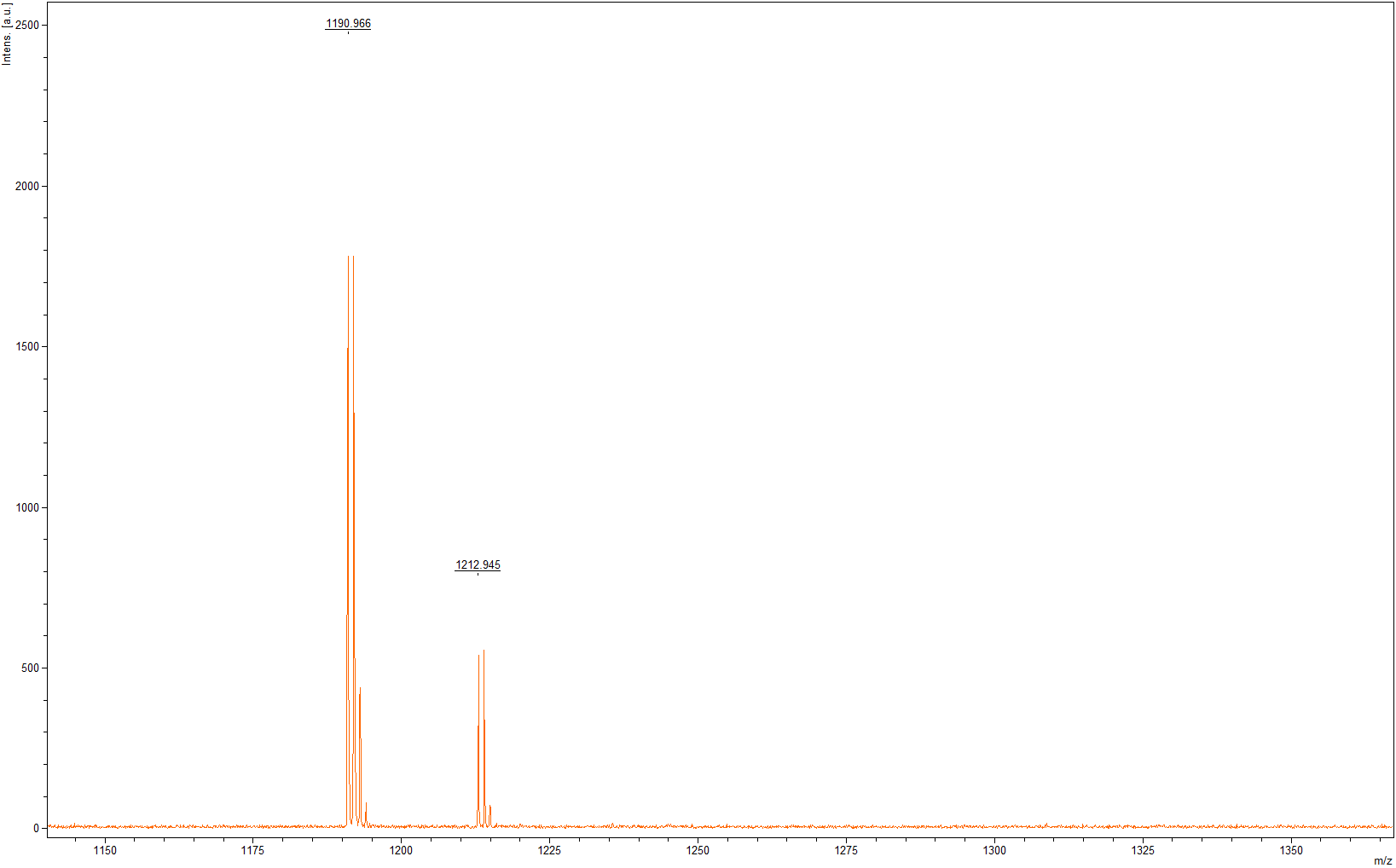


**Figure S83.** Mass spectrum of **A5-4O18**. MALDI-TOF MS m/z of [M + H]^+^ calculated for C_77_H_160_N_3_O_4_: 1191.236; Found: 1190.966.

**Figure S84.** ^1^H NMR of **A6-3O10**. ^1^H NMR (500 MHz, Chloroform-d) δ 3.71 – 3.57 (m, 3H), 2.98 – 2.16 (m, 10H), 1.83 – 1.52 (m, 2H), 1.50 – 1.22 (m, 42H), 0.88 (t, *J* = 6.9 Hz, 9H).


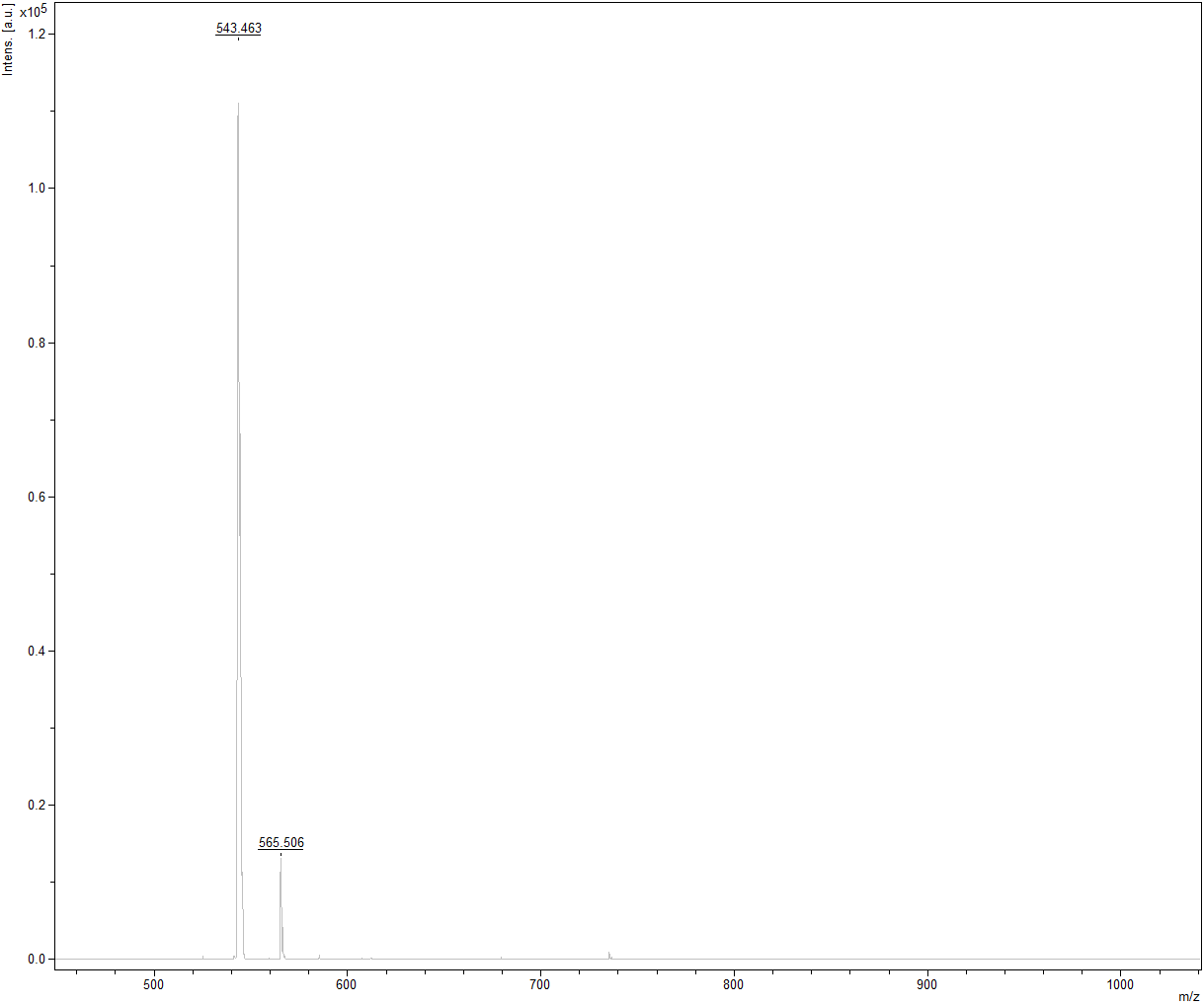


**Figure S85.** Mass spectrum of **A6-3O10**. MALDI-TOF MS m/z of [M + H]^+^ calculated for C_33_H_71_N_2_O_3_: 543.542; Found: 543.463.

**Figure S86.** ^1^H NMR of **A6-4O10**. ^1^H NMR (500 MHz, Chloroform-d) δ 3.78 – 3.62 (m, 4H), 2.85 – 2.10 (m, 12H), 1.73 – 1.60 (m, 2H), 1.56 – 0.97 (m, 56H), 0.88 (t, *J* = 6.9 Hz, 12H).


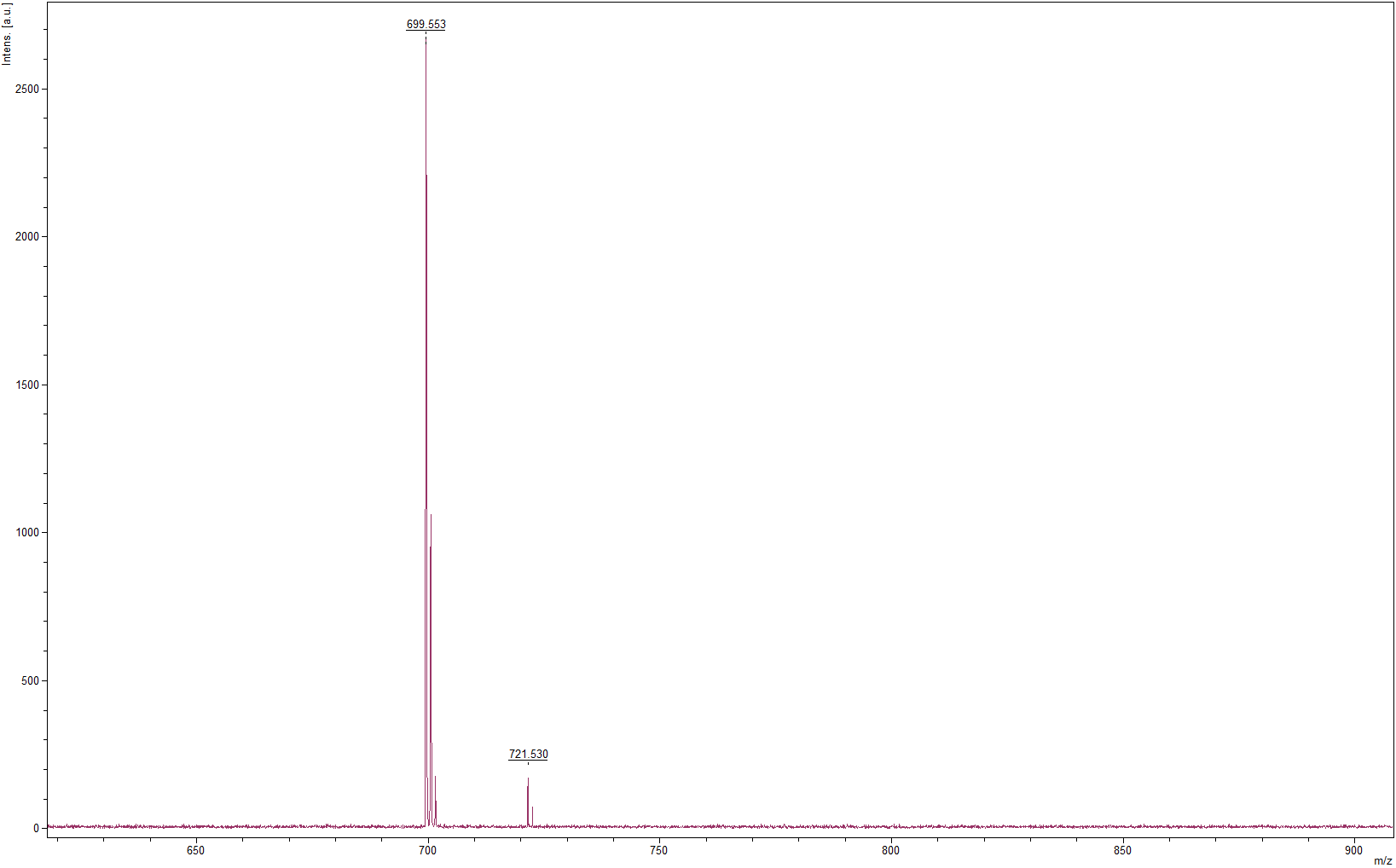


**Figure S87.** Mass spectrum of **A6-4O10**. MALDI-TOF MS m/z of [M + H]^+^ calculated for C_43_H_91_N_2_O_4_: 699.693; Found: 699.553.

**Figure S88.** ^1^H NMR of **A6-3O12**. ^1^H NMR (500 MHz, Chloroform-d) δ 3.73 – 3.56 (m, 3H), 3.02 – 2.16 (m, 10H), 1.72 – 1.54 (m, 2H), 1.48 – 1.23 (m, 54H), 0.88 (t, *J* = 6.8 Hz, 9H).


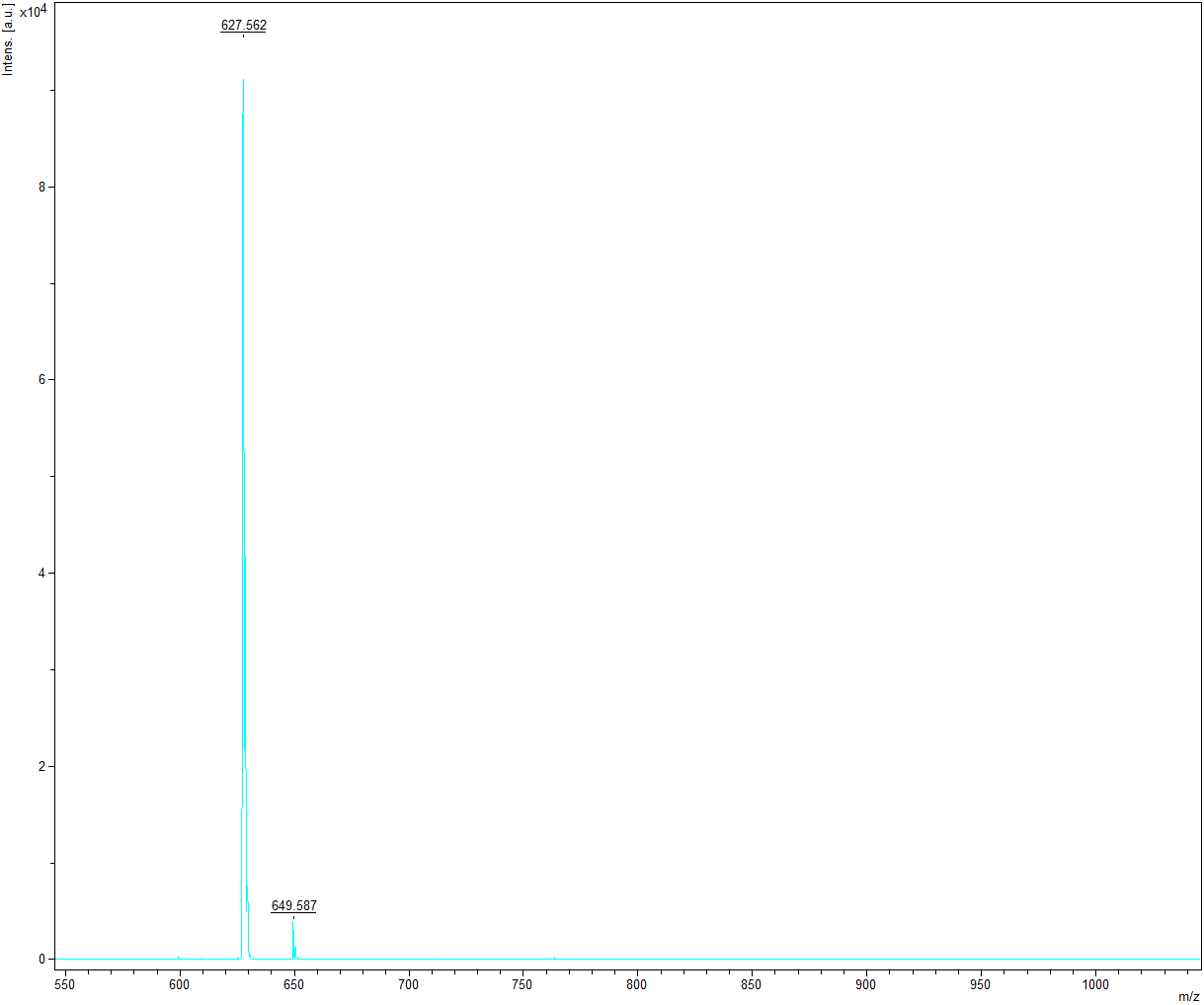


**Figure S89.** Mass spectrum of **A6-3O12**. MALDI-TOF MS m/z of [M + H]^+^ calculated for C_39_H_83_N_2_O_3_: 627.636; Found: 627.562.

**Figure S90.** ^1^H NMR of **A6-4O12**. ^1^H NMR (500 MHz, Chloroform-d) δ 3.79 – 3.57 (m, 4H), 3.01 – 2.07 (m, 12H), 1.85 – 1.56 (m, 2H), 1.52 – 1.02 (m, 72H), 0.88 (t, *J* = 6.8 Hz, 12H).


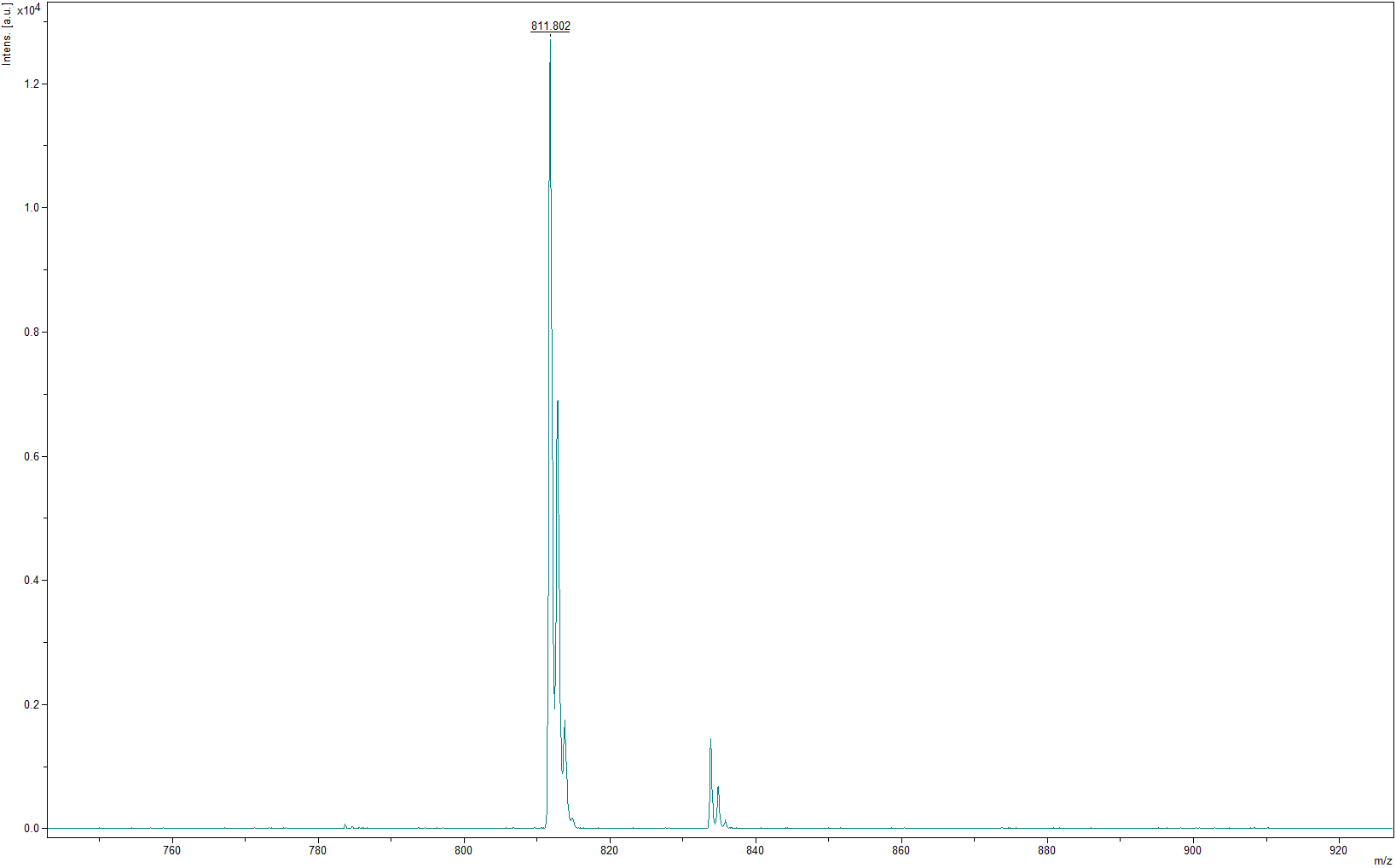


**Figure S91.** Mass spectrum of **A6-4O12**. MALDI-TOF MS m/z of [M + H]^+^ calculated for C_51_H_107_N_2_O_4_: 811.819; Found: 811.802.

**Figure S92.** ^1^H NMR of **A6-3O14**. ^1^H NMR (500 MHz, Chloroform-d) δ 3.83 – 3.54 (m, 3H), 3.10 – 2.17 (m, 10H), 1.85 – 1.53 (m, 2H), 1.51 – 1.03 (m, 66H), 0.88 (t, *J* = 6.9 Hz, 9H).


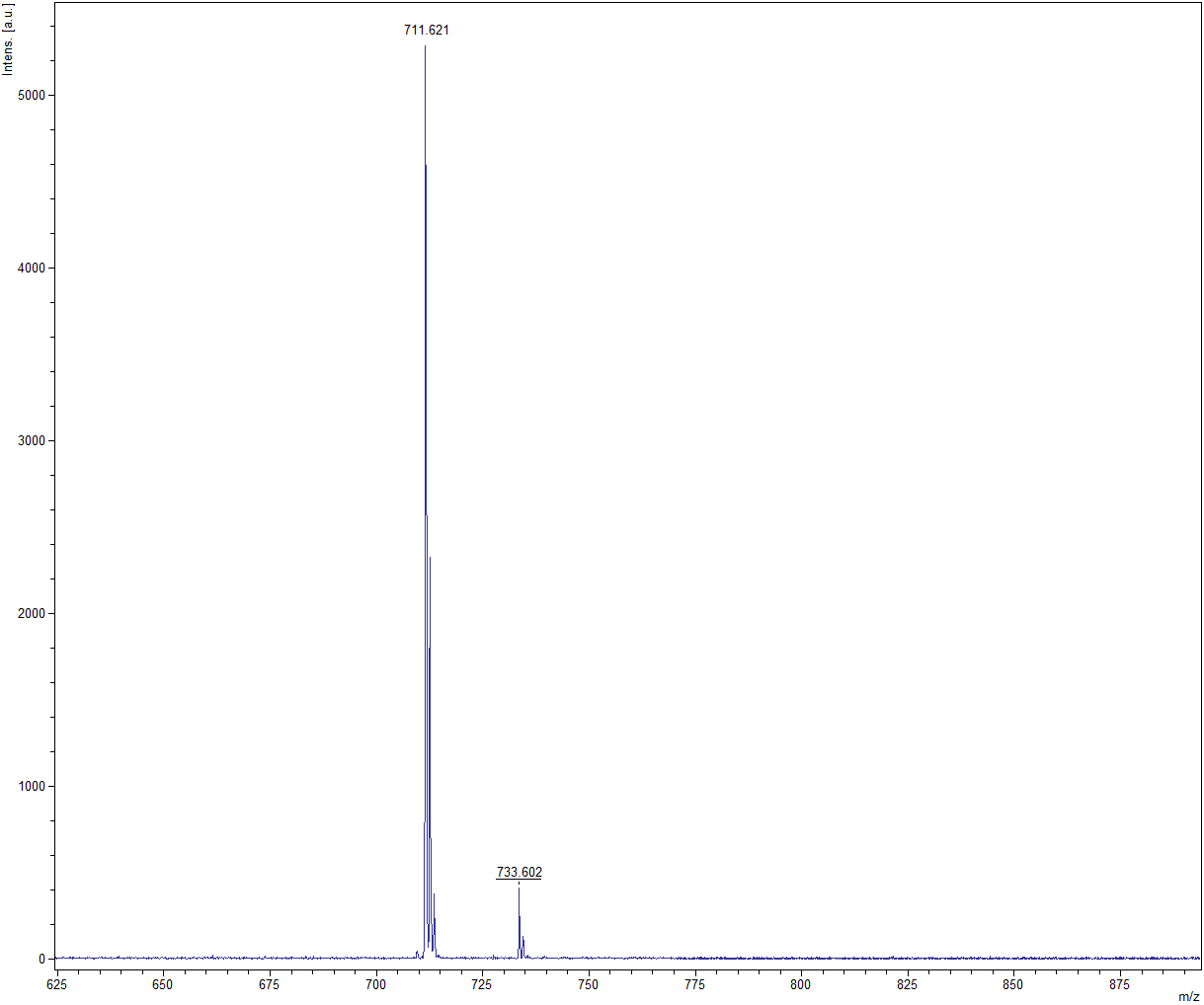


**Figure S93.** Mass spectrum of **A6-3O14**. MALDI-TOF MS m/z of [M + H]^+^ calculated for C_45_H_95_N_2_O_3_: 711.730; Found: 711.621.

**Figure S94.** ^1^H NMR of **A6-4O14**. ^1^H NMR (500 MHz, Chloroform-d) δ 3.76 – 3.61 (m, 4H), 2.85 – 2.11 (m, 12H), 1.72 – 1.62 (m, 2H), 1.52 – 1.19 (m, 88H), 0.88 (t, *J* = 6.9 Hz, 12H).


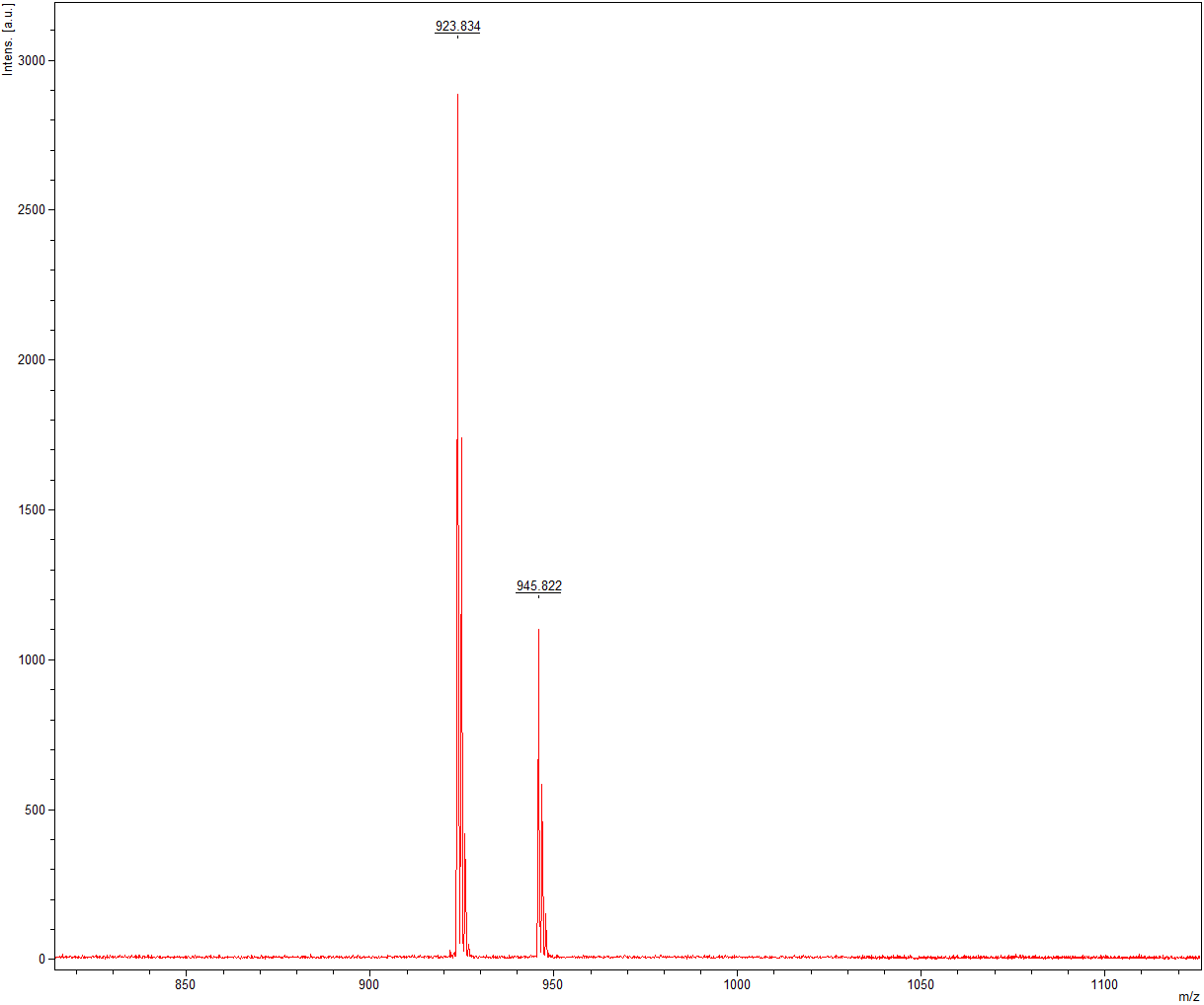


**Figure S95.** Mass spectrum of **A6-4O14**. MALDI-TOF MS m/z of [M + H]^+^ calculated for C_59_H_123_N_2_O_4_: 923.944; Found: 923.834.

**Figure S96.** ^1^H NMR of **A6-3O16**. ^1^H NMR (500 MHz, Chloroform-d) δ 3.75 – 3.58 (m, 3H), 3.08 – 2.17 (m, 10H), 1.81 – 1.54 (m, 2H), 1.51 – 1.12 (m, 78H), 0.88 (t, *J* = 6.9 Hz, 9H).


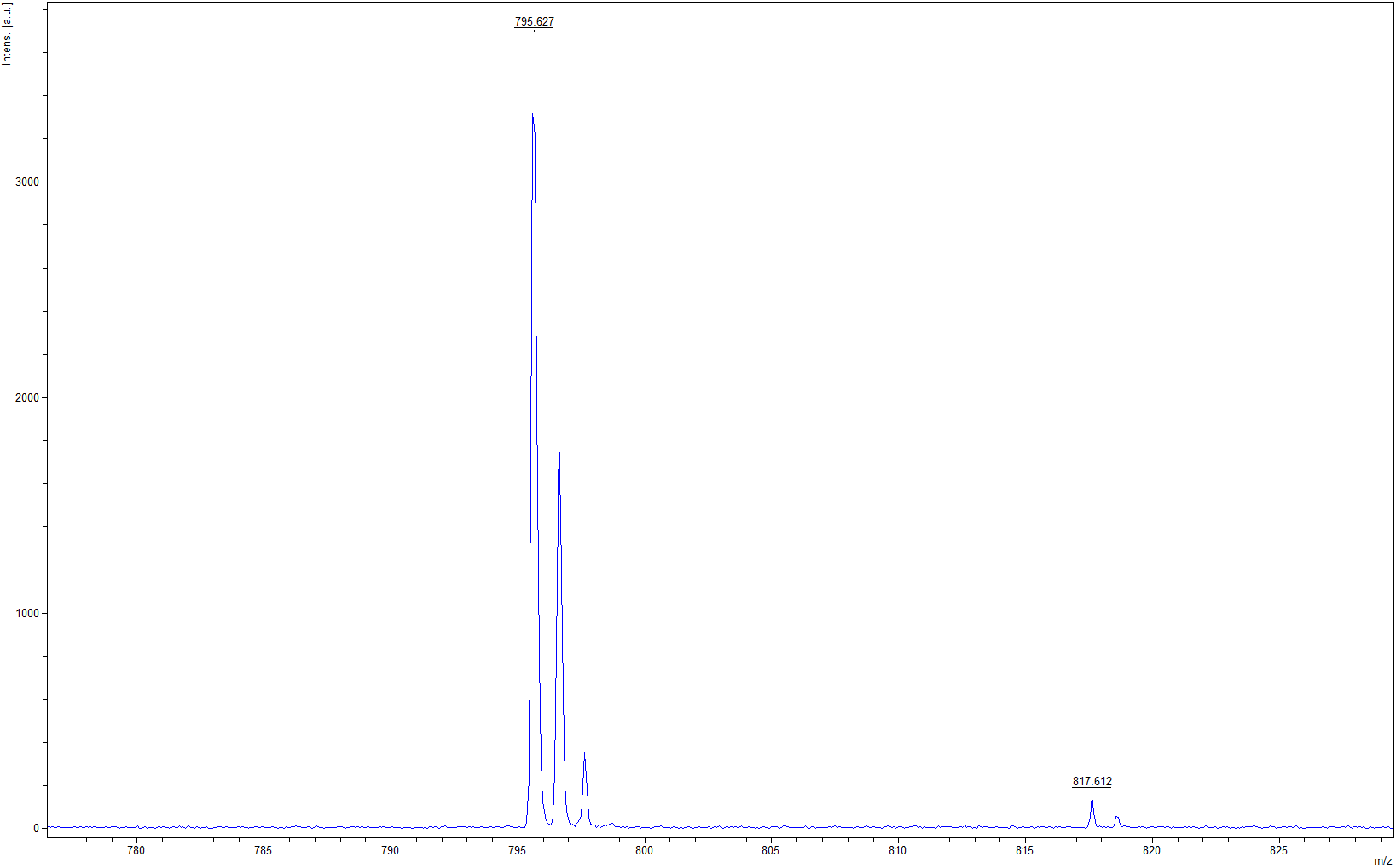


**Figure S97.** Mass spectrum of **A6-3O16**. MALDI-TOF MS m/z of [M + H]^+^ calculated for C_51_H_107_N_2_O_3_: 795.824; Found: 795.627.

**Figure S98.** ^1^H NMR of **A6-4O16**. ^1^H NMR (500 MHz, Chloroform-d) δ 3.81 – 3.58 (m, 4H), 2.94 – 2.11 (m, 12H), 1.64 – 1.84 (m, 2H), 1.53 – 1.10 (m, 104H), 0.88 (t, *J* = 6.9 Hz, 12H).


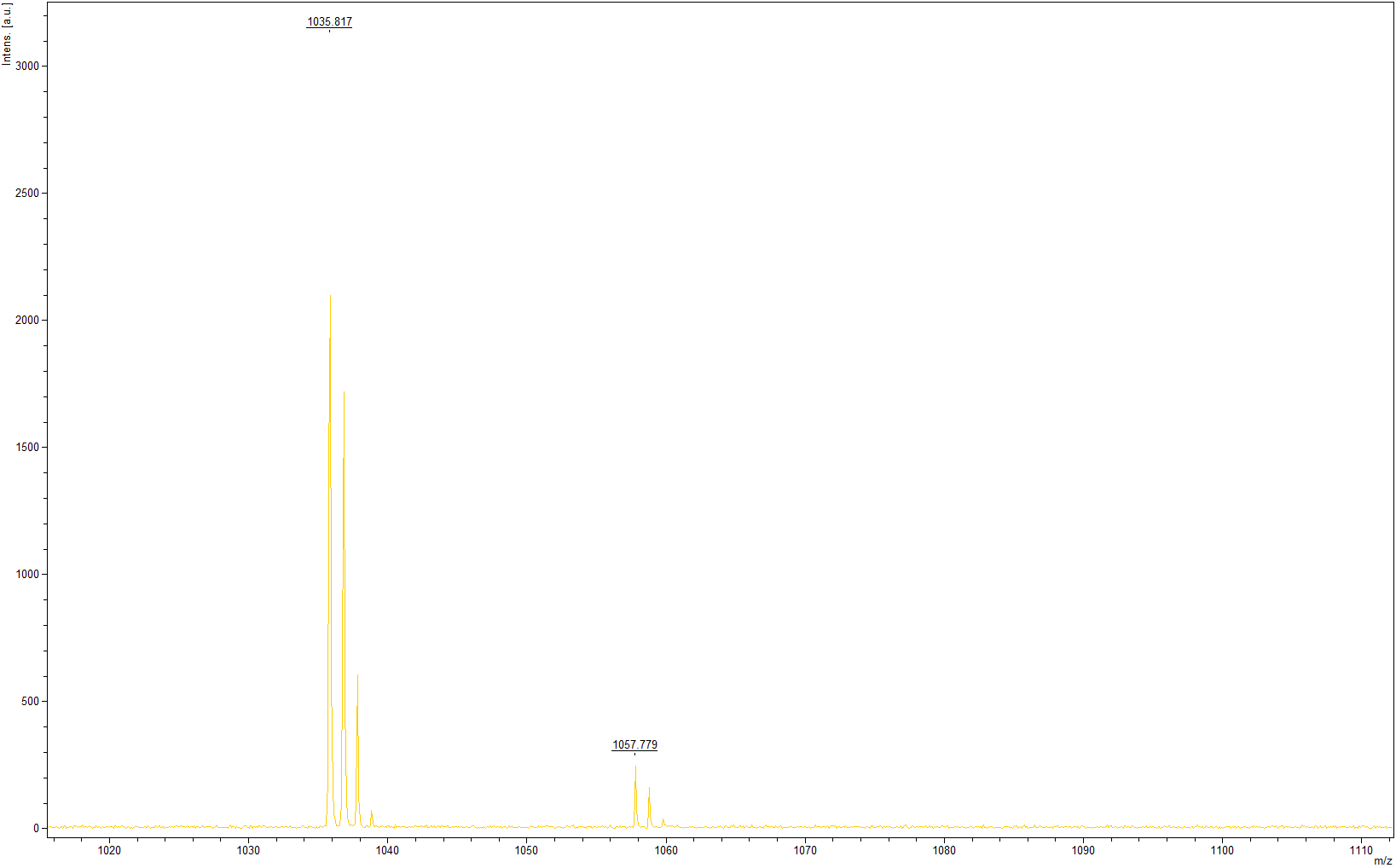


**Figure S99.** Mass spectrum of **A6-4O16**. MALDI-TOF MS m/z of [M + H]^+^ calculated for C_67_H_139_N_2_O_4_: 1036.069; Found: 1035.817.

**Figure S100.** ^1^H NMR of **A6-3O18**. ^1^H NMR (500 MHz, Chloroform-d) δ 3.75 – 3.58 (m, 3H), 2.86 – 2.17 (m, 10H), 1.74 – 1.56 (m, 2H), 1.48 – 1.12 (m, 90H), 0.88 (t, *J* = 6.9 Hz, 9H).


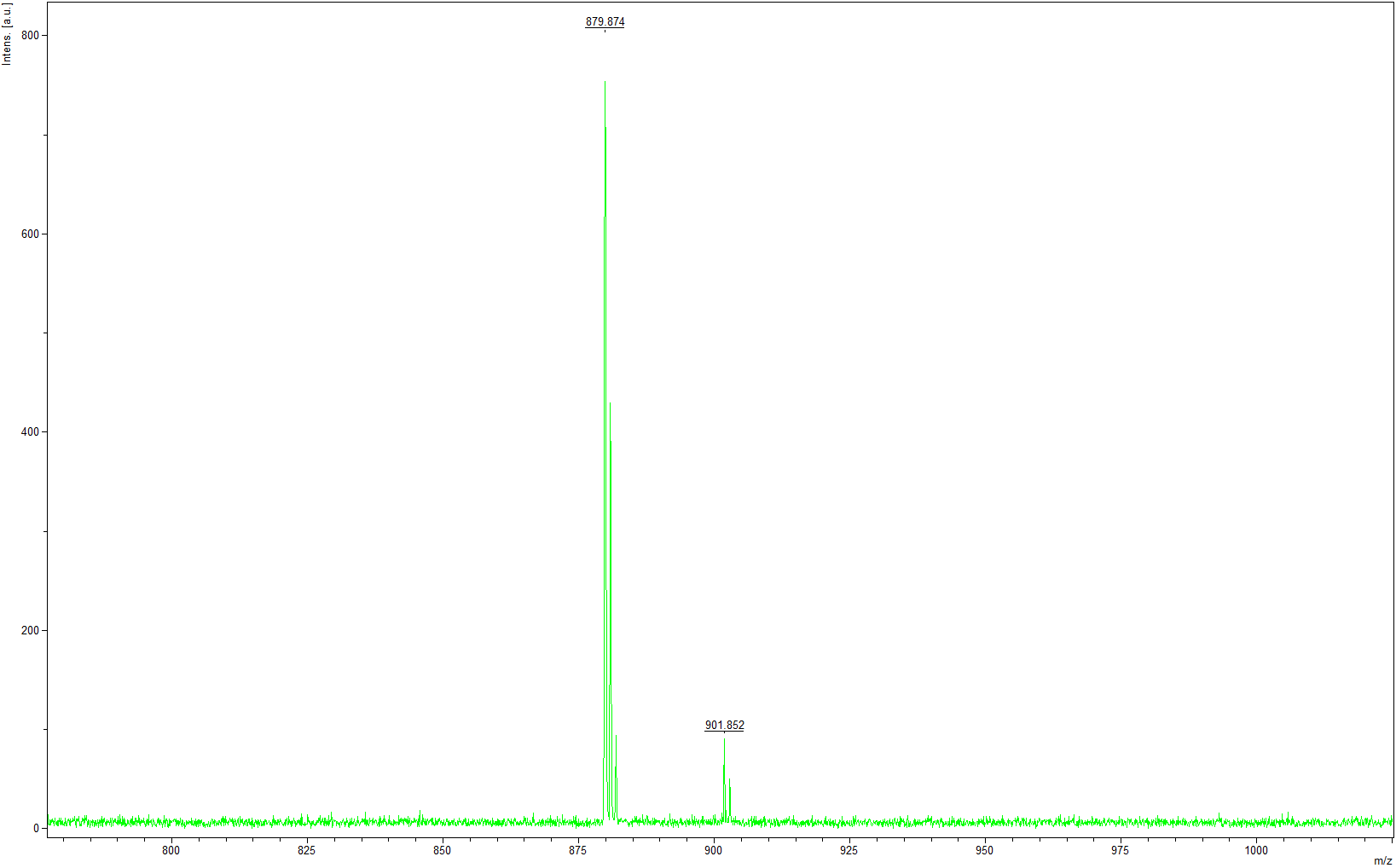


**Figure S101.** Mass spectrum of **A6-3O18**. MALDI-TOF MS m/z of [M + H]^+^ calculated for C_57_H_119_N_2_O_3_: 879.918; Found: 879.874.

**Figure S102.** ^1^H NMR of **A6-4O18**. ^1^H NMR (500 MHz, Chloroform-d) δ 3.85 – 3.56 (m, 4H), 2.96 – 2.08 (m, 12H), 1.82 – 1.61 (m, 2H), 1.54 – 1.16 (m, 120H), 0.88 (t, *J* = 6.9 Hz, 12H).


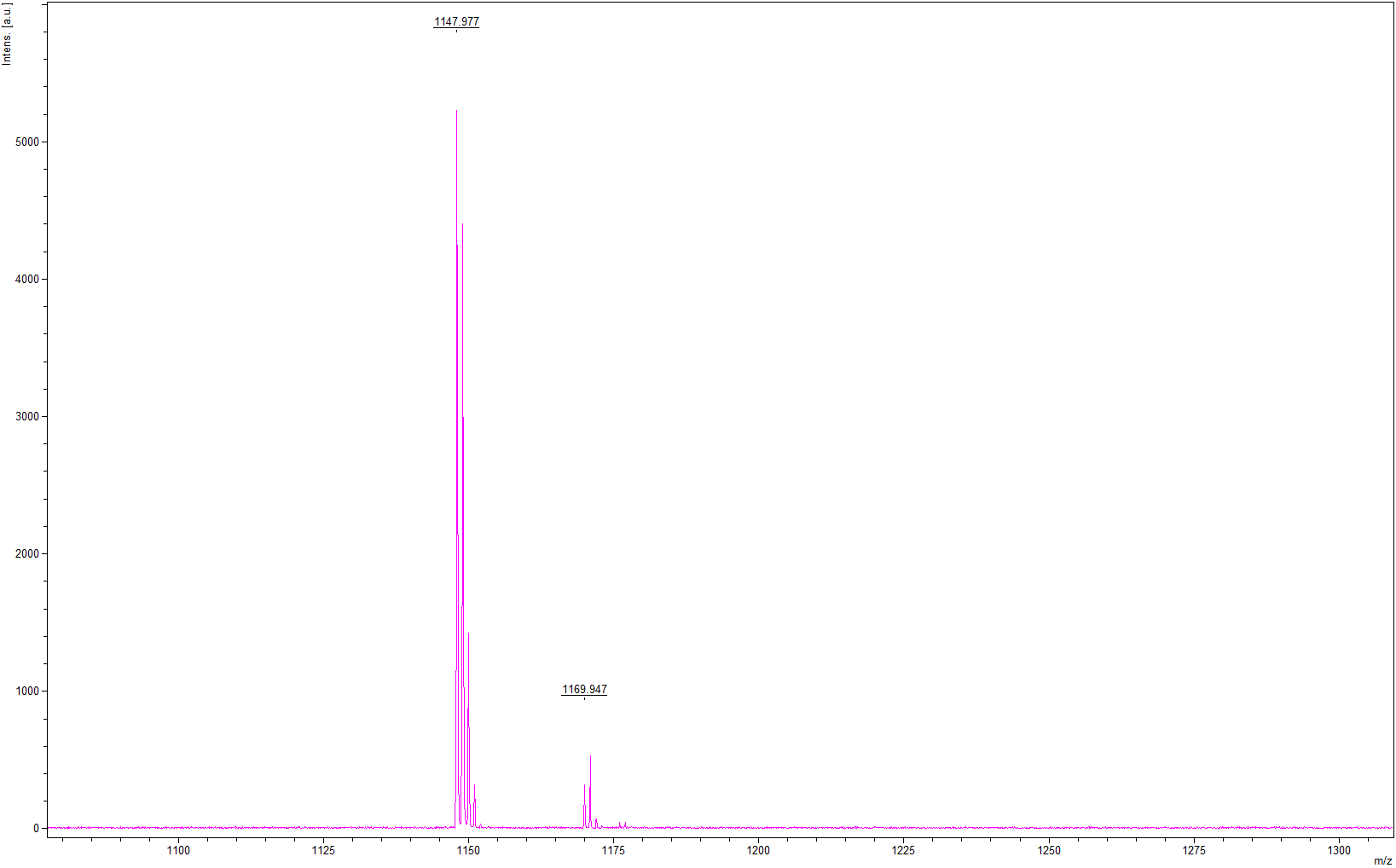


**Figure S103.** Mass spectrum of **A6-4O18**. MALDI-TOF MS m/z of [M + H]^+^ calculated for C_75_H_155_N_2_O_4_: 1148.194; Found: 1147.977.

**Figure S104.** ^1^H NMR of **A7-3O14**. ^1^H NMR (500 MHz, Chloroform-d) δ 3.83 – 3.55 (m, 3H), 2.83 – 2.02 (m, 13H), 1.84 – 1.51 (m, 2H), 1.49 – 1.10 (m, 66H), 0.88 (t, *J* = 6.9 Hz, 9H).


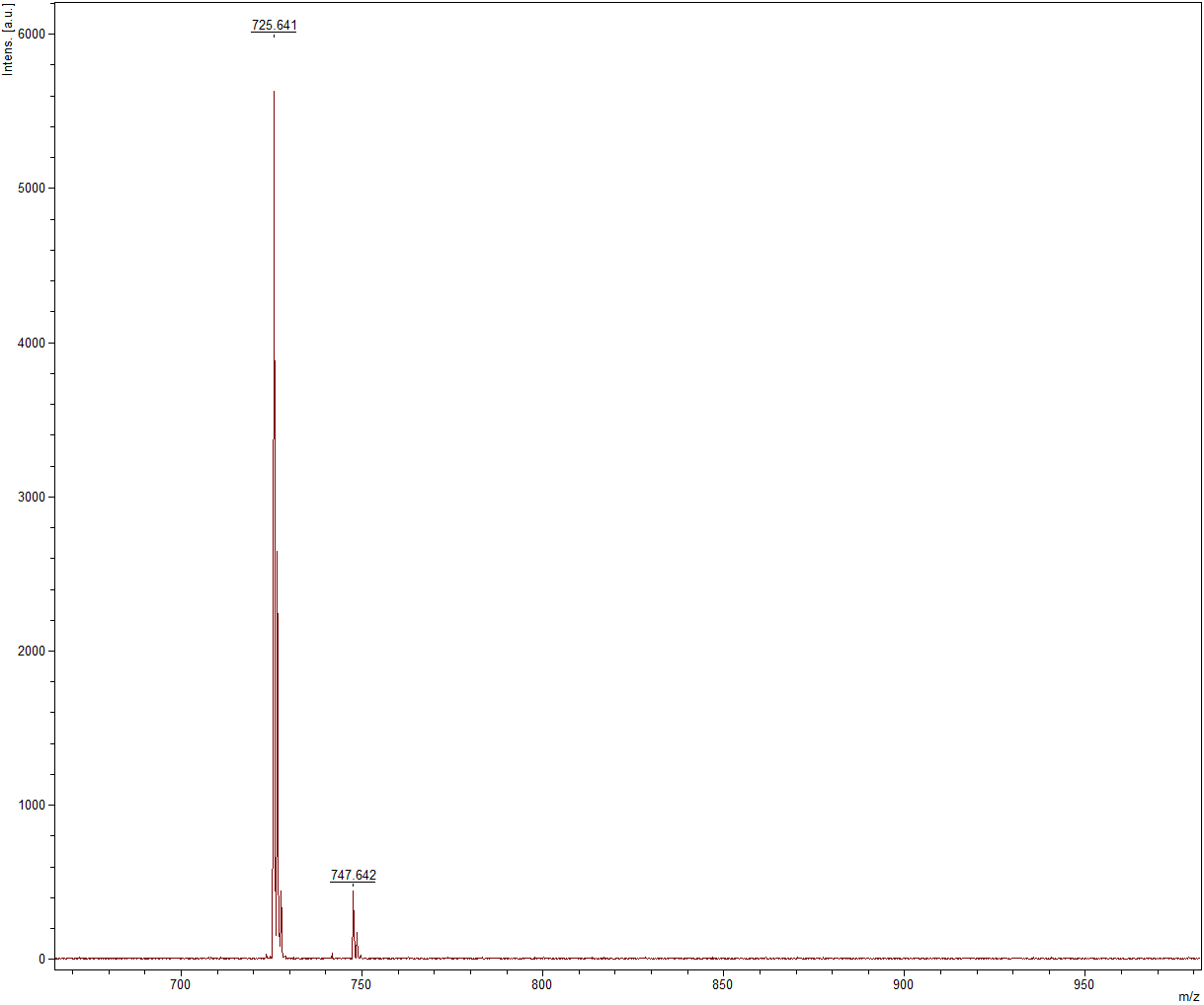


**Figure S105.** Mass spectrum of **A7-3O14**. MALDI-TOF MS m/z of [M + H]^+^ calculated for C_46_H_97_N_2_O_3_: 725.745; Found: 725.641.

**Figure S106.** ^1^H NMR of **A8-3O14**. ^1^H NMR (500 MHz, Chloroform-d) δ 3.704 – 3.599 (m, 3H), 2.87 – 2.18 (m, 12H), 1.77 – 1.57 (m, 2H), 1.53 – 1.16 (m, 66H), 1.04 (t, *J* = 7.3 Hz, 3H), 0.88 (t, *J* = 6.9 Hz, 9H).


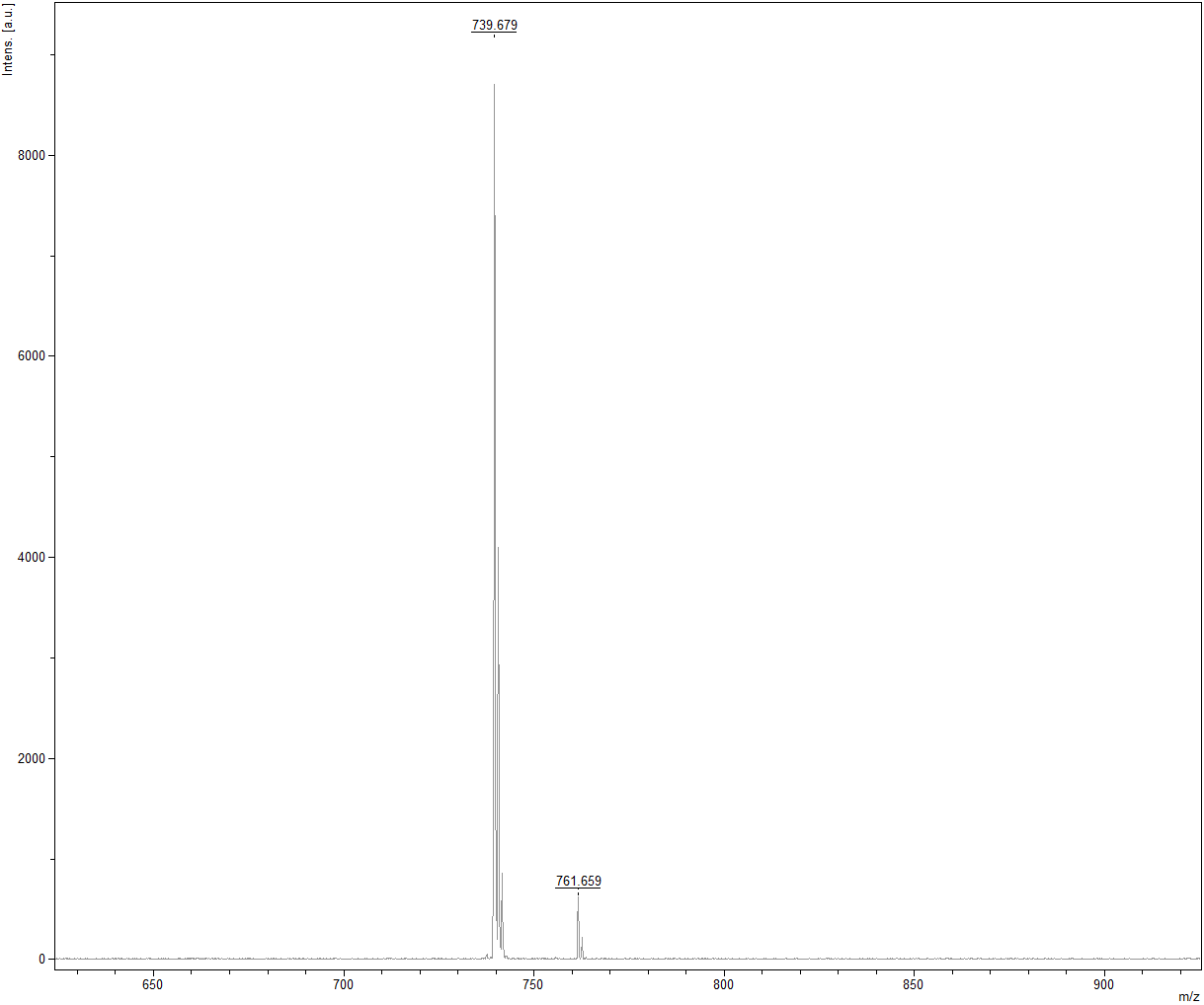


**Figure S107.** Mass spectrum of **A8-3O14**. MALDI-TOF MS m/z of [M + H]^+^ calculated for C_47_H_99_N_2_O_3_: 739.761, Found: 739.679.

**Figure S108.** ^1^H NMR of **A9-3O14**. ^1^H NMR (500 MHz, Chloroform-d) δ 3.72 – 3.59 (m, 3H), 2.81 – 2.20 (m, 12H), 1.77 – 1.56 (m, 2H), 1.51 – 1.17 (m, 68H), 0.88 (t, *J* = 6.9 Hz, 12H).


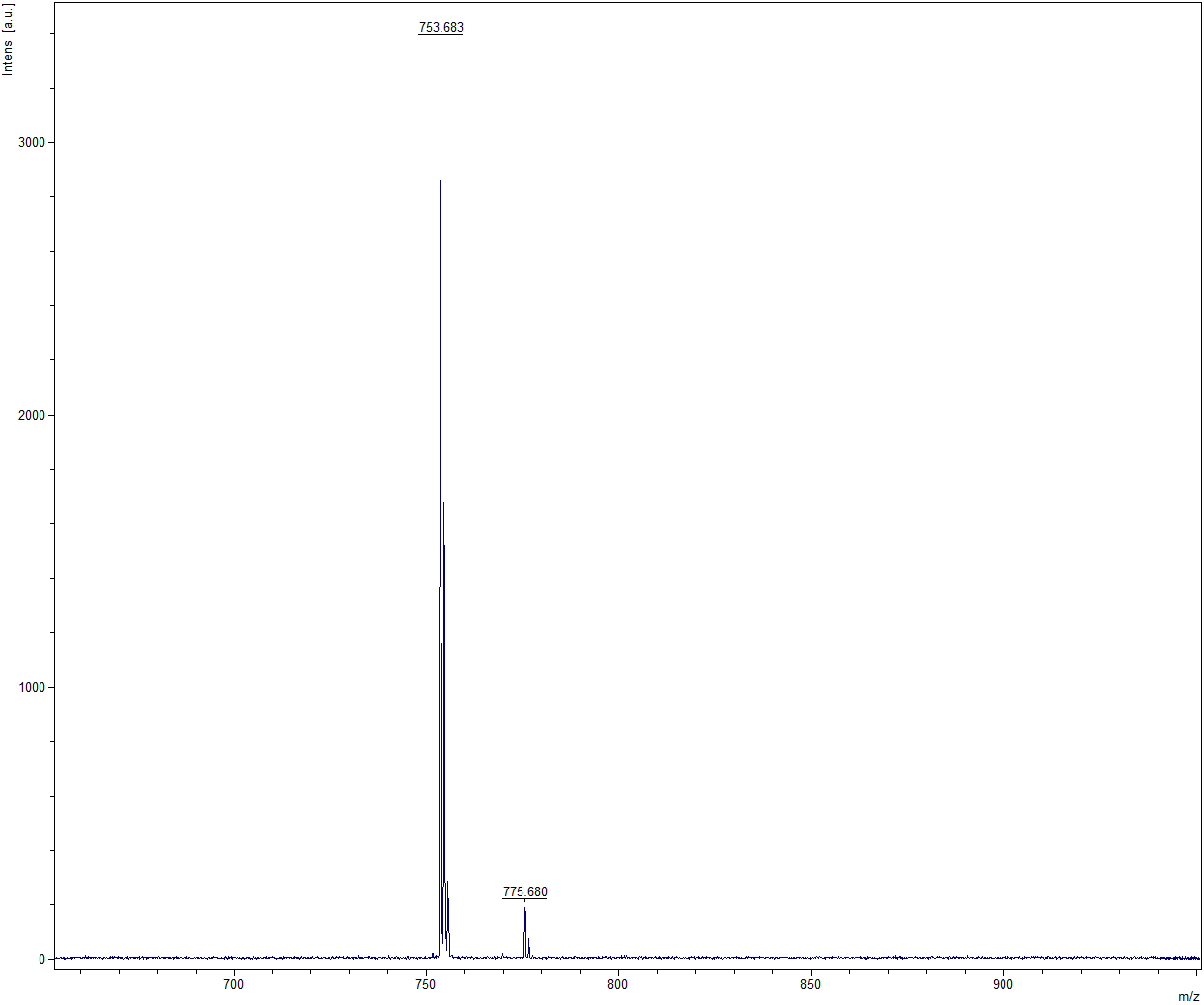


**Figure S109.** Mass spectrum of **A9-3O14**. MALDI-TOF MS m/z of [M + H]^+^ calculated for C_48_H_101_N_2_O_3_: 753.777, Found: 753.683.

**Figure S110.** ^1^H NMR of **A1-3B12**. ^1^H NMR (500 MHz, Chloroform-d) δ 4.06 (dt, *J* = 9.0, 6.8 Hz, 6H), 3.64 – 3.46 (m, 8H), 3.01 – 2.61 (m, 10H), 2.48 (dt, *J* = 35.7, 6.9 Hz, 6H), 1.71 – 1.51 (m, 6H), 1.41 – 1.14 (m, 54H), 0.88 (t, *J* = 6.9 Hz, 9H).


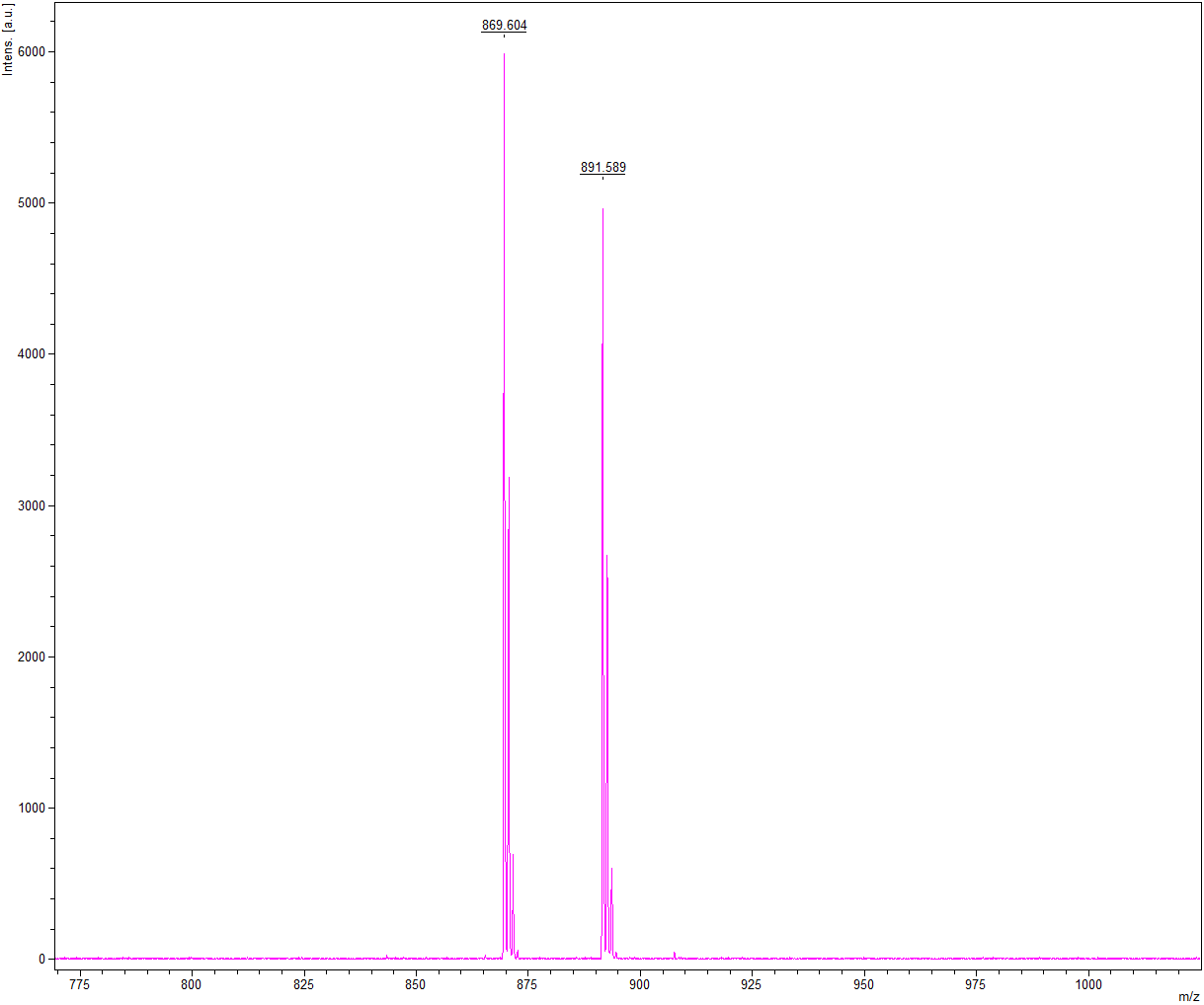


**Figure S111.** Mass spectrum of **A1-3B12**. MALDI-TOF MS m/z of [M + H]^+^ calculated for C_51_H_101_N_2_O_8_: 869.751, Found: 869.604.

**Figure S112.** ^1^H NMR of **A1-3B14**. ^1^H NMR (500 MHz, Chloroform-d) δ 4.13 – 4.00 (m, 6H), 3.70 – 3.45 (m, 8H), 2.99 – 2.75 (m, 8H), 2.68 (t, *J* = 6.3 Hz, 2H), 2.48 (dt, *J* = 38.0, 7.0 Hz, 6H), 1.70 – 1.54 (m, 6H), 1.42 – 1.19 (m, 66H), 0.88 (t, *J* = 6.9 Hz, 9H).


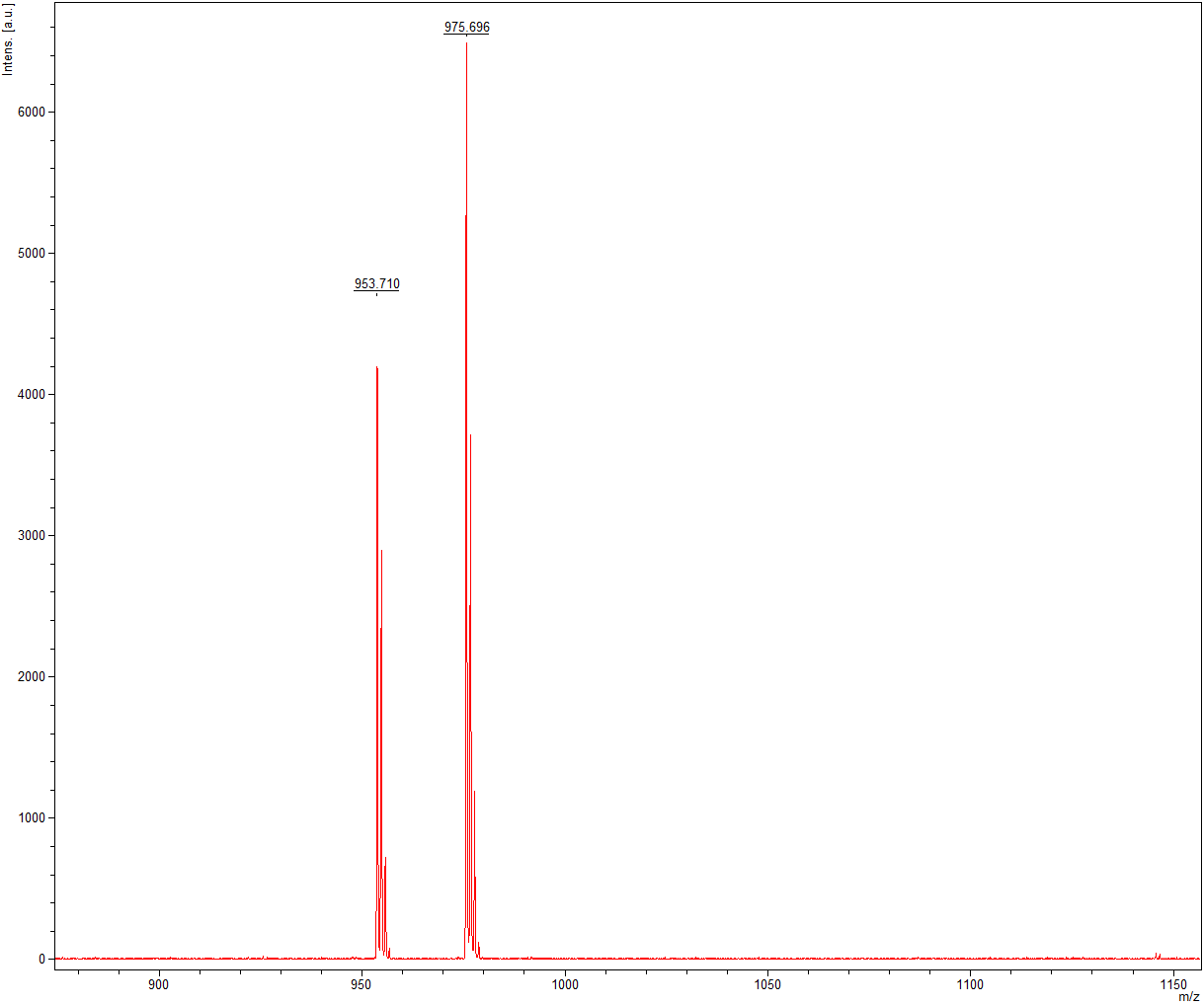


**Figure S113.** Mass spectrum of **A1-3B14**. MALDI-TOF MS m/z of [M + H]^+^ calculated for C_57_H_113_N_2_O_8_: 953.845, Found: 953.710; [M + Na]^+^ calculated for C_57_H_112_N_2_NaO_8_: 975.832, Found: 975.696.

**Figure S114.** ^1^H NMR of **A1-3B16**. ^1^H NMR (500 MHz, Chloroform-d) δ 4.06 (dt, *J* = 9.8, 6.8 Hz, 6H), 3.64 – 3.37 (m, 8H), 2.97 – 2.74 (m, 8H), 2.68 (t, *J* = 6.3 Hz, 2H), 2.48 (dt, *J* = 35.2, 7.0 Hz, 6H), 1.71 – 1.52 (m, 6H), 1.38 – 1.17 (m, 78H), 0.88 (t, *J* = 6.9 Hz, 9H).


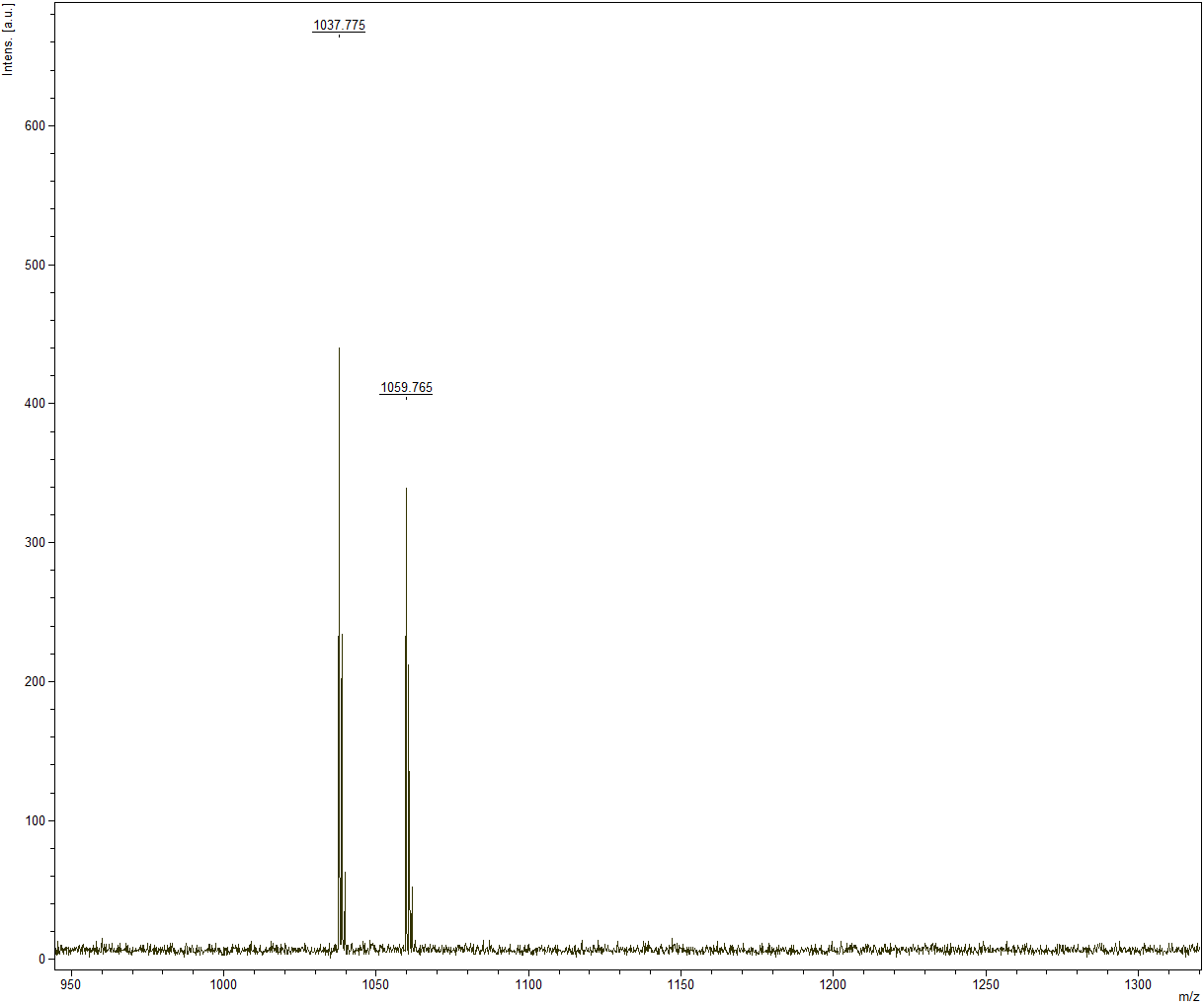


**Figure S115.** Mass spectrum of **A1-3B16**. MALDI-TOF MS m/z of [M + H]^+^ calculated for C_63_H_125_N_2_O_8_: 1037.939, Found: 1037.775.

**Figure S116.** ^1^H NMR of **A1-3B18**. ^1^H NMR (500 MHz, Chloroform-d) δ 4.06 (dt, *J* = 9.9, 6.8 Hz, 6H), 3.68 – 3.44 (m, 8H), 3.02 – 2.74 (m, 8H), 2.68 (t, *J* = 6.3 Hz, 2H), 2.48 (dt, *J* = 35.0, 7.0 Hz, 6H), 1.68 – 1.52 (m, 6H), 1.30 – 1.25 (m, 90H), 0.88 (t, *J* = 6.9 Hz, 9H).


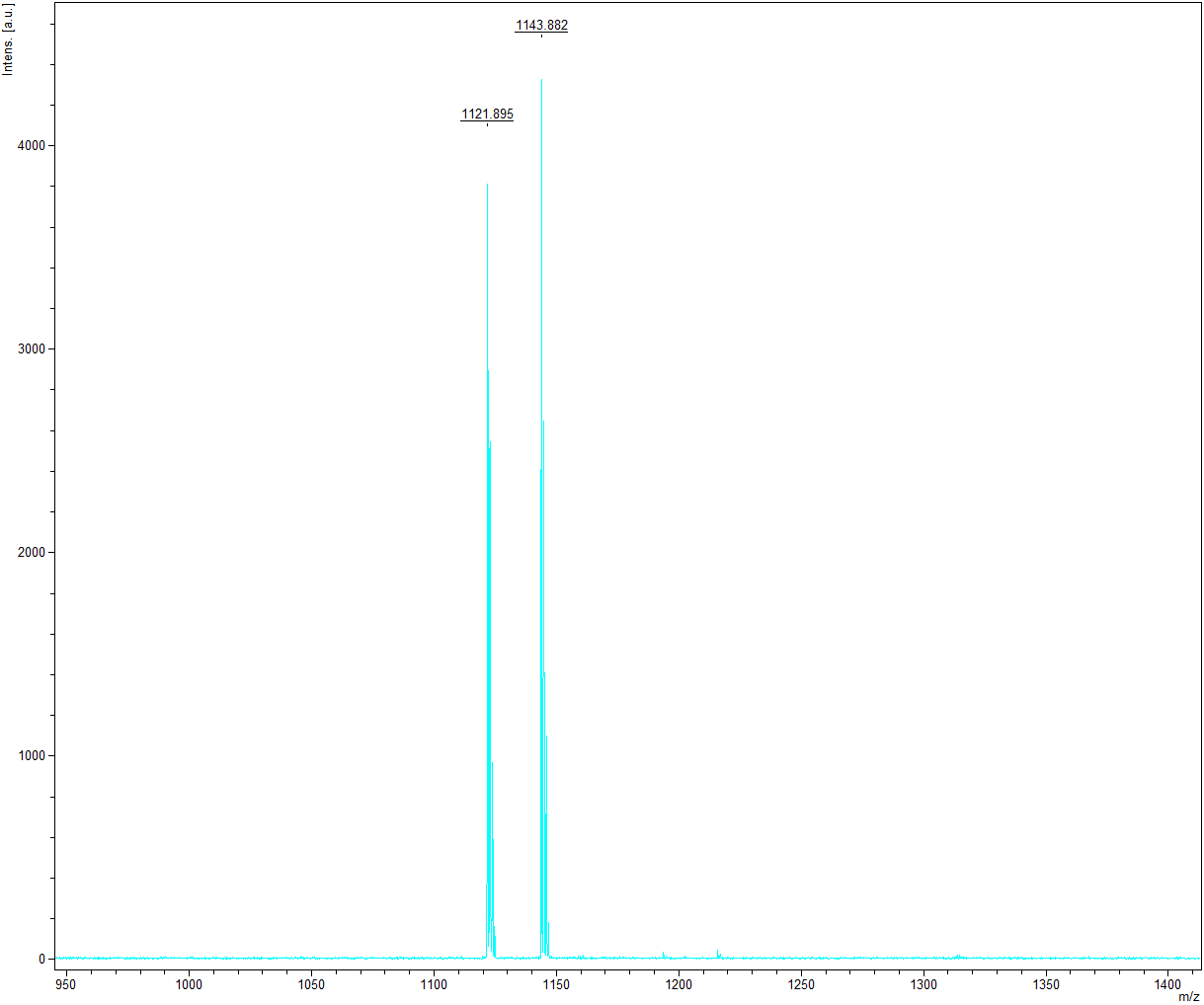


**Figure S117.** Mass spectrum of **A1-3B18**. MALDI-TOF MS m/z of [M + H]^+^ calculated for C_69_H_137_N_2_O_8_: 1122.033, Found: 1121.895; [M + Na]^+^ calculated for C_69_H_136_N_2_NaO_8_:1144.019, Found: 1143.882.

**Figure S118.** ^1^H NMR of **A2-3B12**. ^1^H NMR (500 MHz, Chloroform-d) δ 4.06 (dt, *J* = 9.7, 6.8 Hz, 6H), 2.88 (t, *J* = 6.6 Hz, 2H), 2.76 (t, *J* = 7.3 Hz, 4H), 2.65 (t, *J* = 7.1 Hz, 2H), 2.52 (t, *J* = 6.6 Hz, 2H), 2.43 (t, *J* = 7.3 Hz, 6H), 2.33 (dt, *J* = 30.4, 7.3 Hz, 4H), 2.19 (s, 3H), 1.71 – 1.55 (m, 10H), 1.34 – 1.19 (m, 54H), 0.88 (t, *J* = 6.9 Hz, 9H).


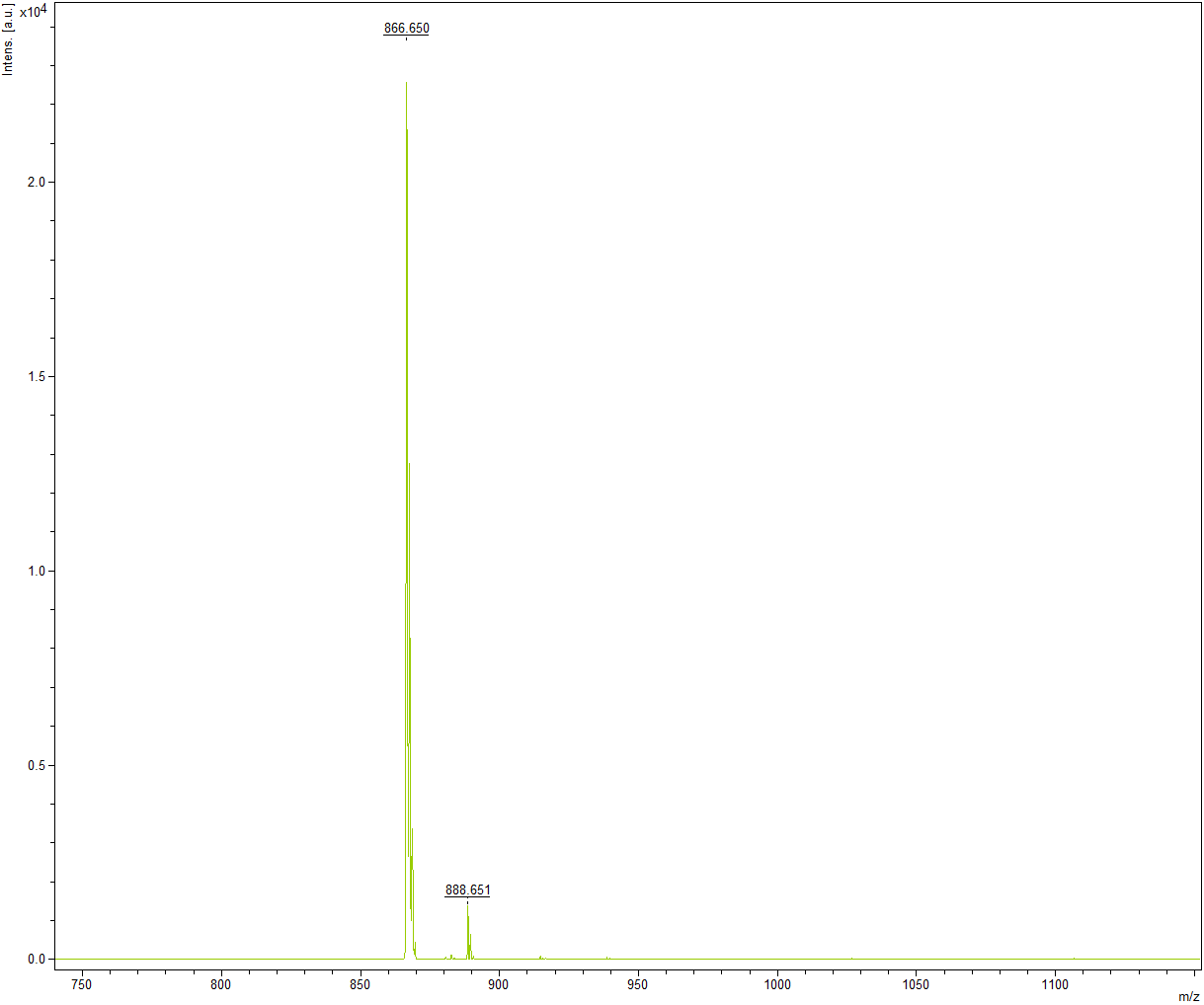


**Figure S119.** Mass spectrum of **A2-3B12**. MALDI-TOF MS m/z of [M + H]^+^ calculated for C_52_H_104_N_3_O_6_: 866.788, Found: 866.650.

**Figure S120.** ^1^H NMR of **A2-3B14**. ^1^H NMR (500 MHz, Chloroform-d) δ 4.06 (dt, *J* = 9.8, 6.8 Hz, 6H), 2.89 (t, *J* = 6.5 Hz, 2H), 2.76 (t, *J* = 7.3 Hz, 4H), 2.68 (t, *J* = 7.0 Hz, 2H), 2.54 (t, *J* = 6.5 Hz, 2H), 2.48 – 2.27 (m, 10H), 2.21 (s, 3H), 1.72 – 1.55 (m, 10H), 1.36 – 1.19 (m, 66H), 0.88 (t, *J* = 6.9 Hz, 9H).


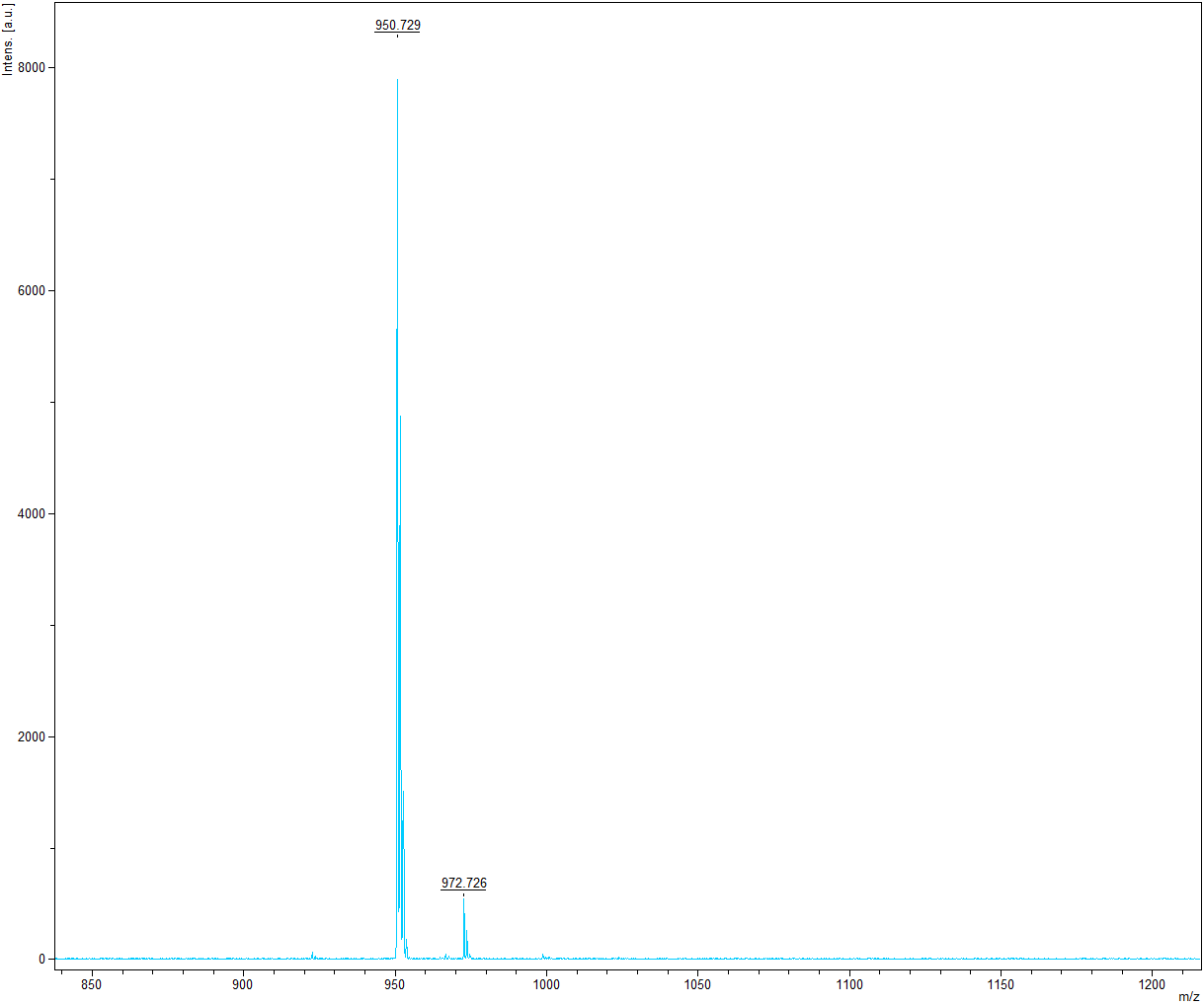


**Figure S121.** Mass spectrum of **A2-3B14**. MALDI-TOF MS m/z of [M + H]^+^ calculated for C_58_H_116_N_3_O_6_: 950.882, Found: 950.729.

**Figure S122.** ^1^H NMR of **A2-3B16**. ^1^H NMR (500 MHz, Chloroform-d) δ 4.06 (dt, *J* = 10.2, 6.8 Hz, 6H), 2.91 (t, *J* = 6.5 Hz, 2H), 2.74 (dt, *J* = 15.0, 6.9 Hz, 6H), 2.59 (t, *J* = 6.4 Hz, 2H), 2.48 – 2.31 (m, 10H), 2.23 (s, 3H), 1.78 – 1.54 (m, 10H), 1.35 – 1.23 (m, 78H), 0.88 (t, *J* = 6.9 Hz, 9H).


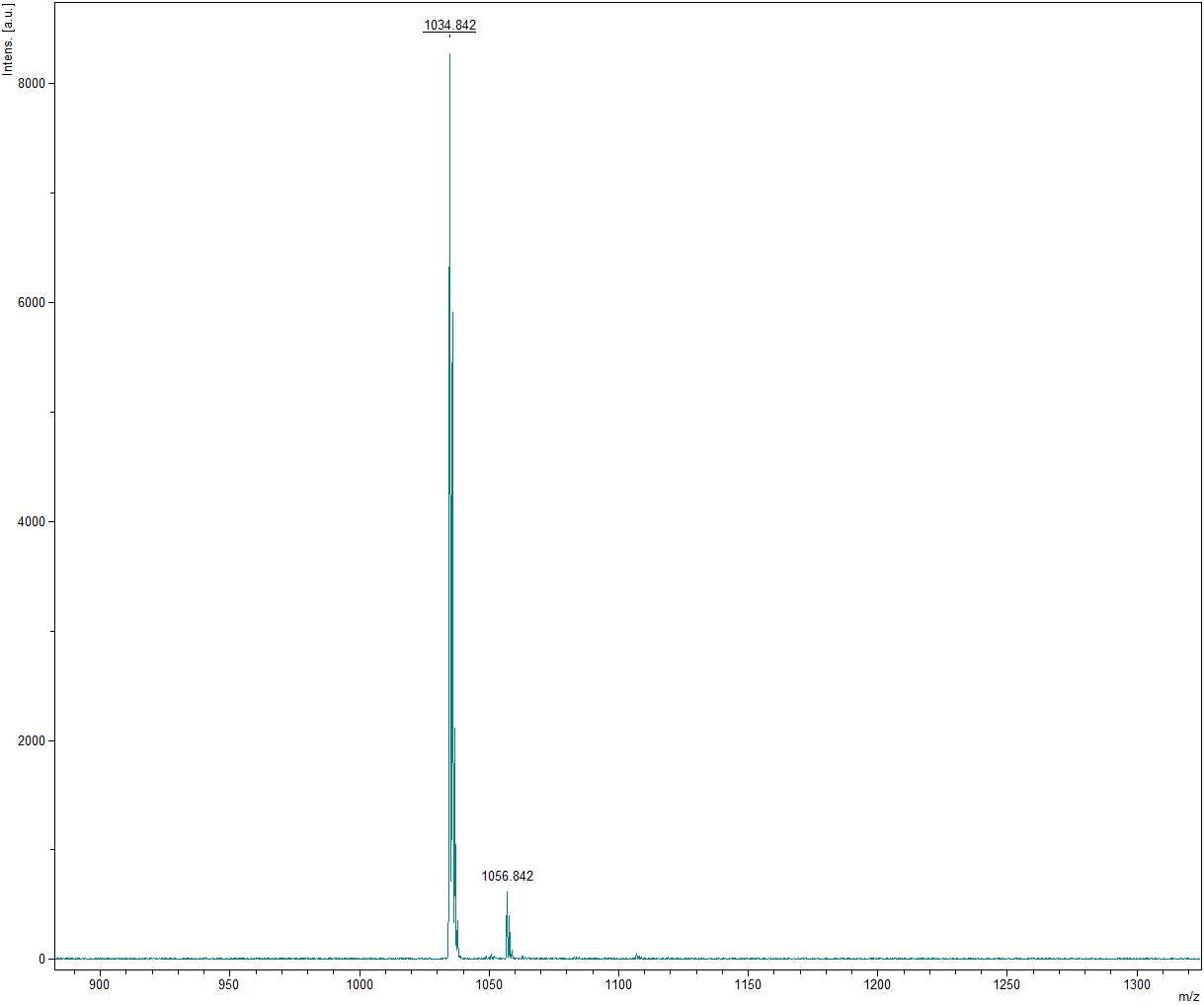


**Figure S123.** Mass spectrum of **A2-3B16**. MALDI-TOF MS m/z of [M + H]^+^ calculated for C_64_H_128_N_3_O_6_: 1034.976, Found: 1034.842.

**Figure S124.** ^1^H NMR of **A2-3B18**. ^1^H NMR (500 MHz, Chloroform-d) δ 4.06 (dt, *J* = 9.8, 6.8 Hz, 6H), 2.89 (t, *J* = 6.5 Hz, 2H), 2.76 (t, *J* = 7.2 Hz, 4H), 2.68 (t, *J* = 7.0 Hz, 2H), 2.55 (t, *J* = 6.5 Hz, 2H), 2.49 – 2.27 (m, 10H), 2.21 (s, 3H), 1.74 – 1.53 (m, 10H), 1.34 – 1.23 (m, 90H), 0.88 (t, *J* = 6.9 Hz, 9H).


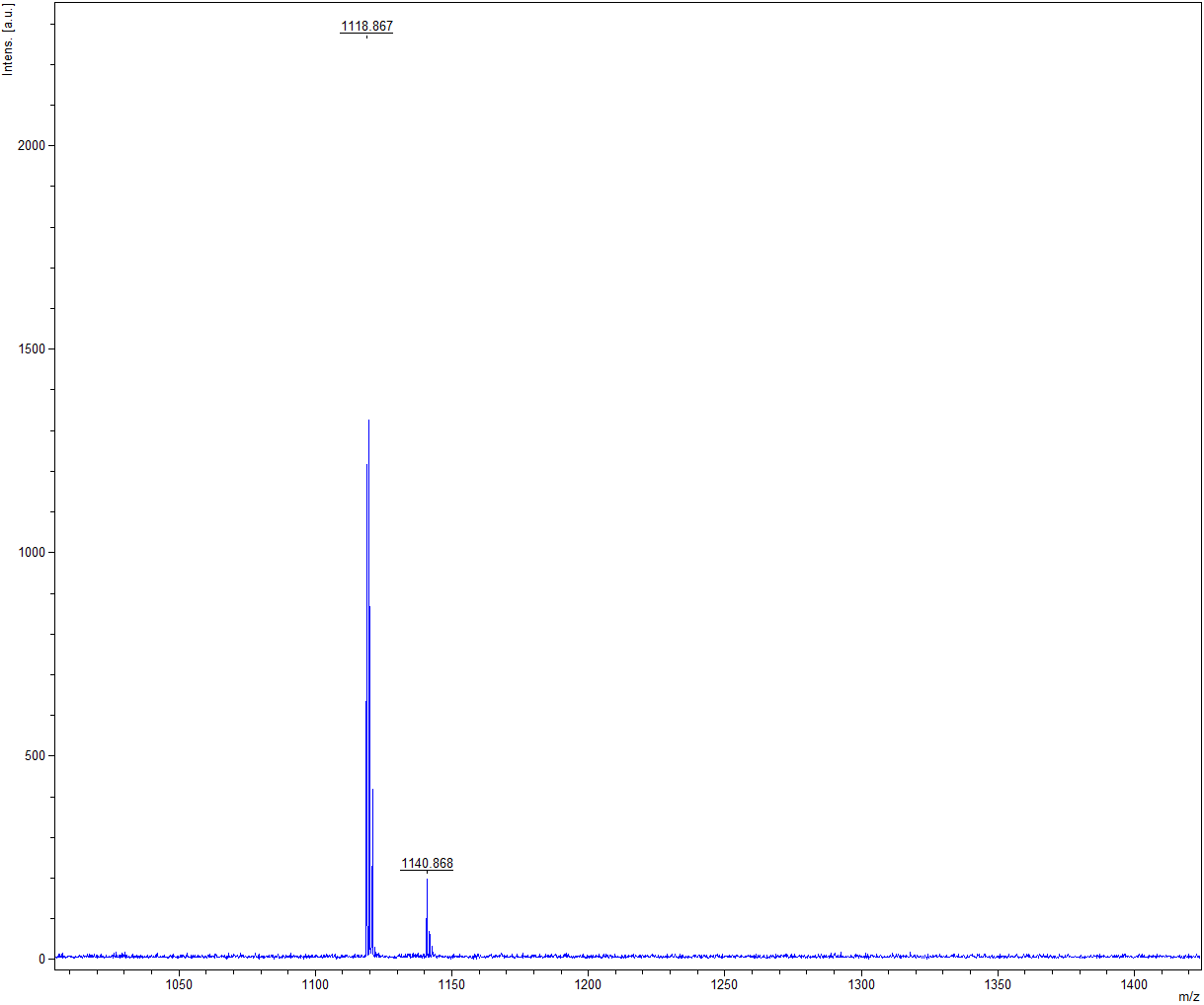


**Figure S125.** Mass spectrum of **A2-3B18**. MALDI-TOF MS m/z of [M + H]^+^ calculated for C_70_H_140_N_3_O_6_: 1119.070, Found: 1118.867.

**Figure S126.** ^1^H NMR of **A3-3B12**. ^1^H NMR (500 MHz, Chloroform-d) δ 4.06 (dt, *J* = 17.9, 6.7 Hz, 6H), 3.02 – 2.25 (m, 28H), 1.77 (t, *J* = 6.7 Hz, 2H), 1.69 – 1.54 (m, 8H), 1.38 – 1.18 (m, 54H), 0.88 (t, *J* = 6.9 Hz, 9H).


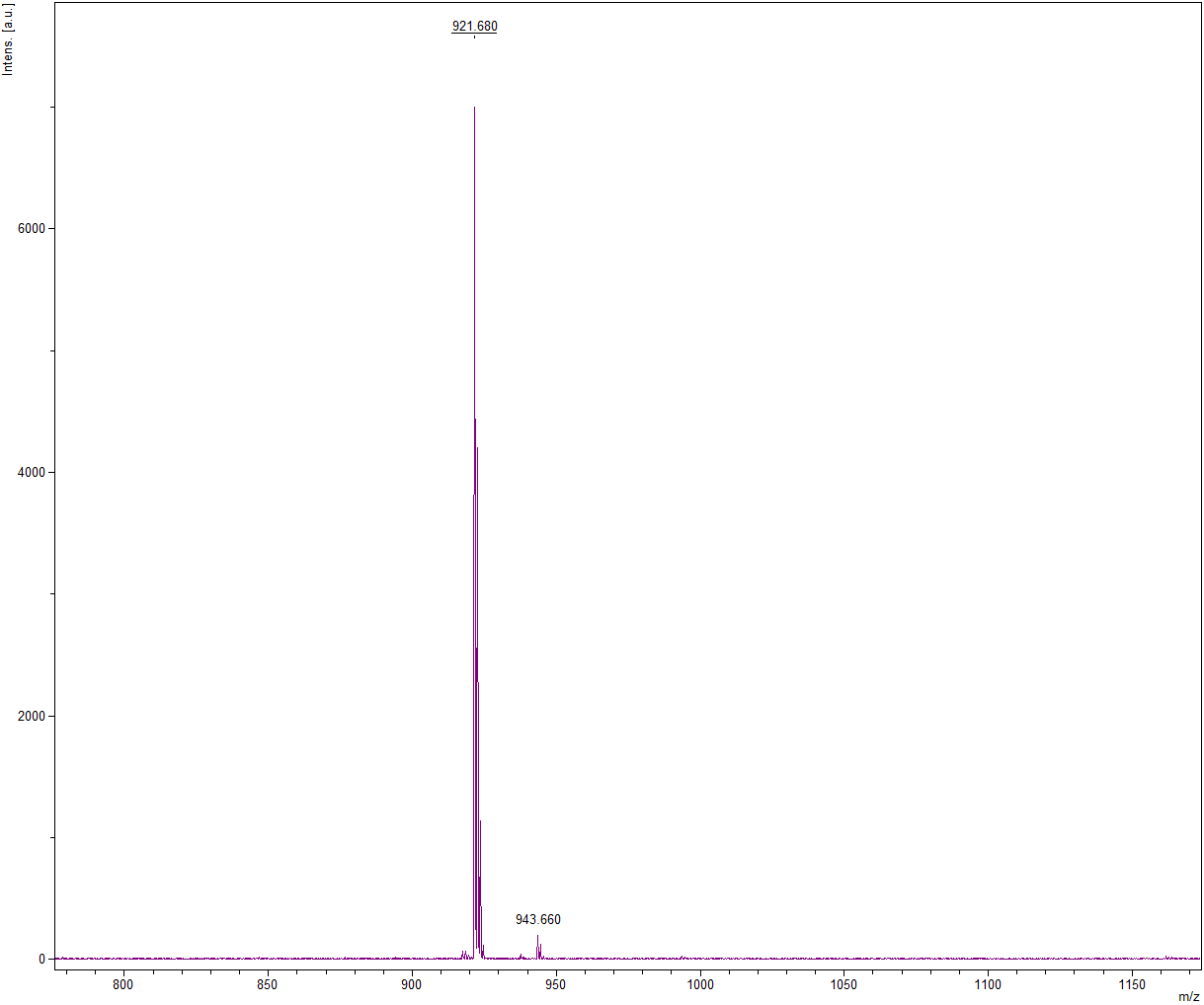


**Figure S127.** Mass spectrum of **A3-3B12**. MALDI-TOF MS m/z of [M + H]^+^ calculated for C_55_H_109_N_4_O_6_: 921.830, Found: 921.680.

**Figure S128.** ^1^H NMR of **A3-3B14**. ^1^H NMR (500 MHz, Chloroform-d) δ 4.07 (dt, *J* = 20.9, 6.8 Hz, 6H), 3.06 – 2.27 (m, 28H), 1.81 (t, *J* = 6.5 Hz, 2H), 1.61 (p, *J* = 7.0 Hz, 8H), 1.35 – 1.22 (m, 66H), 0.88 (t, *J* = 6.9 Hz, 9H).


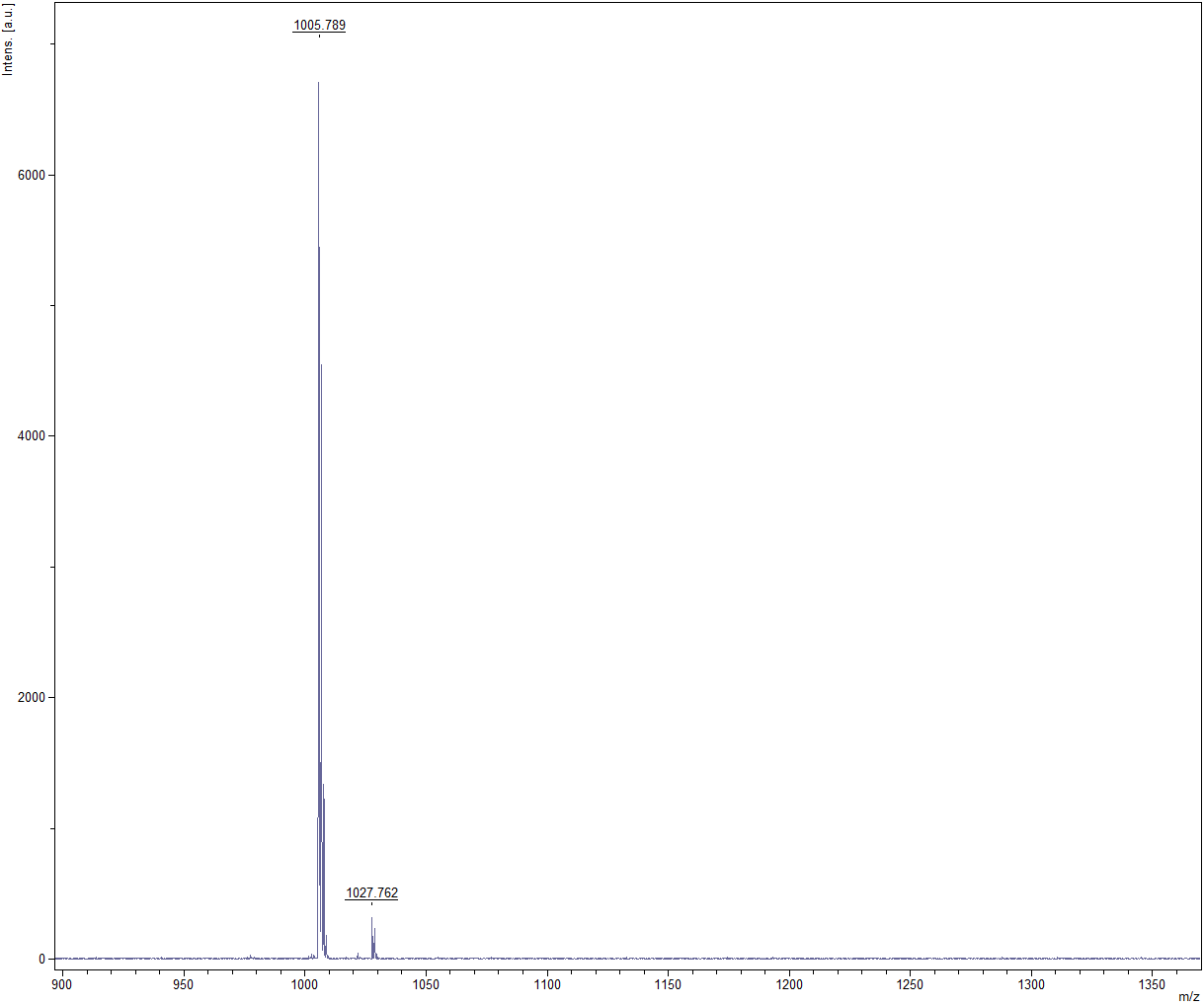


**Figure S129.** Mass spectrum of **A3-3B14**. MALDI-TOF MS m/z of [M + H]^+^ calculated for C_61_H_121_N_4_O_6_: 1005.924, Found: 1005.789.

**Figure S130.** ^1^H NMR of **A3-3B16**. ^1^H NMR (500 MHz, Chloroform-d) δ 4.06 (dt, *J* = 18.8, 6.8 Hz, 6H), 3.04 – 2.24 (m, 28H), 1.78 (p, *J* = 6.6 Hz, 2H), 1.61 (dq, *J* = 13.6, 6.5 Hz, 8H), 1.31 – 1.21 (m, 78H), 0.88 (t, *J* = 6.9 Hz, 9H).


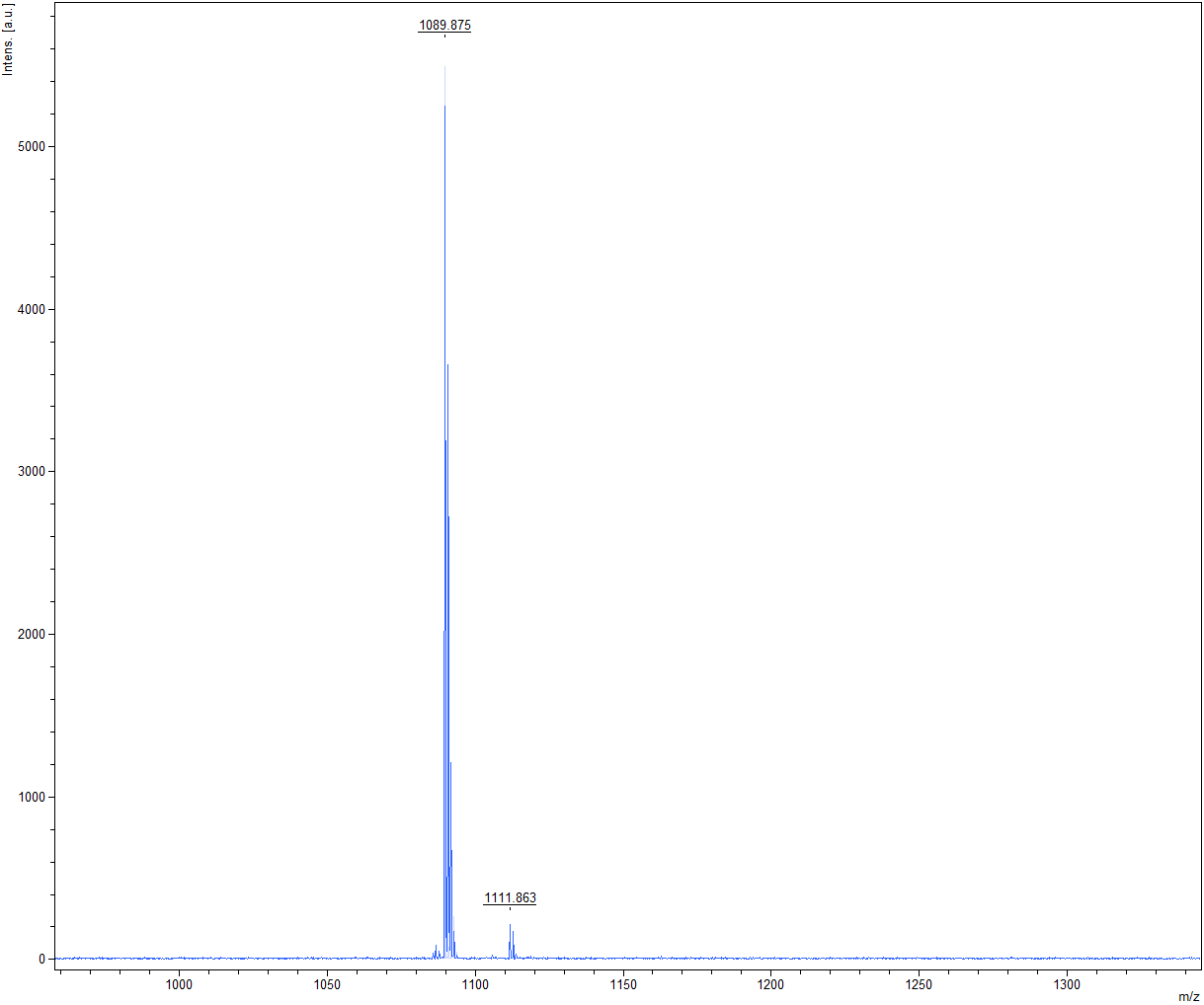


**Figure S131.** Mass spectrum of **A3-3B16**. MALDI-TOF MS m/z of [M + H]^+^ calculated for C_67_H_133_N_4_O_6_: 1090.018, Found: 1089.875.

**Figure S132.** ^1^H NMR of **A3-3B18**. ^1^H NMR (500 MHz, Chloroform-d) δ 4.06 (dt, *J* = 15.3, 6.8 Hz, 6H), 2.99 – 2.24 (m, 28H), 1.80 – 1.51 (m, 10H), 1.30 – 1.20 (m, 90H), 0.88 (t, *J* = 6.9 Hz, 9H).


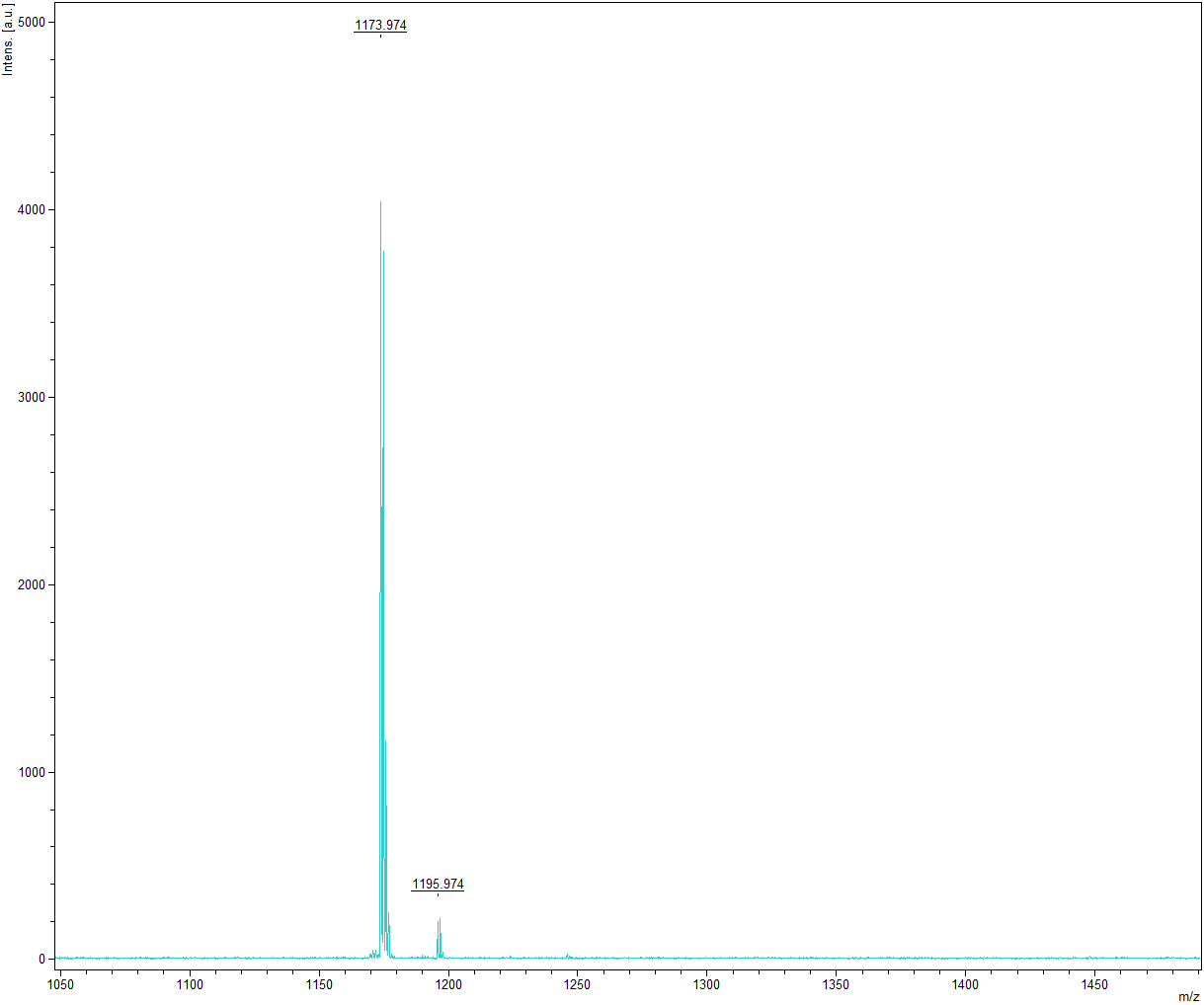


**Figure S133.** Mass spectrum of **A3-3B18**. MALDI-TOF MS m/z of [M + H]^+^ calculated for C_73_H_145_N_4_O_6_: 1174.112, Found: 1173.974.

**Figure S134.** ^1^H NMR of **A5-3B12**. ^1^H NMR (500 MHz, Chloroform-d) δ 4.06 (dt, *J* = 11.5, 6.8 Hz, 6H), 2.95 (t, *J* = 6.7 Hz, 2H), 2.77 (dt, *J* = 19.8, 6.6 Hz, 6H), 2.64 – 2.51 (m, 6H), 2.46 (dt, *J* = 14.4, 7.5 Hz, 6H), 2.25 (s, 3H), 1.61 (dtd, *J* = 13.2, 6.7, 3.3 Hz, 6H), 1.43 – 1.16 (m, 54H), 0.88 (t, *J* = 6.9 Hz, 9H).


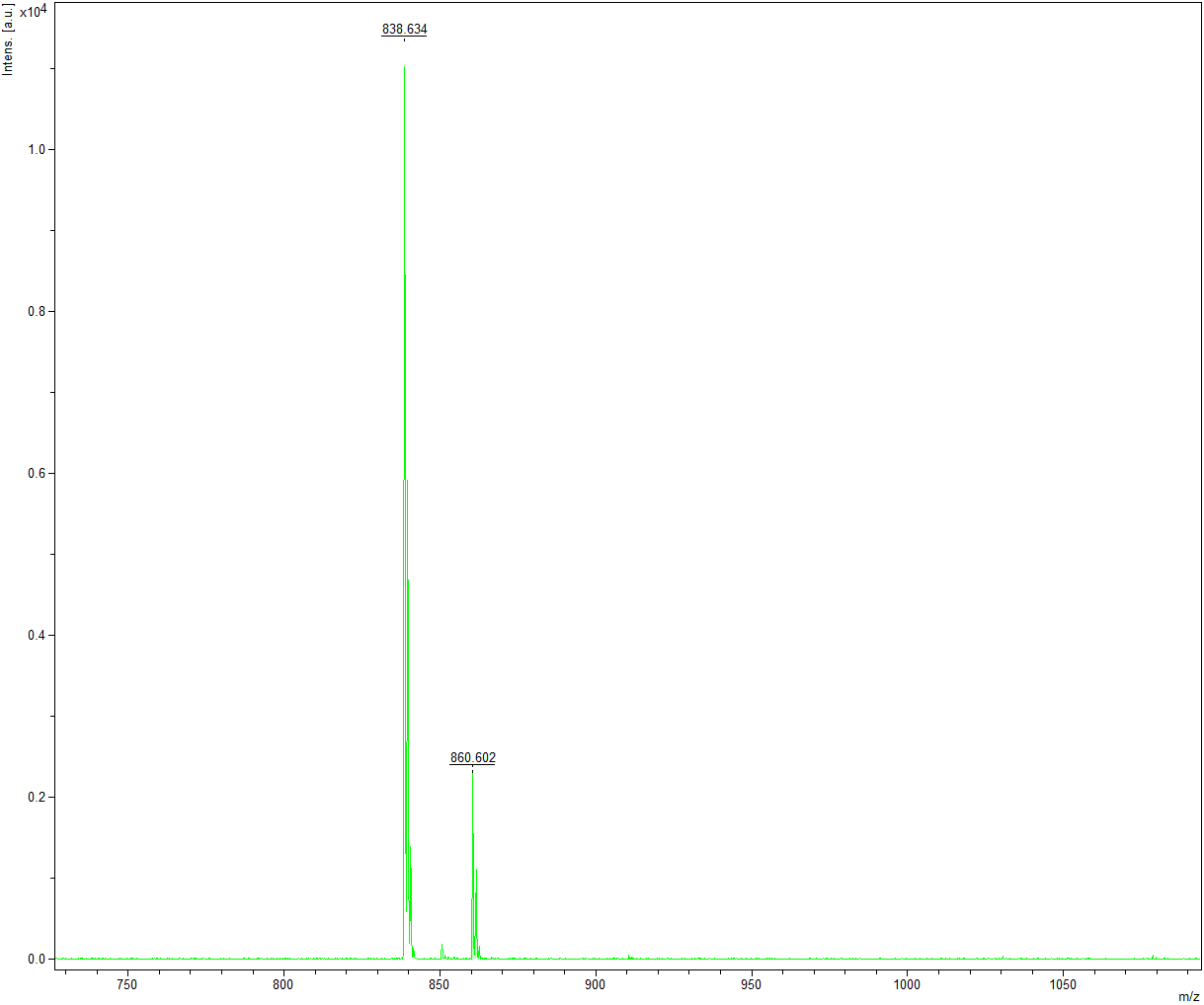


**Figure S135.** Mass spectrum of **A5-3B12**. MALDI-TOF MS m/z of [M + H]^+^ calculated for C_50_H_100_N_3_O_6_: 838.757, Found: 838.634.

**Figure S136.** ^1^H NMR of **A5-3B14**. ^1^H NMR (500 MHz, Chloroform-d) δ 4.06 (dt, *J* = 9.9, 6.8 Hz, 6H), 2.92 (t, *J* = 6.7 Hz, 2H), 2.75 (dt, *J* = 37.7, 6.6 Hz, 6H), 2.61 – 2.40 (m, 12H), 2.23 (s, 3H), 1.61 (tt, *J* = 8.3, 4.2 Hz, 6H), 1.41 – 1.17 (m, 66H), 0.88 (t, *J* = 6.9 Hz, 9H).


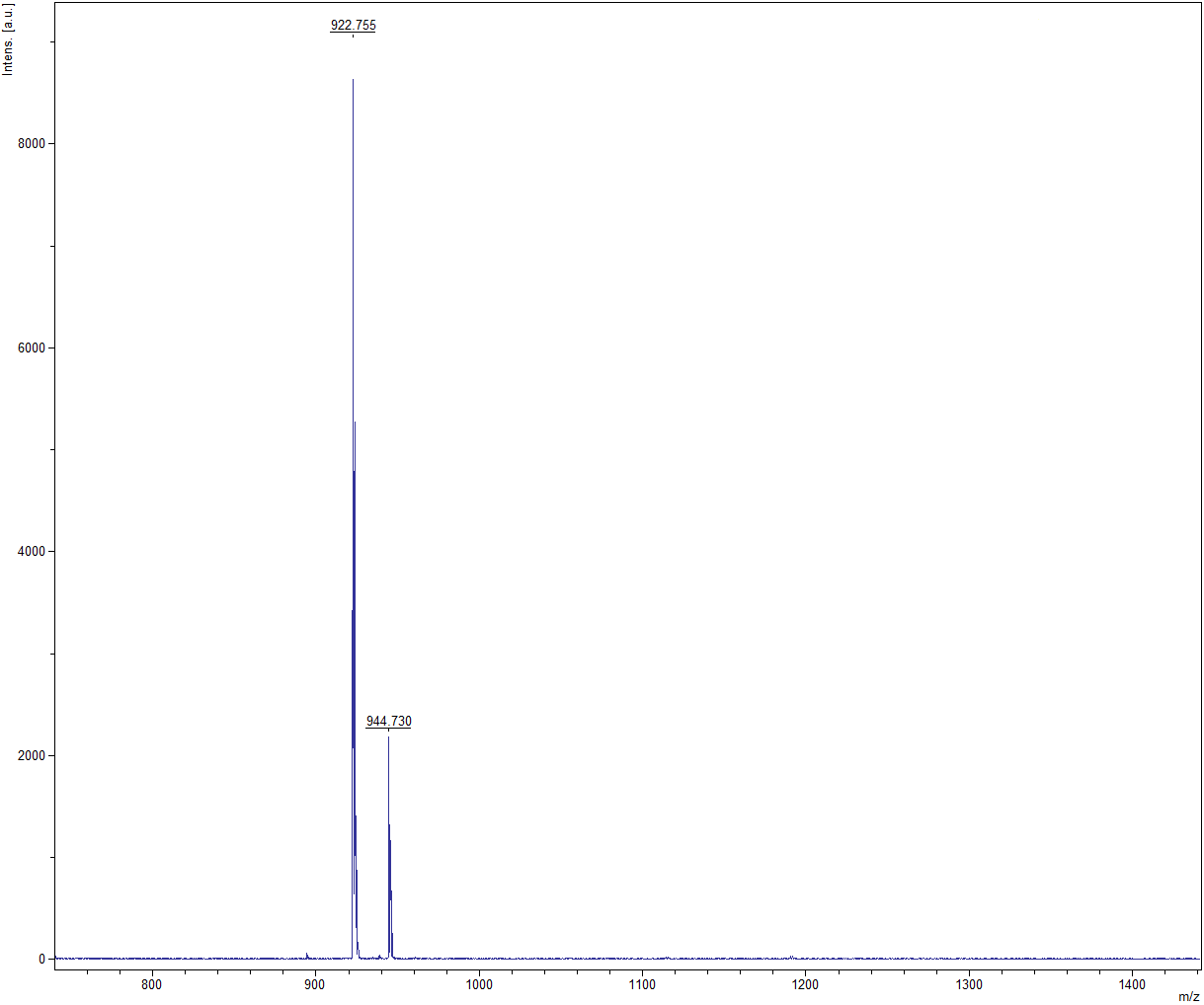


**Figure S137.** Mass spectrum of **A5-3B14**. MALDI-TOF MS m/z of [M + H]^+^ calculated for C_56_H_112_N_3_O_6_: 922.851, Found: 922.755.

**Figure S138.** ^1^H NMR of **A5-3B16**. ^1^H NMR (500 MHz, Chloroform-d) δ 4.06 (dt, *J* = 10.9, 6.8 Hz, 6H), 2.94 (t, *J* = 6.7 Hz, 2H), 2.76 (dt, *J* = 25.7, 6.6 Hz, 6H), 2.61 – 2.39 (m, 12H), 2.24 (s, 3H), 1.67 – 1.56 (m, 6H), 1.32 – 1.23 (m, 78H), 0.88 (t, *J* = 6.9 Hz, 9H).


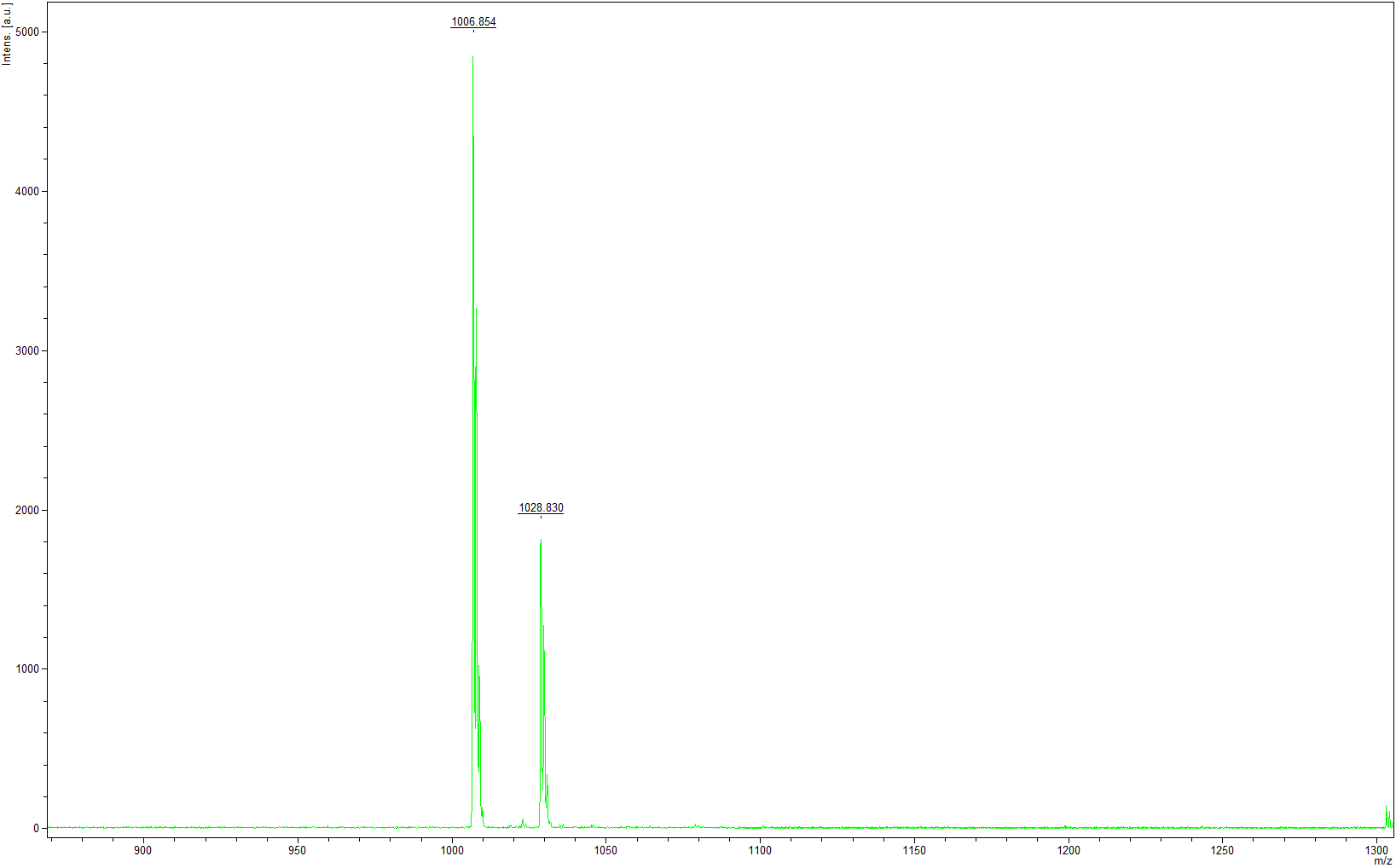


**Figure S139.** Mass spectrum of **A5-3B16**. MALDI-TOF MS m/z of [M + H]^+^ calculated for C_62_H_124_N_3_O_6_: 1006.945, Found: 1006.854.

**Figure S140.** ^1^H NMR of **A5-3B18**. ^1^H NMR (500 MHz, Chloroform-d) δ 4.06 (dt, *J* = 11.6, 6.8 Hz, 6H), 2.95 (t, *J* = 6.7 Hz, 2H), 2.77 (dt, *J* = 17.1, 6.7 Hz, 6H), 2.66 – 2.38 (m, 12H), 2.25 (s, 3H), 1.61 (dtd, *J* = 13.2, 6.8, 3.3 Hz, 6H), 1.35 – 1.21 (s, 90H), 0.88 (t, *J* = 6.9 Hz, 9H).


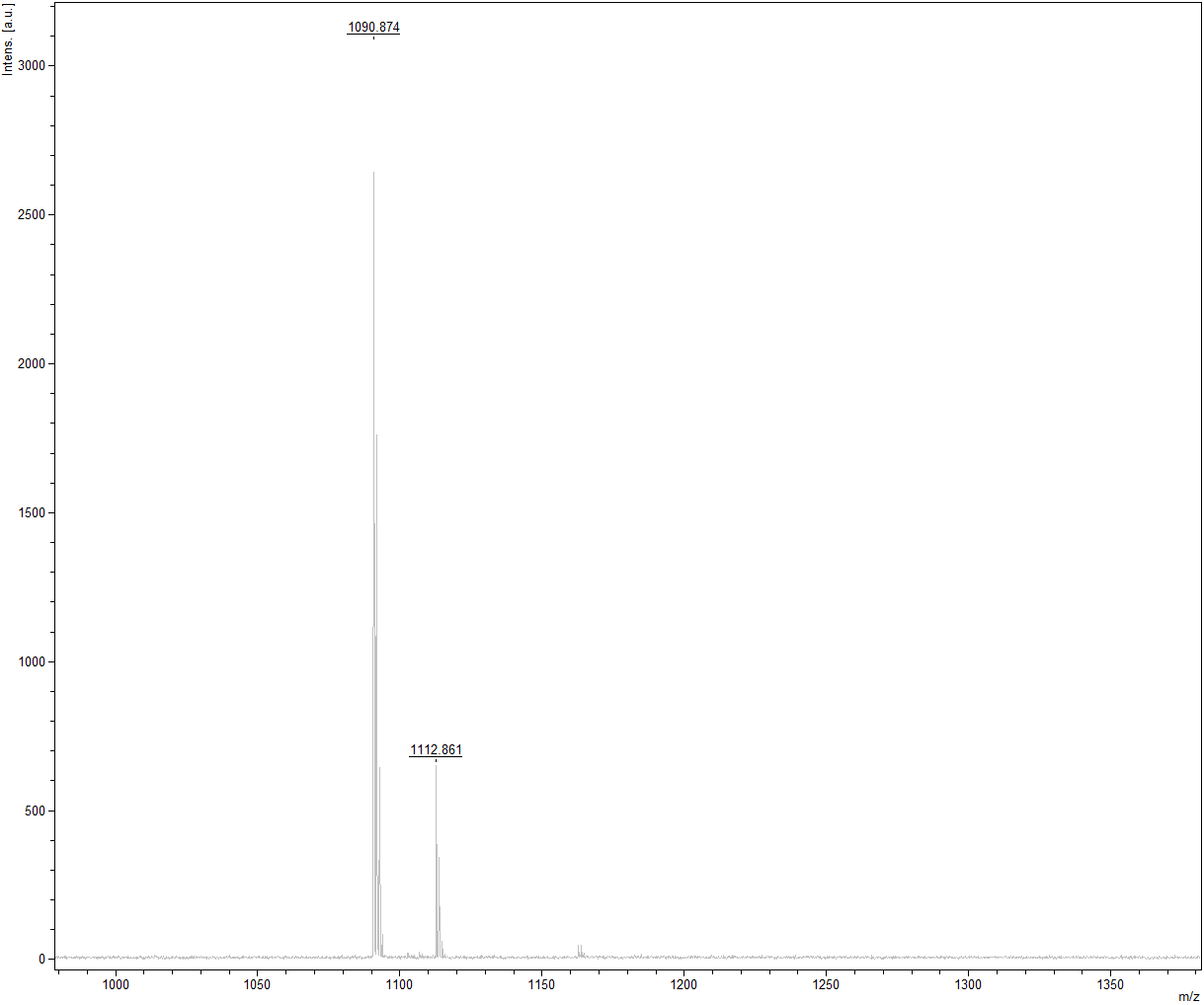


**Figure S141.** Mass spectrum of **A5-3B18**. MALDI-TOF MS m/z of [M + H]^+^ calculated for C_68_H_136_N_3_O_6_: 1091.038, Found: 1090.874.

**Figure S142.** ^1^H NMR of **A6-3B12**. ^1^H NMR (500 MHz, Chloroform-d) δ 4.06 (dt, *J* = 10.2, 6.8 Hz, 6H), 2.86 (t, *J* = 6.6 Hz, 2H), 2.76 (t, *J* = 7.3 Hz, 4H), 2.61 (t, *J* = 7.0 Hz, 2H), 2.54 – 2.38 (m, 8H), 1.65 – 1.53 (m, 8H), 1.37 – 1.15 (m, 54H), 0.88 (t, *J* = 6.9 Hz, 9H).


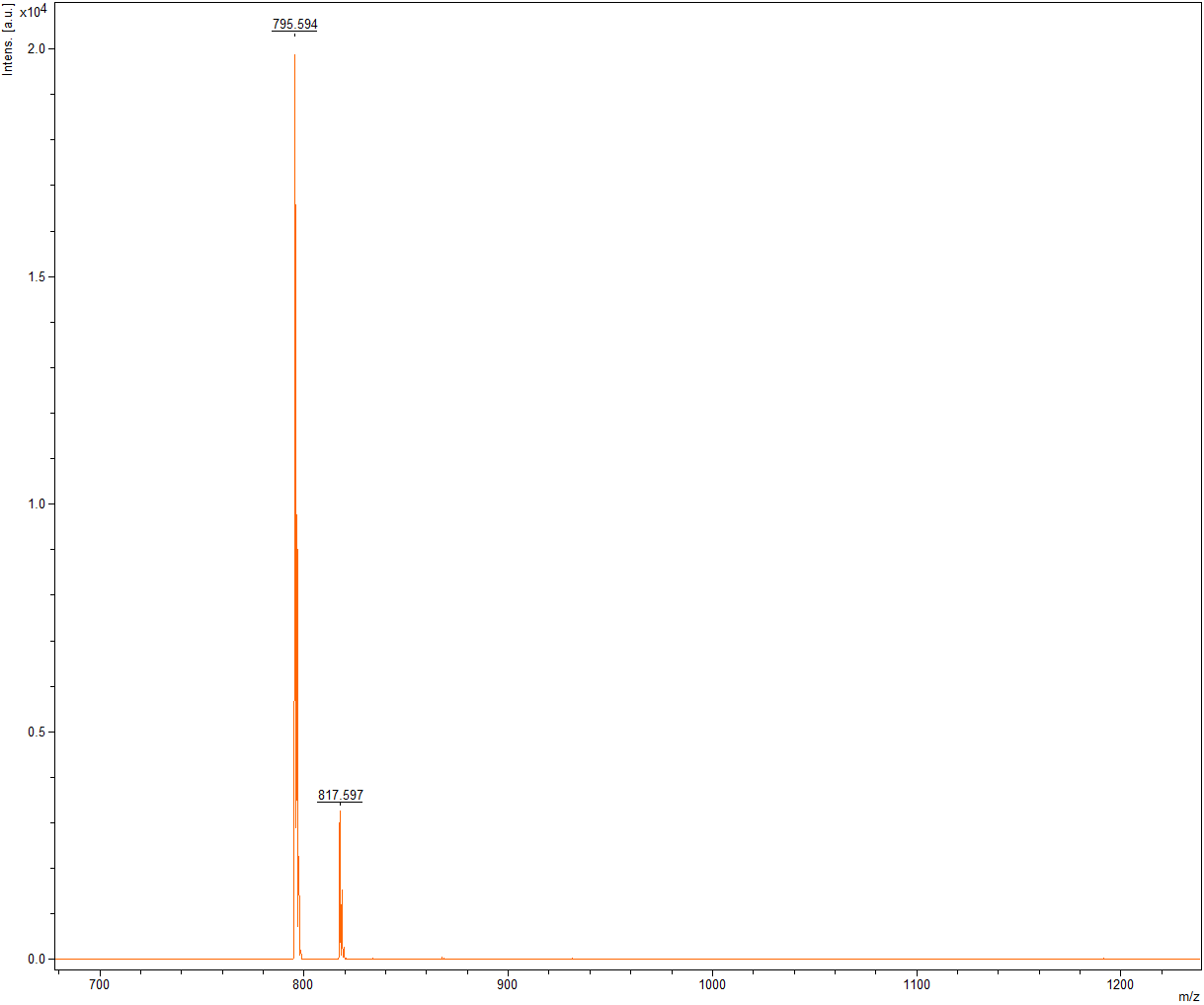


**Figure S143.** Mass spectrum of **A6-3B12**. MALDI-TOF MS m/z of [M + H]^+^ calculated for C_48_H_95_N_2_O_6_: 795.715, Found: 795.594.

**Figure S144.** ^1^H NMR of **A6-3B14**. ^1^H NMR (500 MHz, Chloroform-d) δ 4.06 (dt, *J* = 10.3, 6.8 Hz, 6H), 2.87 (t, *J* = 6.6 Hz, 2H), 2.76 (t, *J* = 7.3 Hz, 4H), 2.63 (t, *J* = 6.9 Hz, 2H), 2.56 – 2.37 (m, 8H), 1.61 (pd, *J* = 6.8, 2.2 Hz, 8H), 1.39 – 1.17 (m, 66H), 0.88 (t, *J* = 6.9 Hz, 9H).


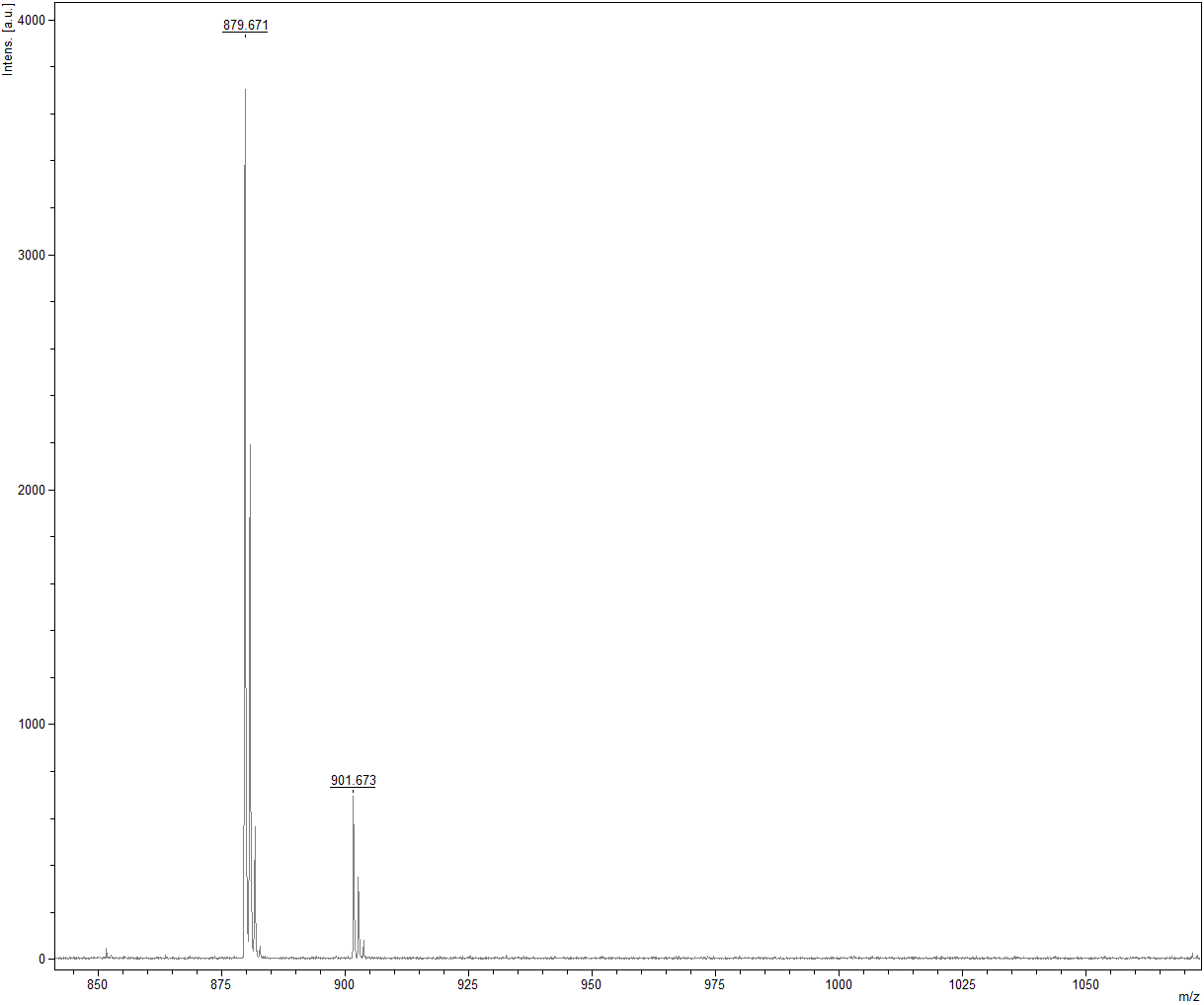


**Figure S145.** Mass spectrum of **A6-3B14**. MALDI-TOF MS m/z of [M + H]^+^ calculated for C_54_H_107_N_2_O_6_: 879.808, Found: 879.671.

**Figure S146.** ^1^H NMR of **A6-3B16**. ^1^H NMR (500 MHz, Chloroform-d) δ 4.06 (dt, *J* = 10.2, 6.8 Hz, 6H), 2.86 (t, *J* = 6.6 Hz, 2H), 2.76 (t, *J* = 7.3 Hz, 4H), 2.61 (t, *J* = 7.0 Hz, 2H), 2.55 – 2.37 (m, 8H), 1.61 (td, *J* = 7.2, 2.5 Hz, 8H), 1.33 – 1.23 (s, 78H), 0.88 (t, *J* = 6.9 Hz, 9H).


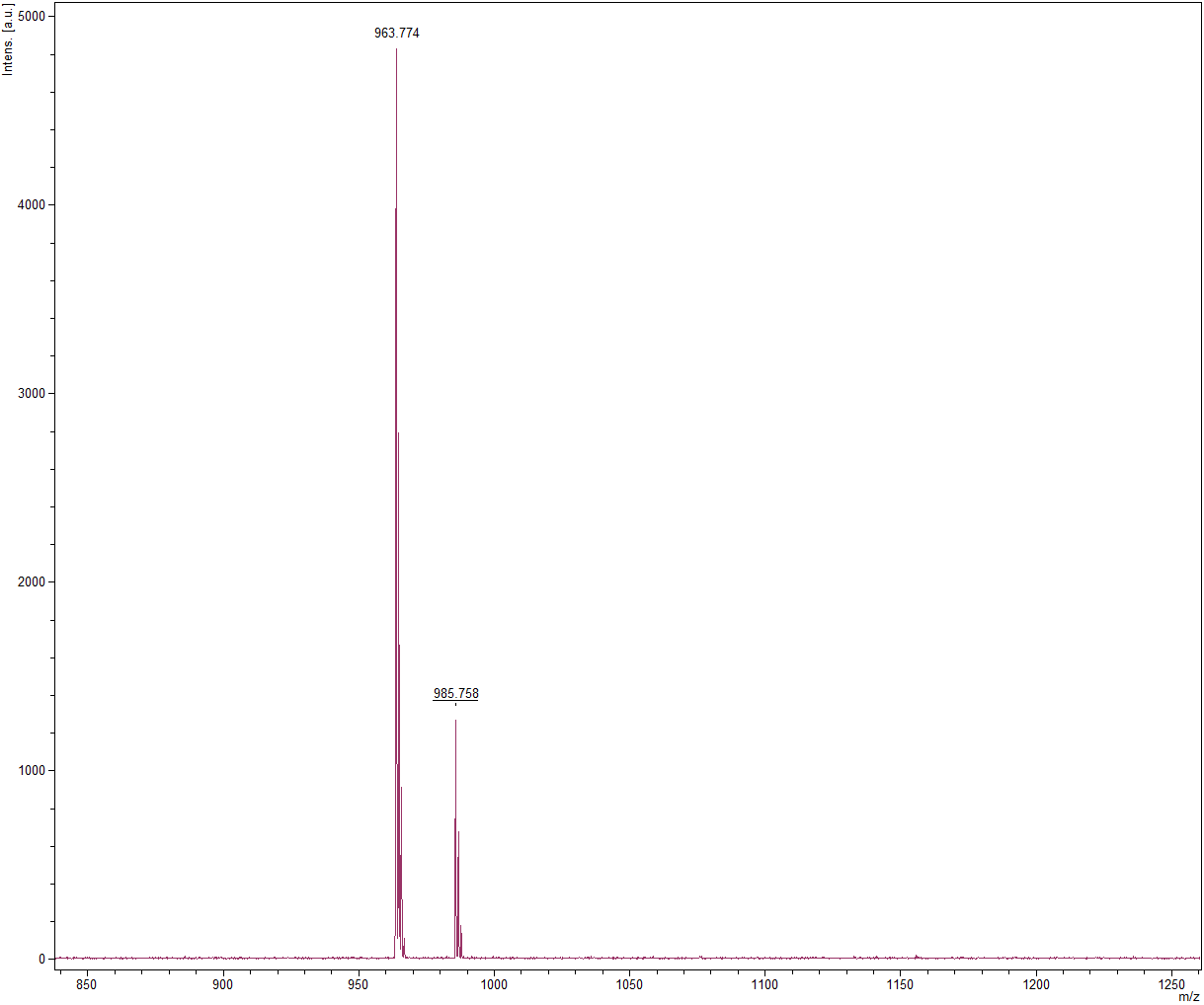


**Figure S147.** Mass spectrum of **A6-3B16**. MALDI-TOF MS m/z of [M + H]^+^ calculated for C_60_H_119_N_2_O_6_: 963.902, Found: 963.774.

**Figure S148.** ^1^H NMR of **A6-3B18**. ^1^H NMR (500 MHz, Chloroform-d) δ 4.06 (dt, *J* = 10.2, 6.8 Hz, 6H), 2.86 (t, *J* = 6.6 Hz, 2H), 2.76 (t, *J* = 7.3 Hz, 4H), 2.61 (t, *J* = 6.9 Hz, 2H), 2.55 – 2.38 (m, 8H), 1.61 (td, *J* = 7.2, 2.4 Hz, 8H), 1.35 – 1.23 (s, 90H), 0.88 (t, *J* = 6.9 Hz, 9H).


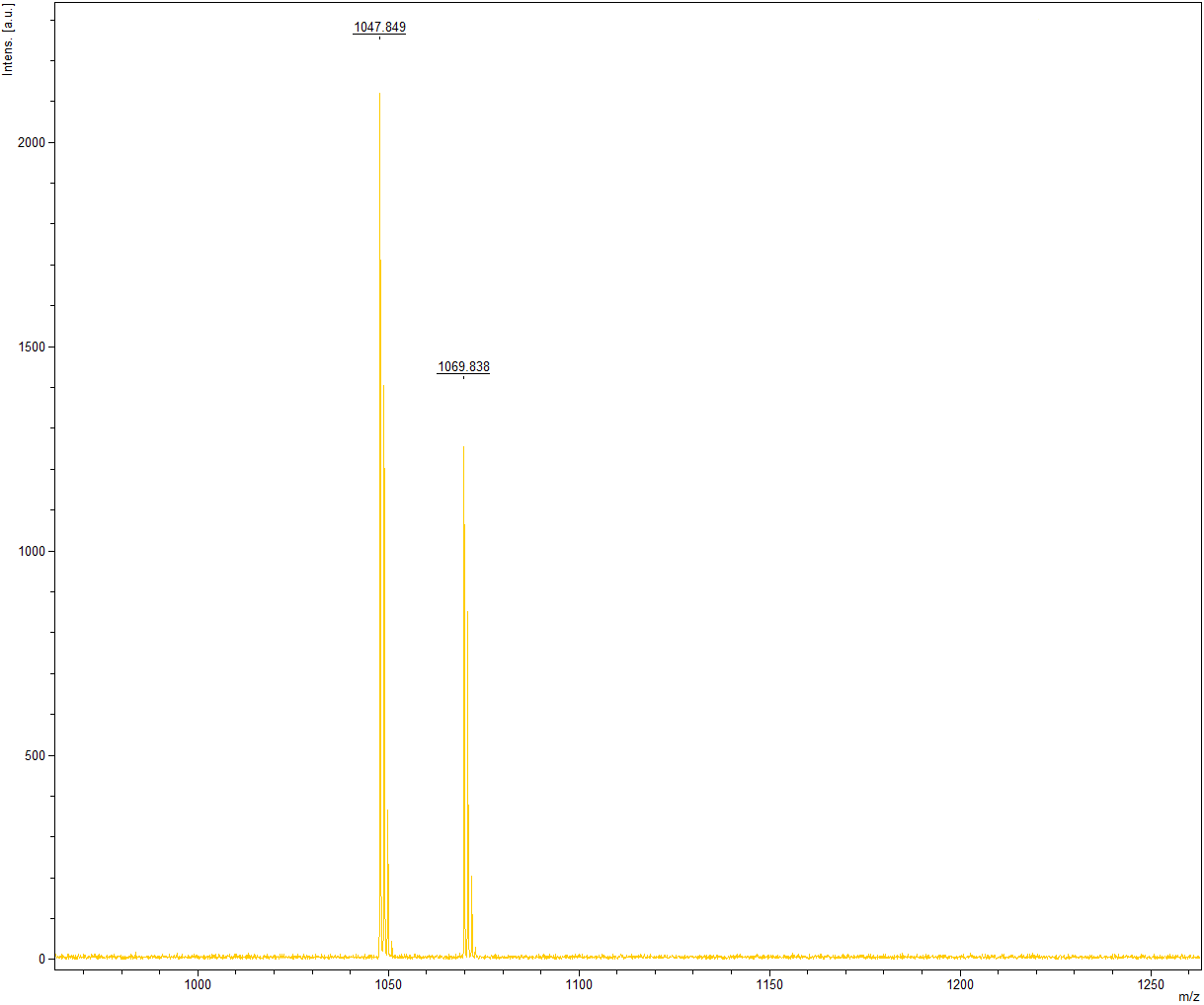


**Figure S149.** Mass spectrum of **A6-3B18**. MALDI-TOF MS m/z of [M + H]^+^ calculated for C_66_H_131_N_2_O_6_: 1047.996, Found: 1047.849.

**Figure S150.** ^1^H NMR of **A1-2O14-1B14**. ^1^H NMR (500 MHz, Chloroform-d) δ 4.06 (tt, *J* = 7.7, 6.2, 4.7 Hz, 2H), 3.71 – 3.47 (m, 10H), 3.22 – 2.20 (m, 12H), 1.71 – 1.12 (m, 68H), 0.88 (t, *J* = 6.9 Hz, 9H).


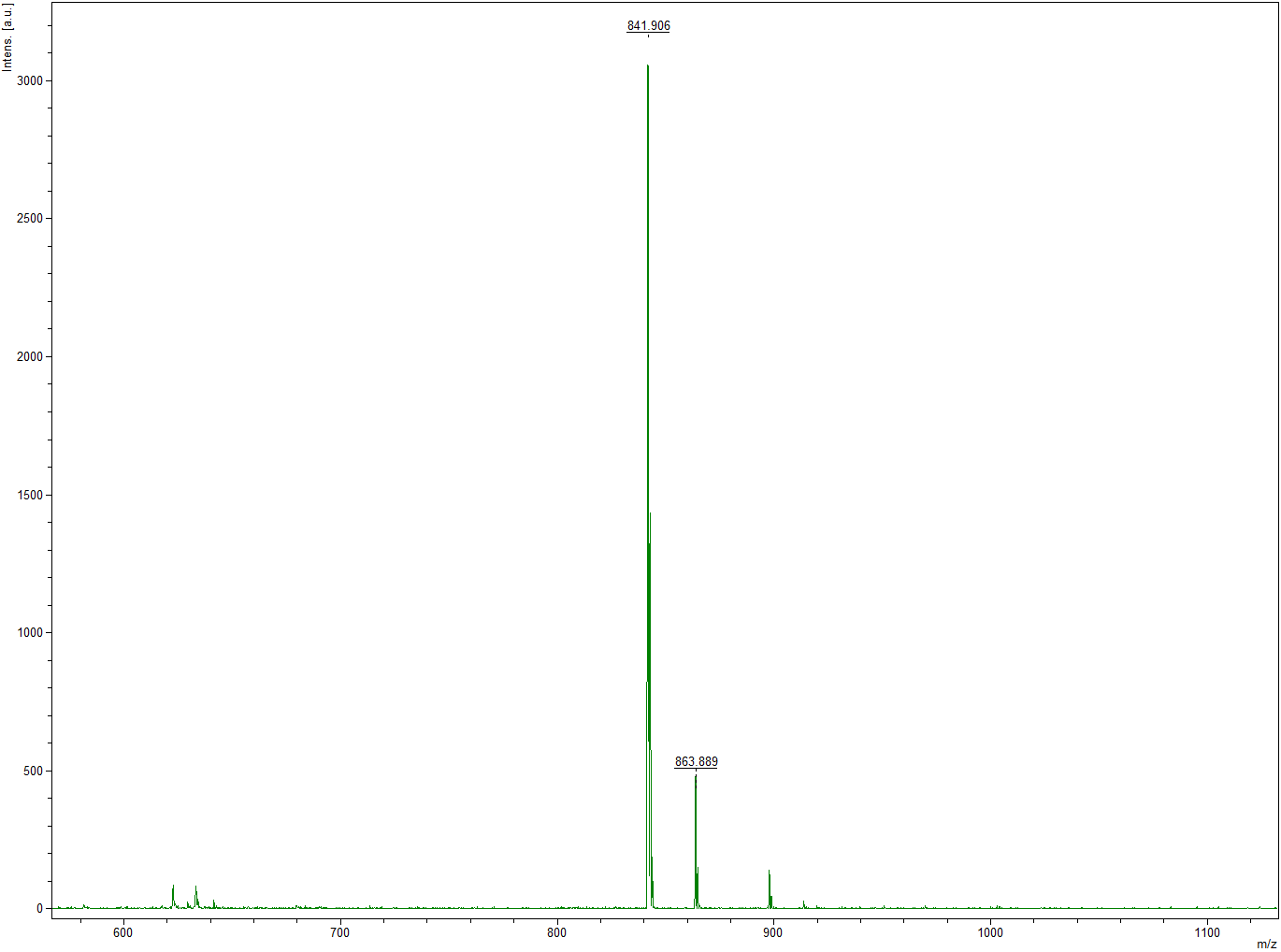


**Figure S151.** Mass spectrum of **A1-2O14-1B14**. MALDI-TOF MS m/z of [M + H]^+^ calculated for C_51_H_105_N_2_O_6_: 841.793, Found: 841.906.

**Figure S152.** ^1^H NMR of **A1-1O14-2B14**. ^1^H NMR (500 MHz, Chloroform-d) δ 4.06 (td, J = 6.8, 4.6 Hz, 4H), 3.68 – 3.47 (m, 9H), 3.12 – 2.19 (m, 14H), 1.61 (q, J = 6.9 Hz, 4H), 1.47 – 1.17 (m, 66H), 0.88 (t, J = 6.9 Hz, 9H).


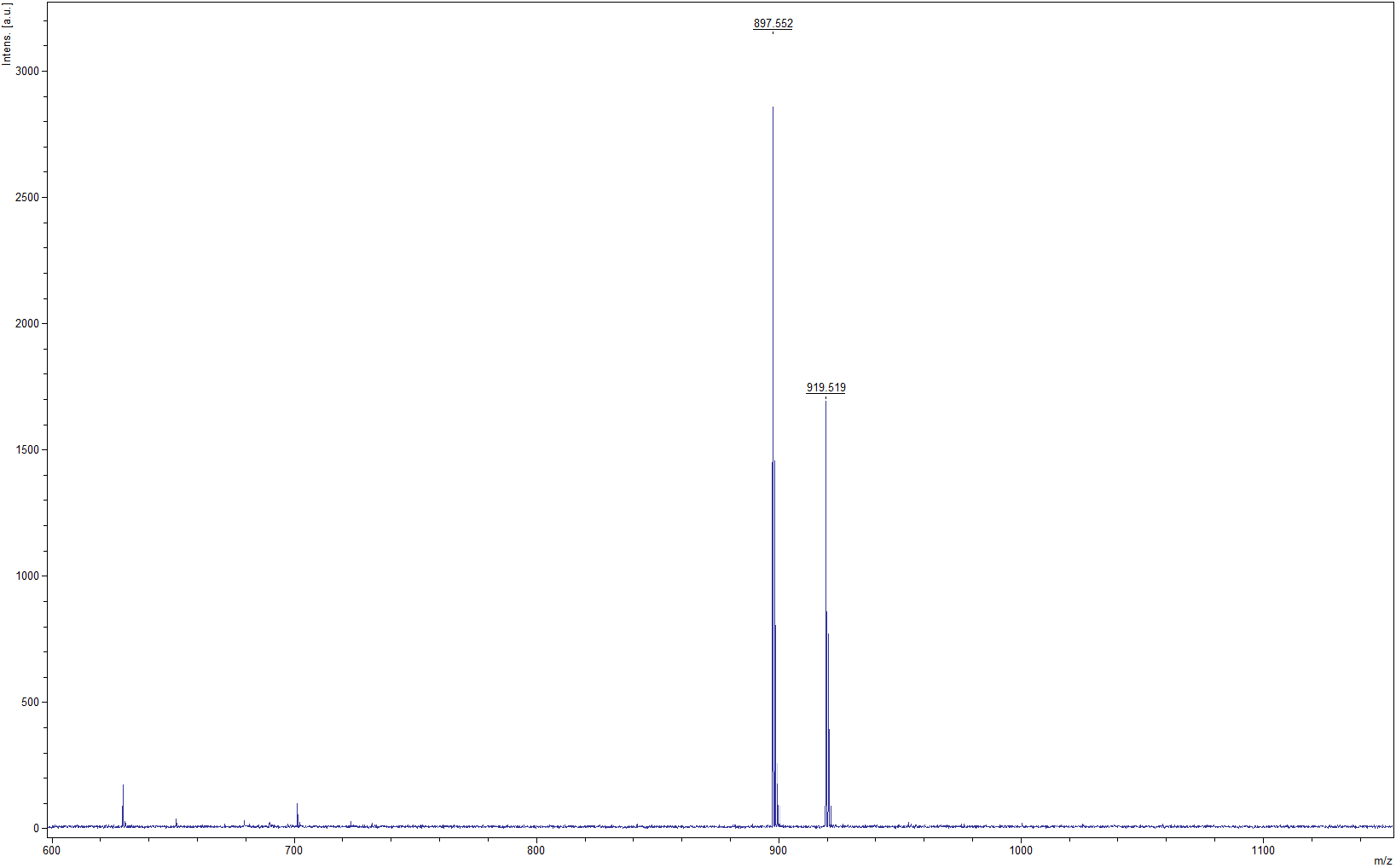


**Figure S153.** Mass spectrum of **A1-1O14-2B14**. MALDI-TOF MS m/z of [M + H]^+^ calculated for C_54_H_109_N_2_O_7_: 897.819, Found: 897.552.

**Supplementary figures and tables**

**Table S1.** The formulation applied for the preparation of A2-3O14 and the characterization of the corresponding LNPs. (n =3, technical replicates for the measurement of Z-Ave, PDI and Zeta in PBS).

| Formulation | **Molar % (molar ratios)** | | | | Z-Ave (nm) | PDI | Zeta in PBS (mV) | | references | |
| --- | --- | --- | --- | --- | --- | --- | --- | --- | --- | --- |
| A | | **A2-3O14** 50 | **DOPE** 10 | **Cholesterol**  38.5 | **DMG-PEG** 1.5 | 132.5±7.5 | 0.251±0.038 | | -3.54±2.06 |  |
| B | | **A2-3O14**  50 | **DSPC** 10 | **Cholesterol**  38.5 | **DMG-PEG** 1.5 | 128.4±0.4 | 0.16±0.008 | | -0.26±0.8 | [8]-[9] |
| C | | **A2-3O14**  35 | **DOPE** 16 | **Cholesterol**  46.5 | **DMG-PEG** 2.5 | 128.1±0.6 | 0.159±0.017 | | -2.23±0.18 | [10] |
| D | | **A2-3O14**  46.3 | **DSPC** 9.4 | **Cholesterol**  42.7 | **DMG-PEG** 1.6 | 144.6±2.4 | 0.204±0.011 | | -0.43±0.37 | [11] |


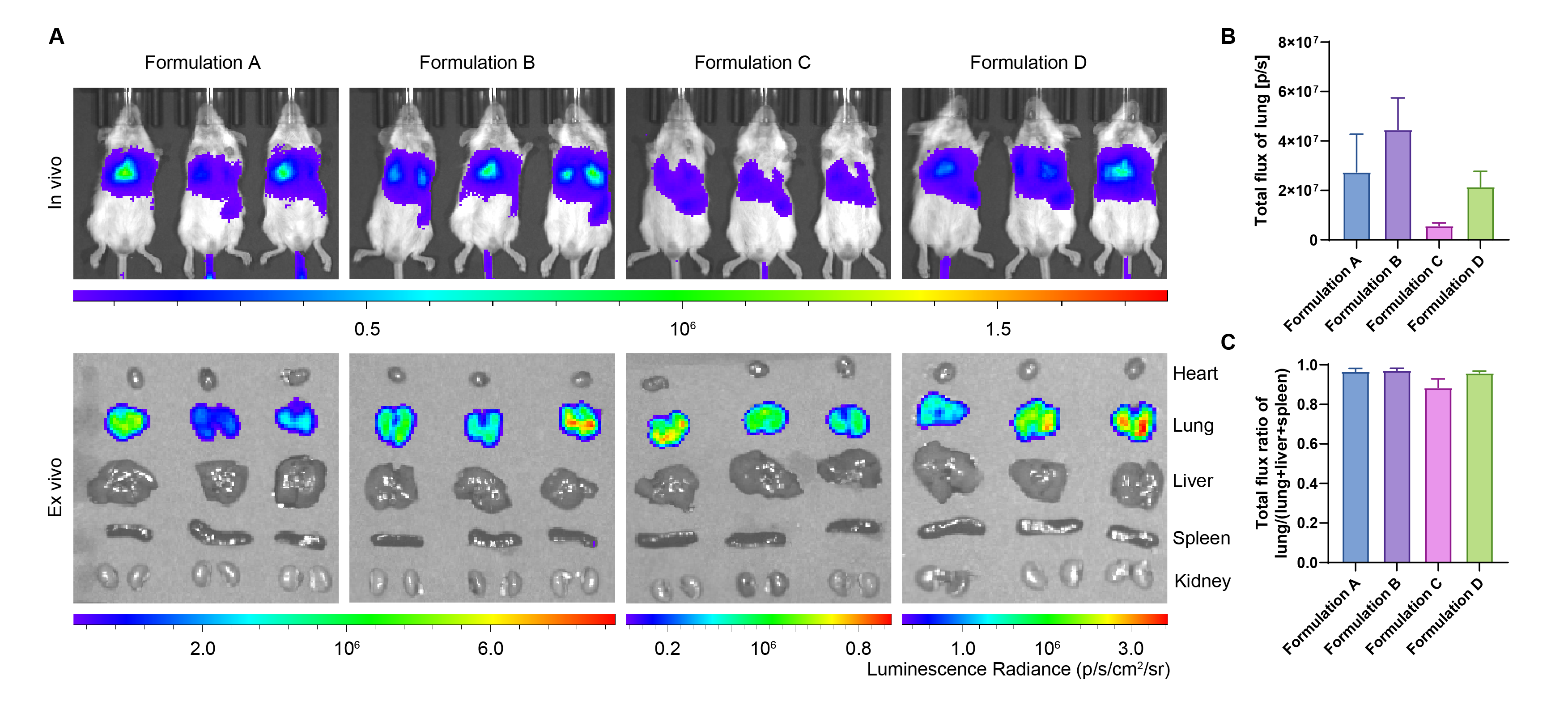


**Figure S154.** (A) Evaluation of the in vivo and ex vivo expression efficiency of Luc mRNA encapsulated by A2-3O14 LNPs with different formulations. The formulation of A, B, C and D were shown in Table S1. The mice were administrated with the Luc mRNA-loaded LNPs at a dose of 0.1 mg kg^-1^ through the tail vein. In/Ex vivo images were taken at 6 h post injection (n = 3). Data are presented as mean ± s.d. (B) The total flux of lung calculated from ex vivo images in A. (C) Calculate the total flux ratio in the lung by total flux of lung/(total flux of lung + total flux of liver + total flux of spleen) of the ex vivo images in A.


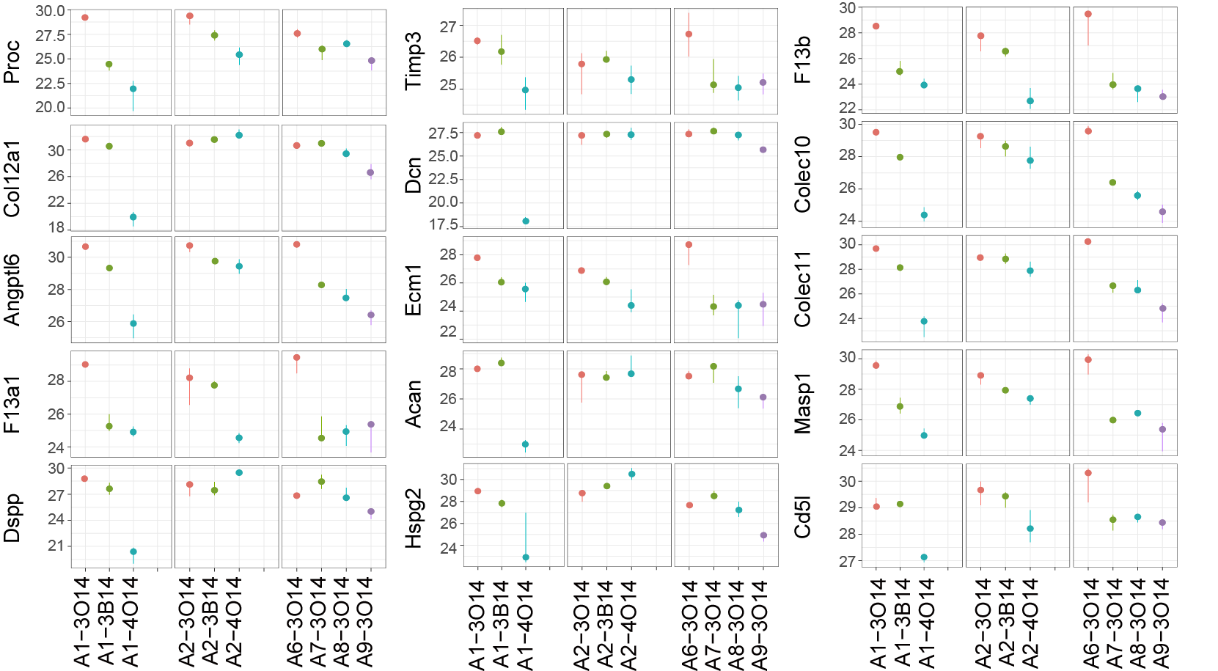


**Figure S155.** The abundance (log_2_ intensity) of the up-regulated proteins (lung-selective) in the protein coronas of the ten selected LNPs with varying degrees of lung-targeting ability (n = 3).


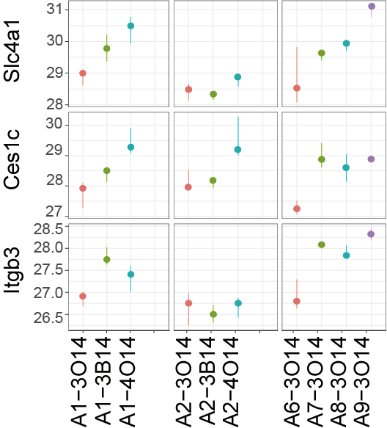


**Figure S156.** The abundance (log_2_ intensity) of the down-regulated proteins (liver-selective) in the protein coronas of the ten selected LNPs with varying degrees of lung-targeting ability (n = 3).

**Table S2.** The physicochemical properties, including pKa, Z-Ave, PDI, zeta potential in PBS and water, and TAR ((total flux of lung - total flux of liver)/(total flux of lung + total flux of liver)) of the LNPs. Additionally, logD and logP of the corresponding ionizable lipids were also listed in the table below. (n =3, technical replicates for the measurement of Z-Ave, PDI, Zeta in PBS and Zeta in ddH_2_O).

| Name | pKa | LogD | LogP | Z-Ave (nm) | PDI | Zeta in PBS (mV) | Zeta in ddH_2_O (mV) | TAR |
| --- | --- | --- | --- | --- | --- | --- | --- | --- |
| A1-3O10 | 5.34 | 4.93 | 6.99 | 124.5±11.7 | 0.31±0.024 | -0.02±0.36 | 15.7±4.35 | 0.97 |
| A1-3O12 | 6.26 | 7.6 | 9.37 | 166.1±61.7 | 0.287±0.013 | 0.48±0.58 | 15.37±1.31 | 0.72 |
| A1-3O14 | 5.34 | 10.27 | 11.75 | 187.8±5.6 | 0.243±0.029 | 0.38±0.33 | 14.53±1.34 | 0.95 |
| A1-3O16 | 5.78 | 12.94 | 14.13 | 161.1±4.1 | 0.292±0.01 | 0.9±0.36 | 15.73±0.29 | 0.75 |
| A1-3O18 | 5.66 | 15.6 | 16.51 | 178±2.8 | 0.311±0.01 | 1.97±0.27 | 18.17±1.36 | 0.80 |
| A1-4O10 | 5.59 | 8.53 | 9.87 | 183.9±2.6 | 0.103±0.029 | -6.24±0.85 | -17.4±1.71 | -0.95 |
| A1-4O12 | 5.65 | 12.09 | 13.04 | 209.4±1.2 | 0.21±0.013 | -2.94±0.91 | -14.87±2.91 | -0.98 |
| A1-4O14 | 5.59 | 15.64 | 16.21 | 277.1±18 | 0.263±0.022 | -8.11±1.66 | -23.93±2.3 | -0.98 |
| A1-4O16 | 5.65 | 19.2 | 19.38 | 458.1±121.2 | 0.465±0.093 | -11.33±1.17 | -28.1±2.43 | -0.95 |
| A1-4O18 | 5.41 | 22.76 | 22.55 | 244.6±3.7 | 0.294±0.018 | -12.04±2.94 | -17.13±1.1 | -0.97 |
| A1-3B12 | 5.36 | 11.11 | 11.84 | 137.9±3.5 | 0.201±0.011 | -2.56±0.6 | 5.07±0.35 | -0.37 |
| A1-3B14 | 5.37 | 13.78 | 14.22 | 117.4±1.7 | 0.154±0.007 | -0.84±0.18 | 6.36±0.16 | -0.17 |
| A1-3B16 | 5.05 | 16.45 | 16.6 | 116.3±0.5 | 0.158±0.016 | -4.4±0.98 | -7.69±0.65 | -0.78 |
| A1-3B18 | 6.00 | 19.11 | 18.98 | 133.9±2.1 | 0.145±0.013 | -1.59±1.74 | 4.46±0.66 | -0.96 |
| A2-3O10 | 7.52 | 4.46 | 7.41 | 115.1±1.2 | 0.342±0.014 | 0.65±0.83 | 14.4±0.61 | 0.98 |
| A2-3O12 | 6.19 | 7.13 | 9.78 | 124.4±6.2 | 0.246±0.061 | 0.72±0.33 | 16.9±0.87 | 0.98 |
| A2-3O14 | 6.40 | 9.79 | 12.16 | 134.1±1.7 | 0.217±0.01 | 0.83±0.7 | 12.27±1.51 | 0.98 |
| A2-3O16 | 5.75 | 12.46 | 14.54 | 160.3±10.4 | 0.252±0.008 | 1.39±0.81 | 18.1±0.79 | 0.98 |
| A2-3O18 | 5.47 | 15.13 | 16.92 | 146.6±1.6 | 0.177±0.028 | -0.31±0.21 | 14.73±0.57 | 0.97 |
| A2-4O10 | 7.69 | 8.02 | 10.28 | 130.3±1.3 | 0.206±0.01 | -0.49±0.48 | 8.11±0.41 | -0.83 |
| A2-4O12 | 6.20 | 11.57 | 13.46 | 118.5±2.5 | 0.206±0.023 | -3.36±3.62 | 6.75±1.74 | 0.05 |
| A2-4O14 | 5.42 | 15.13 | 16.63 | 142.3±19.7 | 0.299±0.124 | -2.52±0.55 | 10.22±0.4 | 0.24 |
| A2-4O16 | 5.17 | 18.69 | 19.8 | 187.5±9.9 | 0.274±0.009 | 0.32±0.13 | 13.43±0.5 | 0.79 |
| A2-4O18 | 5.21 | 22.24 | 22.97 | 237.1±14 | 0.284±0.023 | 0.17±0.09 | 13.47±0.67 | 0.98 |
| A2-3B12 | 5.30 | 10.63 | 12.26 | 137.2±4.3 | 0.107±0.015 | -0.91±0.57 | 7.52±0.25 | 0.88 |
| A2-3B14 | 5.59 | 13.3 | 14.63 | 130.3±8.3 | 0.277±0.033 | -0.42±0.56 | 18.33±0.57 | 0.95 |
| A2-3B16 | 5.72 | 15.97 | 17.01 | 122.5±7.1 | 0.215±0.016 | -0.4±0.24 | 16.23±0.81 | 0.91 |
| A2-3B18 | 5.22 | 18.63 | 19.39 | 240±1.9 | 0.266±0.014 | -1.03±0.38 | 12.7±0.17 | 0.88 |
| A3-3O10 | 7.89 | 4.51 | 7.21 | 123.5±1.8 | 0.342±0.021 | -0.05±0.25 | 6.44±0.34 | 0.85 |
| A3-3O12 | 7.75 | 7.18 | 9.59 | 125.4±8.7 | 0.251±0.017 | -0.47±0.17 | 5.63±0.88 | 0.95 |
| A3-3O14 | 7.10 | 9.85 | 11.96 | 169±2.2 | 0.255±0.009 | -0.27±0.12 | 11.07±0.21 | 0.95 |
| A3-3O16 | 7.09 | 12.51 | 14.34 | 193.5±2.1 | 0.22±0.025 | -0.45±0.39 | 10.47±0.15 | 0.80 |
| A3-3O18 | 7.37 | 15.18 | 16.72 | 203.1±6.5 | 0.197±0.018 | -1.15±0.61 | 11.63±0.06 | 0.80 |
| A5-3O10 | 7.15 | 4.92 | 7.3 | 317.1±88.9 | 0.379±0.057 | 2.33±0.4 | 12.07±2.04 | 0.91 |
| A5-3O12 | 5.31 | 7.58 | 9.68 | 125.4±2.3 | 0.312±0.029 | 2.84±0.45 | 14.77±1.7 | 0.69 |
| A5-3O14 | 5.99 | 10.25 | 12.06 | 110.9±13.1 | 0.28±0.022 | 1.83±0.58 | 14.1±0.36 | 0.92 |
| A5-3O16 | 5.90 | 12.92 | 14.44 | 116.2±1.1 | 0.208±0.009 | 0.6±0.63 | 15.43±0.8 | 0.87 |
| A5-3O18 | 5.70 | 15.59 | 16.81 | 154.3±5.3 | 0.288±0.035 | 3.56±0.92 | 19.13±0.67 | 0.91 |
| A5-4O10 | 4.12 | 8.87 | 10.18 | 145.8±1.9 | 0.24±0.024 | -3.51±3.22 | 5.67±0.63 | -0.98 |
| A5-4O12 | 4.56 | 12.43 | 13.35 | 153.2±1.5 | 0.177±0.031 | -1.13±0.67 | 5.82±1.26 | -0.98 |
| A5-4O14 | 3.27 | 15.99 | 16.52 | 119.3±6.2 | 0.303±0.033 | 0.51±0.28 | 9.33±1.02 | -0.85 |
| A5-4O16 | 4.25 | 19.54 | 19.69 | 188.4±115.8 | 0.325±0.06 | 0.12±0.49 | 4.34±1.18 | -0.15 |
| A5-4O18 | 4.02 | 23.1 | 22.86 | 157.6±6.9 | 0.226±0.049 | 2.51±0.38 | 4.64±1.3 | 0.18 |
| A5-3B12 | 6.62 | 11.08 | 12.15 | 119.8±12.8 | 0.299±0.072 | -0.8±0.28 | 5.45±0.8 | 0.46 |
| A5-3B14 | 5.54 | 13.74 | 14.53 | 152.7±59.8 | 0.261±0.014 | -0.99±0.51 | 9.55±0.41 | 0.72 |
| A5-3B16 | 5.39 | 16.41 | 16.91 | 113.8±1.8 | 0.21±0.011 | -0.71±0.57 | 13.83±1.42 | 0.88 |
| A5-3B18 | 5.18 | 19.08 | 19.28 | 140.5±0.9 | 0.196±0.01 | -0.57±0.42 | 16.97±0.95 | 0.91 |
| A6-3O10 | 5.96 | 5.89 | 7.38 | 104.9±4.9 | 0.272±0.045 | -1.03±0.51 | 10.62±1.28 | -0.37 |
| A6-3O12 | 6.06 | 8.55 | 9.75 | 119.2±2.4 | 0.177±0.03 | -1.09±0.19 | 12.57±0.93 | 0.79 |
| A6-3O14 | 5.60 | 11.22 | 12.13 | 142.7±6.2 | 0.218±0.034 | -0.91±0.96 | 10.27±0.21 | 0.88 |
| A6-3O16 | 3.56 | 13.89 | 14.51 | 200.8±3.5 | 0.177±0.005 | -0.24±0.16 | 13.57±0.51 | 0.73 |
| A6-3O18 | 4.21 | 16.56 | 16.89 | 816.3±55.8 | 0.399±0.107 | -0.3±0.16 | 10.05±0.38 | -0.02 |
| A7-3O10 | 7.21 | 6.64 | 7.74 | 150.4±1.7 | 0.107±0.012 | -3.76±1.04 | 3.36±0.63 | -0.95 |
| A7-3O12 | 6.73 | 9.30 | 10.12 | 113.5±2.7 | 0.207±0.006 | -5.64±2 | -11.03±2.65 | -0.84 |
| A7-3O14 | 7.02 | 11.97 | 12.49 | 124.6±6.2 | 0.254±0.038 | -0.75±0.36 | 7.98±2.02 | 0.52 |
| A7-3O16 | 5.79 | 14.64 | 14.87 | 130.4±3.2 | 0.177±0.028 | -5.76±1.01 | 8.8±1.19 | 0.35 |
| A7-3O18 | 5.18 | 17.31 | 17.25 | 176.9±3.1 | 0.282±0.004 | -5.74±0.49 | 9.5±1.04 | 0.50 |
| A8-3O14 | 6.65 | 12.84 | 12.15 | 115.5±1.5 | 0.24±0.019 | -5.44±1.33 | 11.53±1.15 | -0.33 |
| A9-3O14 | 6.34 | 13.3 | 12.46 | 170.9±1.5 | 0.254±0.003 | -5.54±0.43 | -2.84±0.27 | -0.86 |
|  |  |  |  |  |  |  |  |  |


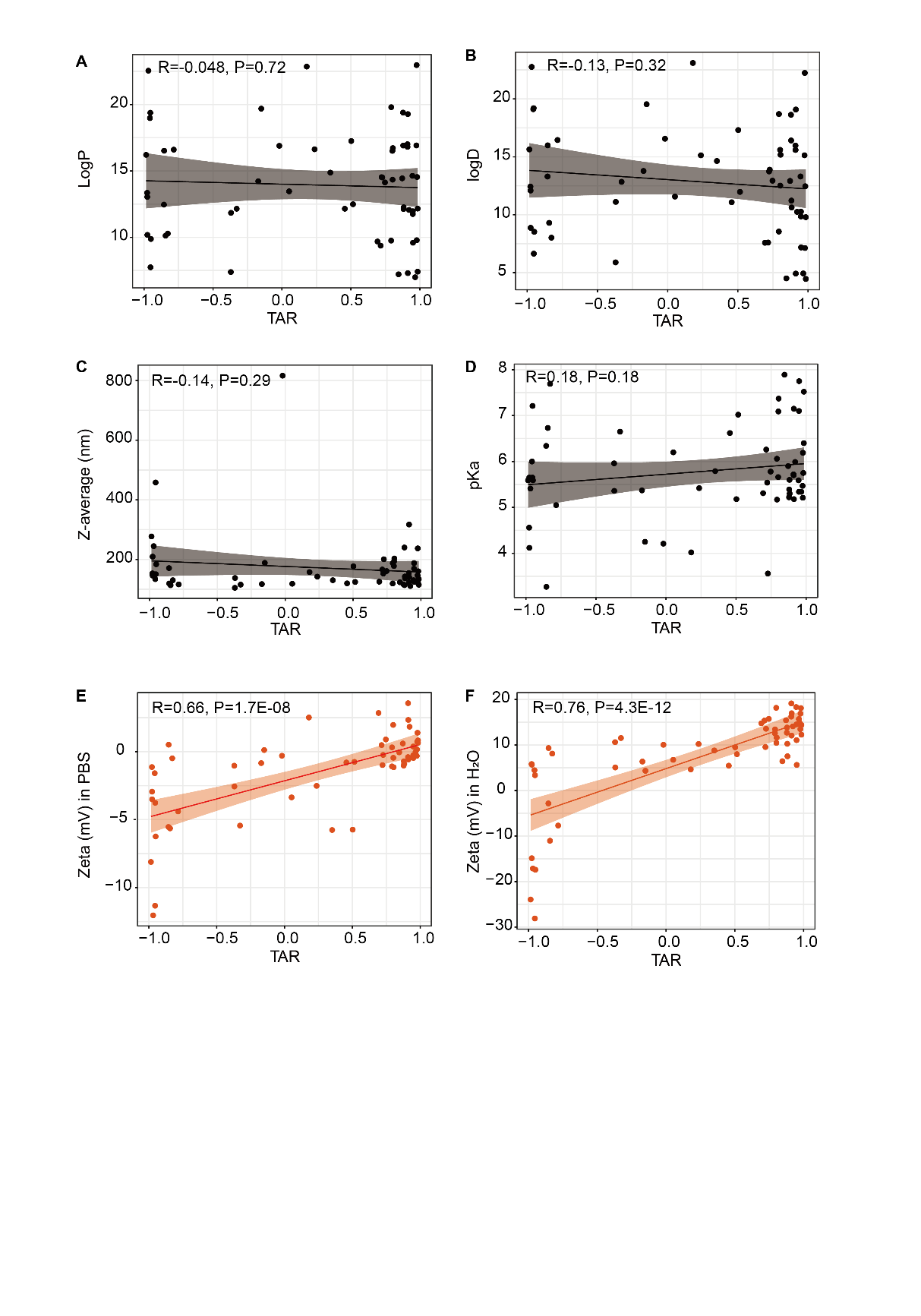


**Figure S157.** The correlation between TAR (total flux of lung - total flux of liver)/(total flux of lung + total flux of liver)) and the physicochemical properties including (A) LogP (B) LogD, (C) Z-average (nm), (D) pKa, (E) Zeta in PBS (mV) and (F) Zeta in H_2_O (mV), of the LNPs listed in Table S1.


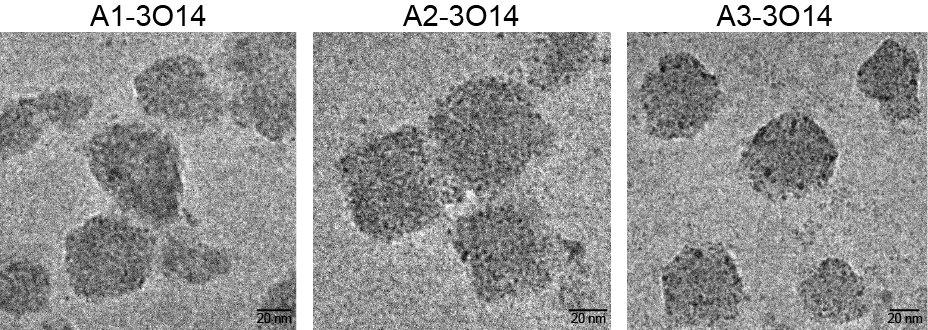


**Figure S158.** TEM images of the **A1-3O14** LNPs, **A2-3O14** LNPs, and **A3-3O14** LNPs.

**References**

[1] M. Kim, M. Jeong, S. Hur, Y. Cho, J. Park, H. Jung, Y. Seo, H. A. Woo, K. T. Nam, K. Lee, H. Lee, *Sci. Adv.* **2021**, *7(9):*eabf4398.

[2] Y. Cao, Z. He, Q. Chen, X. He, L. Su, W. Yu, M. Zhang, H. Yang, X. Huang, J. Li, *Nano Lett.* **2022**, *22(16):*6580-6589.

[3] X. Zhang, A. H. Smits, G. B. van Tilburg, H. Ovaa, W. Huber, M. Vermeulen, *Nat. Protoc.* **2018**, *13(3):*530-550.

[4] Z. Feng, P. Fang, H. Zheng, X. Zhang, *Bioinformatics* **2023**, *39 (8):*btad526.

[5] M. E. Ritchie, B. Phipson, D. Wu, Y. Hu, C. W. Law, W. Shi, G. K. Smyth, *Nucleic Acids Res.* **2015**, *43(7):*e47.

[6] Z. Gu, L. Gu, R. Eils, M. Schlesner, B. Brors, *Bioinformatics* **2014**, *30(19):*2811-2812.

[7] C. Ginestet, *J. R. Stat. Soc. Ser. A-Stat. Soc.* **2011**, *174(1):*245-245.

[8] K.J. Hassett, K.E. Benenato, E. Jacquinet, A. Lee, A. Woods, O. Yuzhakov, S. Himansu, J. Deterling, B.M. Geilich, T. Ketova, C. Mihai, A. Lynn, I. McFadyen, M.J. Moore, J.J. Senn, M.G. Stanton, Ö. Almarsson, G. Ciaramella, L.A. Brito, *Mol. Ther. Nucleic Acids.* **2019**, *15(15)*:1-11.

[9] A. Akinc, M.A. Maier, M. Manoharan, K. Fitzgerald, M. Jayaraman, S. Barros, S. Ansell, X. Du, M.J. Hope, T.D. Madden, B.L. Mui, S.C. Semple, Y.K. Tam, M. Ciufolini, D. Witzigmann, J.A. Kulkarni, R. van der Meel, P.R. Cullis, *Nat. Nanotechnol.* **2019**, *14(12)*:1084-1087.

[10] K.T. Love, K.P. Mahon, C.G. Levins, K.A. Whitehead, W. Querbes, J.R. Dorkin, J. Qin, W. Cantley, L.L. Qin, T. Racie, M. Frank-Kamenetsky, K.N. Yip, R. Alvarez, D.W. Sah, A. de Fougerolles, K. Fitzgerald, V. Koteliansky, A. Akinc, R. Langer, D.G. Anderson, Lipid-like materials for low-dose, in vivo gene silencing. *Proc. Natl. Acad. Sci. U S A.* **2010**,*107(5)*:1864-1869.

[11] L. Schoenmaker, D. Witzigmann, J.A. Kulkarni, R. Verbeke, G. Kersten, W. Jiskoot, D.J.A. Crommelin, mRNA-lipid nanoparticle COVID-19 vaccines: Structure and stability. *Int. J. Pharm.* **2021**, *601*:120586.
